# Epigenetic aging during the COVID-19 pandemic: A prospective longitudinal study with prepandemic baseline data in children, adolescents, and adults

**DOI:** 10.1073/pnas.2604211123

**Published:** 2026-11-03

**Authors:** Alicia M. Schowe, Mirko Ruks, Charlotte K. L. Disselkamp, Dmitry V. Kuznetsov, Jana Instinske, Theresa Rohm, Andreas J. Forstner, Markus M. Nöthen, Bastian Mönkediek, Christian Kandler, Darina Czamara, Elisabeth B. Binder

**Affiliations:** ^a^Department of Genes and Environment, Max-Planck-Institute of Psychiatry, Munich 80804, Germany; ^b^Graduate School of Systemic Neuroscience, Ludwig-Maximilians Universität, Planegg 82152, Germany; ^c^ Faculty of Sociology, Bielefeld University, Bielefeld 33615, Germany; ^d^ Department of Psychology, Bielefeld University, Bielefeld 33615, Germany; ^e^ Institute of Human Genetics, School of Medicine and University Hospital Bonn, University of Bonn, Bonn 53127, Germany; ^f^ Center for Environmental Neuroscience, Max Planck Institute for Human Development, Berlin 14195, Germany; ^g^ Department of Psychology, University of Bremen, Bremen 28359, Germany; ^h^Institute of Neuroscience and Medicine, Research Center Jülich, Jülich 52428, Germany; ^i^ Joint Institute for Individualisation in a Changing Environment, University of Münster University, Münster 48149, Germany; ^j^ Joint Institute for Individualisation in a Changing Environment, University of Bielefeld University, Bielefeld 33615, Germany

**Keywords:** biological aging, epigenetic clocks, COVID-19 pandemic, pandemic stressors, adolescence

## Abstract

Psychosocial stress is a risk factor for aging-related diseases. The COVID-19 pandemic placed a psychosocial burden on individuals, potentially increasing the risk for aging-related diseases for which epigenetic clocks are used as early biomarkers. Based on four epigenetic clocks (DunedinPACE, GrimAge2, Horvath, and PedBE), we investigated whether epigenetic aging changed during the pandemic and whether these changes were associated with objective and subjective pandemic-related stressors and preexisting individual risk factors for poorer physical and mental health. We used prospective longitudinal data (n = 1,028), including repeated salivary DNA collection from children (n = 359, MAGE = 9.5 y), adolescents (n = 385, MAGE = 15.5 y), and adults (n = 284, MAGE = 24.3 y). Saliva was collected before the first phase of containment measures in Germany in March 2020 and after the second phase in 2021. We observed significant increases in epigenetic age acceleration (PedBE: b = 0.17, *P* < 0.0001; GrimAge2: b = 0.08, *P* < 0.0001) and pace of aging (DunedinPACE: b = 0.13, *P* < 0.0001) across all age groups. Prepandemic physical health and parental socioeconomic status, but not objective pandemic stressors, were significantly associated with this change. In adolescents, higher perceived pandemic burden was associated with a larger increase in pace of aging (DunedinPACE: b = 0.04, *P* = 0.01). Together, these findings suggest that prepandemic individual risk factors for poorer physical and mental health are accompanied by measurable increases in epigenetic aging during the COVID-19 pandemic, with adolescence representing a sensitive phase to its psychosocial burden.

## Significance

Large-scale societal disruptions may have lasting impact on health outcomes, yet evidence linking such events to biological alterations is limited. Using prospective, longitudinal data spanning childhood to adulthood, we show that epigenetic aging increased from before to during the COVID-19 pandemic. These changes were associated with physical health, socioeconomic factors, and—among adolescents—perceived pandemic burden. Our findings are consistent with the possibility that societal crises are accompanied by measurable biological changes mediated through multifactorial pathways. They also suggest adolescence as a particularly sensitive developmental period for the pandemic-related increased psychosocial burden. Long-term follow-up will be needed to confirm whether these changes persist and predict adverse health outcomes later in life.

Individuals of the same chronological age can differ in their risk for aging-related health outcomes, such as cognitive decline and cardiometabolic health associated with premature mortality (1–4). While chronological age progresses the same for every individual, the rate of aging-related molecular, cellular, functional, and physiological changes can vary substantially between individuals and can be used to quantify an individual’s biological age (5).Over the past decade, epigenetic clocks have emerged as promising molecular measures of biological aging, capturing aging-related variation in DNA methylation (DNAm) patterns across the genome. They are computed as the weighted sum of DNAm levels at Cytosine-phosphate-Guanine (CpG) dinucleotides associated with chronological age or age-related phenotypes. Across different epigenetic clocks, accelerated epigenetic aging (where epigenetic age exceeds chronological age) has been linked to poorer physical health, aging-related disease, and increased mortality later in life (6–10). Epigenetic clocks may thus serve as early biological signature of modifiable disease risk before aging-related diseases become clinically apparent (5, 9). Given projections that the global burden of disease will increasingly shift toward aging-related noncommunicable diseases (11), identifying early biological signatures of modifiable risk can have broad implications for public health, health economics, and social policy. This may be particularly relevant in the context of global health crises, such as the covid virus disease 2019 (COVID-19) pandemic, for which long-term disease outcomes cannot be assessed in a timely fashion. Epigenetic clocks may therefore provide valuable means of assessing early risk and evaluating the potential long-term impact of societal measures on later health outcomes.

In addition, as detailed below, there is evidence that the COVID-19 pandemic introduced psychosocial strain in people’s lives and worsened stress-related mental health, both of which have previously been associated with accelerated epigenetic aging. Indeed, stress-related mental disorders (e.g., major depressive or anxiety disorders) and psychosocial stress exposure (e.g., financial strain, social isolation) have been associated with an increased risk for aging-related diseases (1–4) and accelerated epigenetic aging (6–10) outside the COVID-19 context. As a globally shared and recent multifaceted crisis, the COVID-19 pandemic combined direct health threats with large-scale disruptions to economic activity, education, social relationships, and mobility, placing strains on individuals worldwide across nearly every aspect of their daily life (12, 13). Several of these strains, most notably financial hardship and social isolation, are well-established psychosocial stressors (1, 2, 14) and a large body of literature highlights an increase in anxiety and depressive symptoms during the first lockdown of the pandemic in Germany (March to July 2020)(15), with some studies linking symptom severity to 7-d incidence rates and policy stringency (16–18). At the same time, behavioral and lifestyle adaptations to the pandemic-related restrictions led to reduction in physical activity and increased weight gain among both youth and adults (19–22). The presence of more severe anxiety and depressive symptoms and higher perceived pandemic burden has been linked to several environmental, developmental, clinical, and genetic factors (18, 23–25). The most affected groups included children and adolescents, females, individuals of lower socioeconomic status (SES), those with poorer prepandemic mental health, a lower polygenic score for subjective well-being, and those subjected to stricter physical distancing measures (18, 23–25). While these mental health and behavioral effects have been documented across large-scale meta-analyses spanning sample sizes from approximately 7,000 to over 3.5 million participants, evidence for biological alterations over the course of the COVID-19 pandemic remains sparse. So far, circulating stress hormones (26, 27) and fasting glucose, lipids, ferritin, and pro-inflammatory cytokines (28) have been shown to increase in three adolescent samples (n of 44 to 77). In adults, four of five studies found significant increases in circulating stress hormones (n of 87 to 827) (29–33). Meanwhile, epigenetic changes have only been related to postacute COVID-19 infection symptoms (PACS) and long-COVID (34). This highlights a critical gap in understanding if and how the COVID-19 pandemic and its societal implication may be associated with aging-related epigenetic alterations that have been linked to long-term disease risk.

**Fig. 1.**
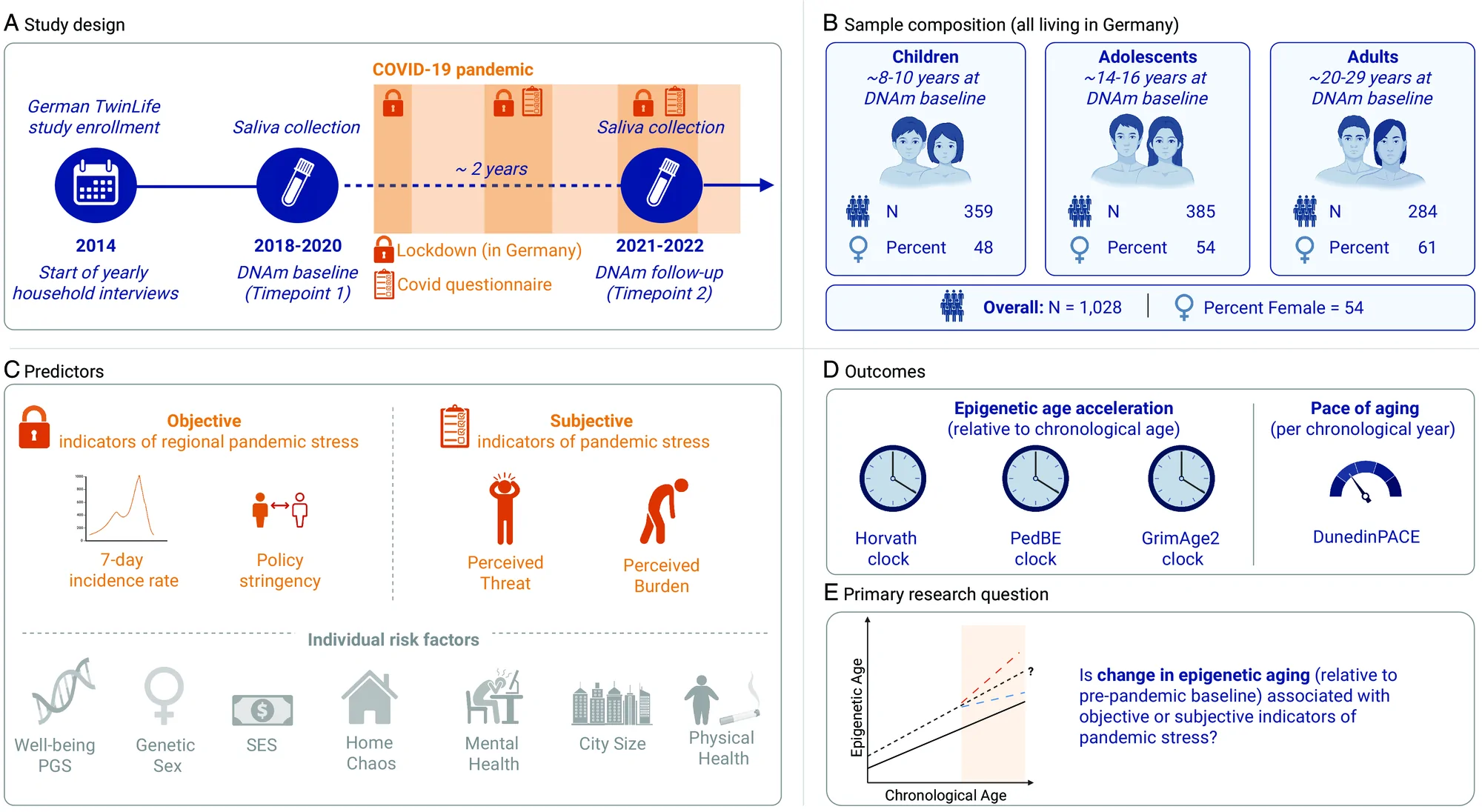
Conceptual overview illustrating the study design (*A*), the sample composition (*B*), the examined predictors (*C*) and outcomes (*D*), and main research question (*E*). Panel *A* shows the study design from the initiation of the TwinLife study in 2014 up to the second saliva collection, with the COVID-19 pandemic in Germany (1st of March 2020 to 5th of May 2023) highlighted in orange. All families participated in yearly household interviews. The first saliva sample was collected through trained interviewers during household interviews. For this study, we excluded the 18 individuals for whom saliva was collected after the 1st of March 2020. A second saliva sample was collected from TwinLife participants via a self-collection kit according to written instructions and mailed back between September 2021 and May 2022. The pandemic unfolded through three main lockdown periods in Germany (indicated by the lock and shaded orange areas). During two of the lockdowns, participants filled in additional questionnaires on their perception of the pandemic. A detailed illustration of all repeated measures is provided in *SI Appendix*, section 2.6 and Fig. S9. Panel *B* illustrates the sample composition (Table 1). Panel *C* shows all tested predictors, with pandemic stressors highlighted in orange as the main predictors of interest in the present study. Subjective indicators of pandemic stress were derived from the COVID-19 supplementary surveys (i.e., computer-assisted telephone or web interviews), including questions on the subjective burden and threat due to the COVID-19 pandemic, home environment, and anxiety symptoms (*SI Appendix*, sections 2.8 and 2.9). During the yearly household interviews (*SI Appendix,* Fig. S1) before the pandemic, TwinLife families provided information on their socioeconomic background, home environment, body-mass-index, and mental health (Table 2). Their salivary DNA was used for genotyping and DNAm-based estimation of physical health including BMI and smoking exposure (*SI Appendix*, sections 2.2 and 2.3). They also provided their post codes for regional characterization including city size, regional COVID-19 7-d incidence rates, and regional governmental stringency of imposed containment measures (*SI Appendix*, sections 2.6 and 2.7). Two chronological (Horvath and PedBE clock) and two biological (GrimAge2 clock, DunedinPACE) epigenetic clocks shown in panel *D* were computed from salivary DNAm (*SI Appendix*, section 2.4) to quantify the study outcome variables. Panel *E* illustrates the main analysis of the present study, in which changes in epigenetic age acceleration (or pace of aging) from before to during the COVID-19 pandemic were examined in relation to objective and subjective indicators of pandemic stress. In addition, associations with prepandemic individual risk factors for higher pandemic stress were explored (for a more detailed overview of tested associations, see *SI Appendix*, Fig. S10). BMI = body-mass-index, DNAm = DNA methylation, PedBE = pediatric-buccal-epigenetic clock. Created with BioRender.com.

A major obstacle in testing for biological changes in the context of the pandemic and stress research more broadly is the scarcity of longitudinal biological data with a pre- and postexposure assessment combined with deep phenotyping. To our knowledge, the German longitudinal TwinLife Epigenetic Change Satellite (TECS) project is the only prospective within-person DNAm dataset to date spanning prepandemic and pandemic periods (total n = 1,028). It includes repeated salivary DNAm samples from children (age range: 8.6 to 10.2 y), adolescents (age range: 14.8 to 16.2 y), and adults (age range: 20.8 to 29.2 y) (Table 1) (35, 36). Saliva collections took place before the first lockdown in March 2020 and after the second lockdown in 2021 in Germany (Fig. 1*A*). For the present study, we leveraged this design in which the COVID-19 pandemic constituted a natural experiment occurring between repeated assessments and, thus, were able to test whether a major, globally shared stressor was associated with detectable epigenetic changes.

**Table 1.** Analytic sample

	All, n = 1,028	Children, n = 359	Adolescents, n = 385	Adults, n = 284
Chronological age prepandemic baseline [years], M (SD)	15.82 (6.03)	9.51 (0.32)	15.47 (0.31)	24.28 (3.05)
Chronological age pandemic follow-up [years], M (SD)	18.15 (6.06)	11.84 (0.58)	17.76 (0.57)	26.63 (3.12)
Genetic sex [female], N (%)	550 (54%)	171 (48%)	206 (54%)	173 (61%)
Parental highest education^*^ [years], M (SD)	15.63 (2.84)	16.37 (2.70)	15.55 (2.74)	14.72 (2.92)
Parental net HH income^†^ [Euro], M (SD)	2,168.47 (1,026.70)	2,480.29 (1,133.86)	1,890.97 (846.63)	2,144.76 (996.08)
City size				
<19,999 inhabitants, N (%)	211 (21%)	79 (22%)	75 (19%)	57 (20%)
20,000 to 99,999 inhabitants, N (%)	310 (30%)	115 (32%)	122 (32%)	73 (26%)
100,000 to 499,999 inhabitants, N (%)	229 (22%)	63 (18%)	85 (22%)	81 (29%)
>500,000 inhabitants, N (%)	278 (27%)	102 (28%)	103 (27%)	73 (26%)
Ever-infected HH at pandemic follow-up^‡^ [yes], N (%)	94 (9.1%)	34 (9.5%)	33 (8.6%)	27 (9.5%)
Fully vaccinated at pandemic follow-up^§^ [yes], N (%)		NA	334 (87%)	264 (93%)
Any cardiometabolic diagnosis, [yes], N (%)^¶^	20 (1.9%)	0 (0%)	8 (2.1%)	12 (4.2%)
Any mental disorder diagnosis, [yes], N (%)^¶^	30 (2.9%)	0 (0%)	20 (5.2%)	10 (3.5%)

Note: HH = household, SD = standard deviation.

^*^Information missing for 30 participants (2 children, 28 adults).

^†^Information missing for 25 participants (6 children, 12, adolescents, 7 adults).

^‡^Self-reported COVID-19 infection of any household member, information missing for 11 children.

^§^Information missing for 416 participants (all 359 children, 38 adolescents, and 19 adults). From June 7th, 2021, anyone older than 12 y could get vaccinated.

^¶^Information missing for 98 participants (all 24 children, 45 adolescents, and 29 adults).

Specifically, we tested whether epigenetic age acceleration and pace of aging changed during the pandemic and whether these changes were associated with pandemic-related stressors. As detailed in Fig. 1*C* and Table 2, we used two objective and two subjective indicators of pandemic-related stress in our analysis: the regional 7-d incidence rate and policy stringency in participant’s residential area and the self-reported perceived pandemic threat and burden (*SI Appendix*, sections 2.6–2.8). In addition, we explored whether changes in epigenetic age acceleration and pace of aging were associated with environmental, developmental, clinical, and genetic factors that may contribute to more severe stress experiences and that have been previously linked to poorer physical health and more severe anxiety and depressive symptoms during the COVID-19 pandemic (18, 23–25). As described in Table 2, these included indicators of demographic background (sex, parental, and individual SES), home environment (living on their own (for adults), population size of residential city, perceived home chaos), mental health (depressive and anxiety symptoms), a polygenic score-based liability for subjective well-being (PGS for well-being), and physical health (BMI, DNAm-based scores for BMI and smoking).

**Table 2.** Brief overview of measurements

Construct	Specific variables	Description
Biological aging	**Epigenetic Clocks^a,e^:** Horvath DNAmAge PedBE clock GrimAge2 DunedinPACE	Epigenetic clocks were calculated as the weighted sum of salivary DNAm at CpG sites. Horvath DNAmAge, the PedBE clock, and GrimAge2 represent epigenetic age in years. Positive residuals of epigenetic age regressed on chronological age indicate epigenetic age acceleration (as epigenetic exceeds chronological age). DunedinPACE indicates the rate of aging, with 1 = the same rate as chronological aging.
Indicators of pandemic stressors	**Subjective^a,d^:** Perceived pandemic threat^c,d^ Perceived pandemic burden^c,d^ **Objective^b-e^:** 7-d incidence rate Policy stringency	Pandemic threat and burden were rated on 4 to 7 items (depending on scale, age group, lockdown) from 1 to 10. 7-d incidence rate (i.e., COVID-19 cases of the last 7 d/the number of inhabitants multiplied by 100,000) and policy stringency (i.e., restrictions across 21 areas of daily life rated and averaged from 0 to 100) for each participant’s place of residence were quantified using the AUC.
Covariates	**Model 1:** Chronological age^a,e^ Genetic sex^a^ Cell type^a,e^ Genetic relatedness^a^ **In addition, in Model 2:** BMI^a^ Epigenetic smoking^a,e^	Age was calculated from the birth and saliva sampling date and BMI as kg/m^2^. Genetic sex and relatedness were based on genotypes. Cell type proportions were estimated from salivary DNAm. Smoking exposure was estimated based on reversely coded beta values of cg05575921, with higher values indicating greater smoking exposure.
Demographic	Genetic sex^a^ History of SES^a^ For adults: personal SES^a^	Genetic sex: see covariates. History of parental SES was estimated using the average sum of the maximum years of parental education, occupational prestige, and net household income. For adults who had left the parental home, we additionally considered personal SES in the same manner.
Home environment	Subjective home environment^a^ City size^a–e^ Living at parental home^a^	The home environment was rated on 5 to 6 items (depending on age) from 1 to 5, with higher scores indicating a more chaotic home. City size of participant’s place of residents was coded into four categories: inhabitants <19,999; 20,000 to 99,999; 100,000 to 499,999; >500,000).
PGS-derived genetic liability	PGS for subjective well-being^a^	Individual genotypes were used to compute the PGS for subjective well-being.
Mental health	Depression^a^ Anxiety^a,c,d^	Anxiety (2 items) and depressive (7 items) symptom frequency was rated from 1 to 4.
Physical health	BMI^a^ Epigenetic BMI^a,e^ Epigenetic smoking^a,e^	BMI, smoking: see covariates. Epigenetic BMI was calculated from salivary DNAm.

Note: Please consult the *SI Appendix*, section 2, for detailed information. Variables were assessed at: ^a^prepandemic baseline, ^b^first, ^c^second, or ^d^third lockdown ^e^pandemic follow-up. BMI = body-mass-index, SES = socioeconomic status, DNAm = DNA methylation, PGS = polygenic score.

To quantify epigenetic aging, we used four epigenetic clocks. Epigenetic clocks differ in their training models, CpG selection, source tissues, and age ranges (37, 38), representing complementary but conceptually distinct frameworks for quantifying disease risk. “Chronological” clocks primarily predict chronological age, representing how DNAm patterns (in years) accumulate over the lifespan, whereas “biological” clocks integrate biomarkers tied to mortality and morbidity such as inflammation or blood lipids, indicating cumulative biological age (in years). An additional approach was taken with DunedinPACE, which was trained on longitudinally measured biomarkers of aging-related deterioration and estimates the pace of current aging in years of biological change per chronological year (39). Because DNAm is sensitive to tissue and cell type composition, for our study, it was important to select epigenetic clocks that perform well in saliva, across childhood, adolescence, and adulthood, and that have been shown to be associated with adverse environmental exposure in this tissue. We selected two chronological clocks, Horvath DNAmAge (40) and the Pediatric-Buccal-Epigenetic (PedBE) clock (41) and two biological clocks, GrimAge2 (42) and DunedinPACE (39). Horvath DNAmAge was trained across several tissues including saliva and covered a large age range (1 to 101 y). PedBE was trained on buccal cells in youth (0 to 20 y of age). Both clocks tracked chronological age well in the saliva samples in TECS [(35) and *SI Appendix*, sections 2.4 and 2.5] and prior studies using salivary DNA have shown their association with psychosocial stress (43). GrimAge2 was also highly correlated with chronological age in TECS (r = 0.80). While both GrimAge2 and DunedinPACE have been trained in adult blood, recent cross-tissue analyses indicate that adversity-related effects generalize from blood to saliva within the same child, with lower SES consistently associated with higher GrimAge2 acceleration and faster DunedinPACE-measured pace of aging (44). Importantly, these cross-tissue analyses suggest that accurate chronological age prediction is not the best indicator of epigenetic sensitivity to adversity or disease risk and rather emphasize the utility of GrimAge2 acceleration and DunedinPACE (6–9), even when measured in saliva (44).

## Results

Demographic information of the analytic sample is summarized in Table 1. The objective measures of pandemic stress, reflected in the 7-d incidence rate and the policy stringency index of government-imposed containment measures encountered by our analytic sample, closely followed the nationwide trends (TECS: median AUC of 7-DIR AUC = 65,445 and policy stringency = 30,085; Germany: median AUC of 7-d incidence rate = 65,761 and policy stringency = 30,122; *SI Appendix,* Figs. S7 and S8). On the subjective level, TECS participants perceived the COVID-19 pandemic as low to moderately threatening and burdensome, with average scores during the second lockdown and the third lockdown between 3 and 5 (total range: 1 to 10). Descriptive statistics of the subjective and objective pandemic stressors and more detailed information on the course of the COVID-19 pandemic in Germany can be found in *SI Appendix*, sections 2.6 and 4.1, respectively.

### Epigenetic Change During the COVID-19 Pandemic.

**Fig. 2.**
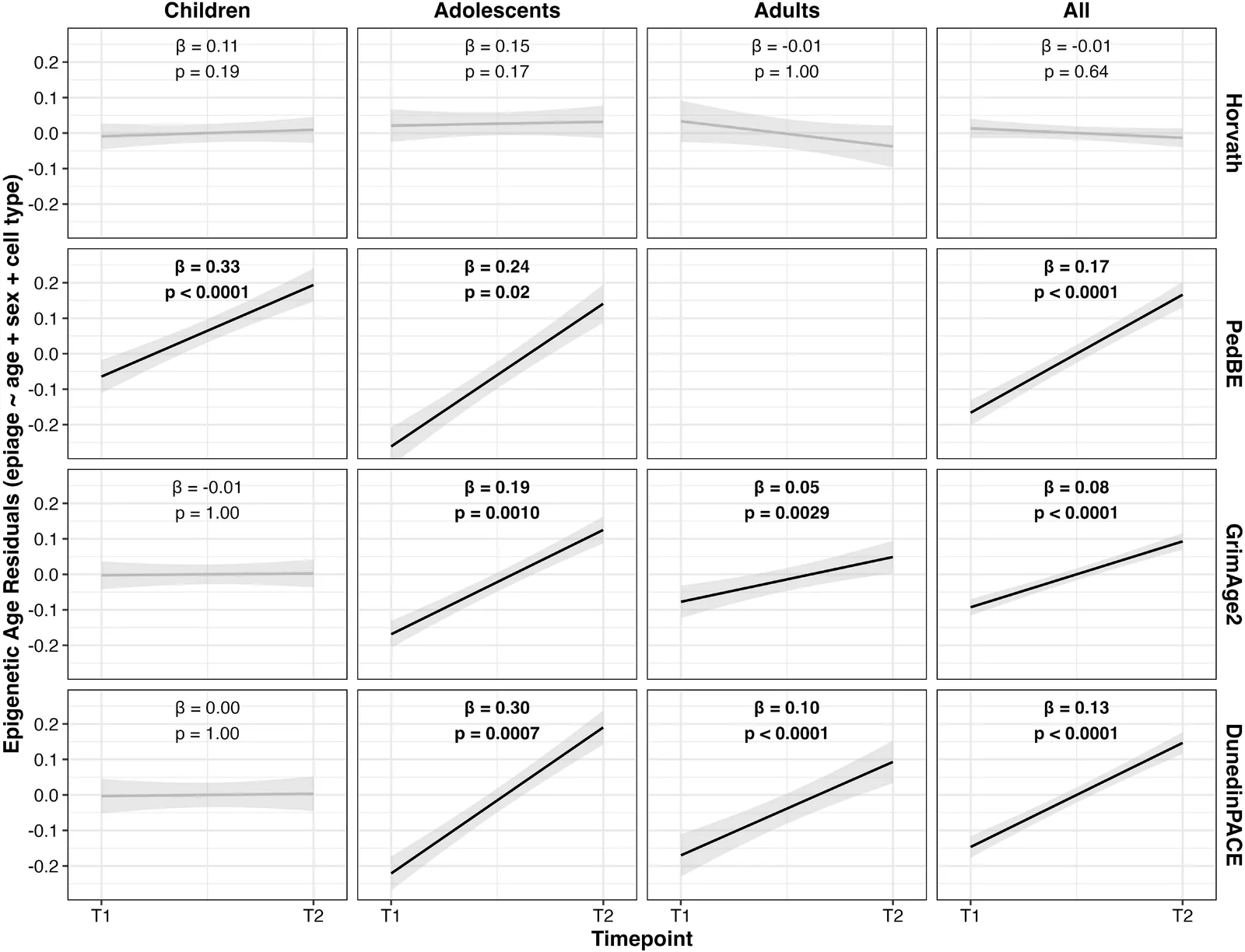
Average Change in Epigenetic Aging across Individuals. Average change in epigenetic age and pace of aging (regressed on chronological age, sex, and cell type) from before the COVID-19 pandemic (T1: December 2018 to February 2020) to pandemic follow-up (T2: 1.5 to 3 y later, from September 2021 to May 2022) during the third lockdown for children, adolescents, adults, and the full sample (“All”). Horvath DNAmAge, the PedBE clock, and GrimAge2 represent z-score transformed epigenetic age measured in years regressed on age, sex, and cell type. DunedinPACE, on the other hand, represents the pace of aging where a value of 1 represents a rate of aging at the same pace as chronological age. To be consistent across clocks, we also show the z-score transformed pace of aging residualized for age, sex, and cell type. Gray areas indicate the SE. PedBE was not analyzed for adults and thus left blank (*SI Appendix*, section 2.5). Full sample analysis for PedBE refers to children and adolescents combined. Beta coefficients indicate change in epigenetic age in SD per year. *P*-values displayed in the figure are corrected for multiple testing (*SI Appendix*, section 3.2, M_eff_ = 4). Significant changes are highlighted in bold.

We tested for change in epigenetic aging using four separate linear mixed models, with the two repeated measures of each epigenetic clock as outcome and time between the two assessments as predictor (*Materials and Methods*). Multiple testing was corrected for by multiplying the *P*-value by the effective number of independent tests (n = 4). As illustrated in Fig. 2, all clocks, except Horvath DNAmAge, showed a significant increase in age acceleration or pace of aging from prepandemic baseline to pandemic follow-up (Horvath: *β* = -0.01, *p.adj* = 0.64; PedBE: *β* = 0.17, *p.adj* < 0.0001; GrimAge2: *β* = 0.08, *p.adj* < 0.0001; DunedinPACE: *β* = 0.13, *p.adj* < 0.0001). These findings remained statistically significant when controlling for BMI and smoking exposure and when excluding households ever affected by viral infection (*SI Appendix*, section 4.2). Our age-stratified analyses showed that, in children, only PedBE age acceleration (*β* = 0.33, *p.adj* < 0.0001) increased significantly. The findings for adolescents and adults were similar to those for the full sample, with an increase in acceleration in all examined clocks except for Horvath (adolescents: PedBE: *β* = 0.24, *p.adj* = 0.02, GrimAge2: *β* = 1.19, *p.adj* = 0.001, DunedinPACE: *β* = 0.30, *p.adj* = 0.0007; adults: GrimAge2: *β* = 0.05, *p.adj* = 0.003; DunedinPACE: *β* = 0.10, *p.adj* < 0.0001; PedBE was originally developed in children and adolescents, hence we did not test it in adults).

### Epigenetic Change in Relation to Pandemic Stressors.

**Fig. 3.**
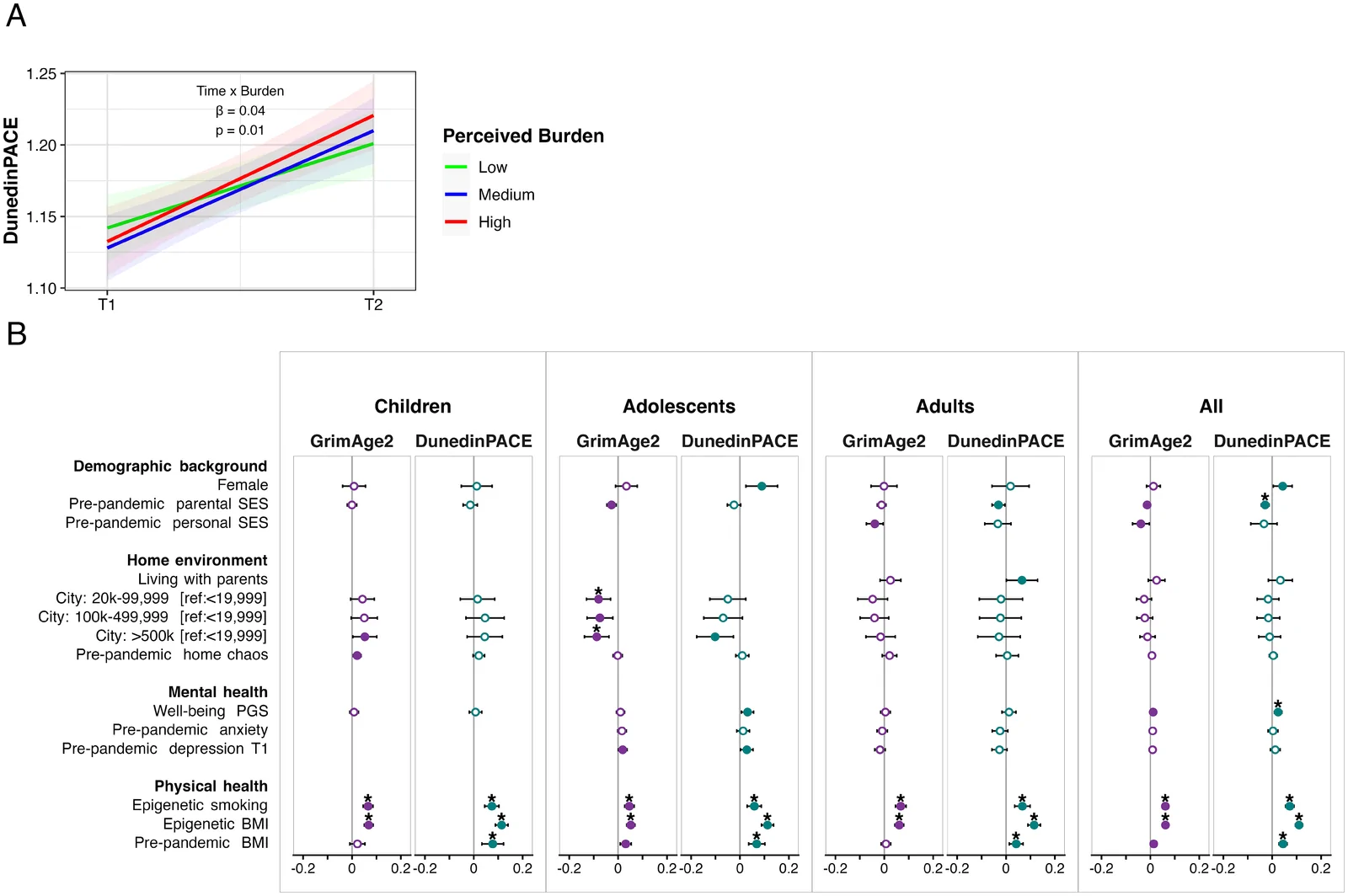
Associations of perceived pandemic burden and individual risk factors with change in epigenetic aging in biological clocks. Panel *A* shows the pace of aging for adolescents with low (green line, *n* = 129, M = 2.12, SD = 0.52), medium (blue line, *n* = 128, M = 3.60, SD = 0.55), and high (red line, *n* = 128, M = 6.02, SD = 1.06) levels of perceived burden during the third lockdown. The forest plot in panel *B* shows the standardized beta and 95% CI of the time x predictor interaction term for examined tested individual risk factors. A positive interaction term indicates stronger increase in GrimAge2 acceleration and pace of aging whereas a negative term indicates less increase. Filled in circles represent nominally significant interaction terms. The asterix marks interaction terms that remain significant after multiple testing correction. For a complete overview of all exploratory analysis results, please see *SI Appendix*, sections 4.4 and 4.5 and Fig. S11. SES = socioeconomic status, PGS = polygenic score, BMI = body-mass-index.

In our main analysis, we tested for the association between pandemic-related stressors and change in epigenetic aging by fitting a time x stressor interaction term for each stressor as predictor and the two repeated measures of each clock as outcome in a linear mixed regression (*Materials and Methods*). Multiple testing was corrected for by multiplying the *P*-value by the effective number of independent tests (n = 9). Associations between pandemic-related stress and changes in epigenetic aging were only significant in adolescents (Fig. 3*A*). There were no significant associations in children or adults (*SI Appendix*, section 4.3). Specifically, adolescents with a higher perceived burden during the third lockdown presented with higher increase in DunedinPACE (*β* = 0.04, *p.adj* = 0.01) from prepandemic baseline to pandemic follow-up. The direction of this association was consistent with GrimAge2 acceleration (*β* = 0.02, *p.adj* = 0.09), as well as associations with perceived burden during the earlier second lockdown (DunedinPACE: *β* = 0.01, *p.adj* = 0.36; GrimAge2: *β* = 0.02, *p.adj* = 0.05). In addition, adolescents living in regions with higher 7-d incidence rate showed less increase in PedBE age acceleration (*β* = −0.05, *p.adj* = 0.002). Both interaction effects attenuated but remained significant after controlling for BMI and smoking, with alternative batch correction (correcting for row and plate using Combat, see *Materials and Methods*); when excluding ever infected households, and when accounting for regional clustering for the 7-d incidence rate (*SI Appendix*, section 4.3). Focusing on the effects of perceived pandemic burden on epigenetic aging, exploratory analyses of single burden items revealed mostly significant effects for items indicative of social interactions (i.e., more separation from important people and more restriction of civil rights associating with higher pace of aging, *SI Appendix*, Table S10).

### Epigenetic Change in Relation to Individual Risk Factors.

Next, we explored whether change in epigenetic aging was associated with individual differences that may contribute to more severe stress experiences and that have been previously linked to poorer physical health and more severe anxiety and depressive symptoms during the COVID-19 pandemic (18, 23–25). We fit linear mixed models with a time x predictor interaction term and the repeated measures of each clock as outcome. Multiple testing was corrected for by multiplying the *P*-value by the effective number of independent tests (n = 11). Of all 13 demographic, mental and physical health, and home environmental predictors, physical health variables including BMI and DNAm-derived scores for BMI (45) and smoking (46) (Table 2) had the strongest association with change in epigenetic aging over time across all age groups and clocks (Fig. 3*B*). For the biological clocks, individuals with higher baseline BMI (DunedinPACE: *β* = 0.17, *p.adj* < 0.0001; GrimAge2: *β* = 0.06, *p.adj* < 0.0001), higher DNAm-based BMI (DunedinPACE: *β* = 0.31, *p.adj* < 0.0001; GrimAge2: *β* = 0.16, *p.adj* = 0.0001), and higher DNAm-based smoking (DunedinPACE: *β* = 0.27, *p.adj* < 0.0001; GrimAge2: *β* = 0.20, *p.adj* < 0.0001) showed a stronger increase in epigenetic age acceleration and pace of aging (*SI Appendix,* Tables S12 and S13) during the pandemic. Effects were less consistent for the two chronological clocks (*SI Appendix*, Fig. S11 and Tables S12 and S13). In addition, individuals with a lower parental SES and lower well-being PGS presented with a higher increase in DunedinPACE (SES: *β* = −0.03, *p.adj* = 0.004; well-being PGS: *β* = 0.02, *p.adj* = 0.02). Moreover, adolescents living in larger cities presented with a lower increase in epigenetic age acceleration (GrimAge2: city of 20,000 to 99,999: *β* = −0.08, *p.adj* = 0.02; city of >500,000: *β* = −0.09, *p.adj* = 0.007) and PedBE:city of > 500,000: *β* = −0.15, *p.adj* = 0.0006). This association was not observed in other age groups. The direction of associations was generally consistent when additionally exploring associations of the different risk factors with epigenetic clocks at prepandemic baseline and pandemic follow-up separately (*SI Appendix*, section 4.4 and Fig. S11).

Because we did not observe significant associations of the objective indicators of pandemic-related stress with change in the biological clocks, we explored the relationship between subjective pandemic perception, objective indicators of pandemic stress and individual risk factors. Perceived pandemic threat and burden, as well as anxiety during the pandemic were significantly associated with a number of baseline risk factors (*SI Appendix*, section 4.5 and Fig. S10). Specifically, individuals of female sex, lower parental SES, more chaotic home environment, living in a smaller city, a lower polygenic score for well-being (well-being PGS) (47), and higher prepandemic level of depression and anxiety presented with more perceived threat and higher anxiety during the pandemic (*SI Appendix*, Fig. S11 and Table S15). Yet again, few associations of pandemic perception with objective measures were found and none remained significant after multiple testing correction.

## Discussion

We identified a significant acceleration in biological aging based on PedBE, GrimAge2, and DunedinPACE over the course of the COVID-19 pandemic in children, adolescents, and adults. In adolescents, specific subjective and objective measures of pandemic stress were associated with this change while this was not observed in other age groups. Specifically, higher perceived pandemic burden was significantly associated with increased pace of aging in this age group. This was related to the separation from important people and restriction of civil rights, and suggests that mid to late adolescence may be a sensitive period for subjective stress-related alterations in biological aging. Overall, however, factors related to physical health were the variables that were most strongly related to epigenetic age acceleration in all age groups, suggesting that a complex interplay of changes in both psychosocial and physical factors contributes to the observed age acceleration. Interestingly, objective measures of pandemic stress not only showed inconsistent association with epigenetic age acceleration but were also not consistently associated with perceived pandemic threat and burden. The latter were mainly associated with prepandemic factors, including depression and anxiety, parental SES, chaos in the home environment, city size, and the well-being PGS, supporting the presence of potentially more vulnerable groups of individuals in the population consistent with prior studies (18, 23–25).

### Epigenetic Aging Accelerated During the COVID-19 Pandemic.

In this study, we observed significant increases in GrimAge2 and PedBE epigenetic age acceleration and DunedinPACE of aging from prepandemic baseline to pandemic follow-up. Prior longitudinal studies (total N = 2,473) of salivary DNAm in healthy adolescents (ages 9 to 15) over 4 y (48) and young adults (ages 17 to 25) over 8 y (49) report no systematic increases in Horvath DNAmAge acceleration, GrimAge2 acceleration, or DunedinPACE in the absence of severe stressors. In contrast, studies of stress-exposed groups including those reporting stressful life events (50) or structural stressors such as ethnic disparities (48, 51) and paramedic training (52) have documented increased epigenetic age acceleration and pace of aging based on saliva-derived Horvath DNAmAge, GrimAge2, and DunedinPACE (ages 8 to 43). This pattern suggests that chronic or pronounced stress exposure is associated with accelerated epigenetic aging trajectories, whereas normative development in healthy populations typically does not. However, direct quantitative comparison between TECS and previously published studies is limited by differences in statistical modeling, age ranges, and follow-up durations, and from this observation alone we cannot discern whether the observed changes originate from the pandemic stressors, other societal changes during this time period, or simply changes in epigenetic aging with time and development. Future studies may facilitate such conclusions by establishing normative developmental trajectories for epigenetic aging and pace-of-aging measures.

The increase in epigenetic aging in our study was not consistent across all clocks in all age ranges, possibly due to the differences in the training procedures between epigenetic clocks. In detail, our sample assessed epigenetic clocks from DNAm from saliva and across a young age range, including childhood. GrimAge2 and DunedinPACE were trained on adult blood tissue (39, 42) and the Horvath clock with an age range from 1 to 101 y, while the PedBE clock was trained on pediatric buccal tissue in youths of less than 20 y of age (41). The PedBE clock may thus be the only clock with sufficient sensitivity to track small effects in saliva samples of children as observed in our study. From adolescence onward, the adult-trained biological clocks perform better (*SI Appendix*, section 2.9), which could explain why we observe more consistent acceleration across clocks in older individuals (39, 42). The absence of change in Horvath DNAmAge acceleration in our data may reflect the lower sensitivity of chronological clocks to environmental and psychosocial stressors, as compared to more biologically informed measures like GrimAge2 and DunedinPACE (37, 53). This aligns with recent evidence from a cross-tissue study in a pediatric sample showing consistent association of lower SES with higher GrimAge2 age acceleration and Dunedin pace of aging in n = 291 matched blood and saliva samples, whereas no such significant associations were observed for Horvath DNAmAge or PedBE (44).

### Adolescence as Sensitive Period for the Psychosocial Burden of the Pandemic.

Associations of both subjective and objective pandemic stressors with biological aging were only statistically significant among adolescents, but not in children, adults, or the full sample. Mid to late puberty may thus represent a sensitive period for cumulative, stress-related alterations in biological aging. This sensitivity may arise from vulnerable biological processes at this age, but also from social and health-related circumstances related to lockdown-specific restrictions that may most affect this age group. This includes reduced possibilities to establish peer relationships or to be physically active, which likely impact biological aging (54). In line with this, items of the perceived burden scale referring to reduced social interactions and leisure activities were most strongly associated with change in PedBE age acceleration and DunedinPACE (*SI Appendix,* Table S10).

Yet, associations of these items and the associations with perceived burden and 7-d incidence were in opposite direction, with higher burden linked to increased DunedinPACE and higher 7-d incidence to reduced PedBE age acceleration. This suggests that PedBE and DunedinPACE may capture distinct stress-related biological processes. PedBE is a chronological clock and thus captures how DNAm patterns accumulate over the lifespan. Our finding suggests that this accumulation slows down with higher 7-d incidence rate. However, the specific processes behind this association remain speculative. PedBE has only been validated in relation to gestational age and birth weight (41), while previous associations with psychosocial adversity are mixed (55, 56) and absent even in buccal cells which correspond to the initial training tissue (57). Likewise, the interpretation of 7-d incidence rate is challenging because it is the result of a complex interplay of governmental measures, adherence, and testing facilities among many other factors. In contrast, DunedinPACE has been consistently associated with SES and childhood adversity in both blood and saliva and in both youth and adults (8, 44, 58). It was directly trained on longitudinal changes in 19 biomarkers assessing cardiovascular, metabolic, renal, hepatic, immune, dental, and pulmonary systems (39), and can thus be more informative of disease risk than chronological clocks like PedBE (5). This is further supported by a positive association of DunedinPACE, but not PedBE, with BMI in our sample. Overall, our finding of increased pace of aging during the pandemic aligns with a broader literature indicating that inflammatory and metabolic processes, that DunedinPACE was trained on, can be affected by psychosocial adversity (59).

### Multifaceted Influences on Epigenetic Change and Pandemic Perception.

Across age groups, GrimAge2 acceleration and DunedinPACE were consistently associated with indicators of physical health and health-related behaviors (i.e., BMI, smoking exposure). This pattern is consistent with prior studies (7) and supports the utility of biological clocks as markers of disease risk (5). Accordingly, observed increases in sedentary lifestyle and weight gain during the pandemic (19–22) may also partly account for the observed increases in GrimAge2 and DunedinPACE in the overall sample, alongside psychosocial burden in adolescents.

Our findings revealed that lockdown-related restrictions mostly did not directly relate to increased anxiety, or perceived threat or burden. In fact, subjective stress and anxiety during the COVID-19 pandemic were more strongly associated with prepandemic differences in SES and mental health, as well as the well-being PGS, female sex, and city size rather than objective measures such as 7-d incidence rate or policy stringency. Several of these factors were also associated with epigenetic age acceleration. These included parental SES, city size, and the well-being PGS (*SI Appendix,* Fig. S11). While psychosocial stress was only directly related to epigenetic age acceleration in adolescents, these factors might have indirectly contributed to epigenetic change in the other age groups. Overall, our findings thus suggest a multifaceted relationship of factors related to mental health status, home environment, and PRS-derived genetic liability that influence the subjective perception of the pandemic and epigenetic aging across the pandemic.

### Limitations.

Our study comprised a young, healthy, population-based sample, which limits insights into aging trajectories under more adverse circumstances and may underestimate the biological impact of pandemic-related stressors. Also, the policy stringency used to quantify government-imposed containment measures encompassed an average across 21 areas and, while measures across areas are interconnected, tailoring the policy stringency to specific age groups (e.g., travel restrictions for adults, school closures for children and adolescents) may have been more suitable. In addition, our study relied on correlational analyses and does not capture causal mechanisms. For example, we cannot fully exclude viral infection itself (and increased viral exposure in certain contexts) as a contributing factor, though associations with pandemic stressors remain significant after excluding all ever-infected households. Likewise, associations with the well-being PGS may reflect environmental confounding in addition to genetic influences. While we tried to select clocks with stress sensitivity in saliva in the age groups of TECS, accuracy is likely still limited and this could be improved with future clocks trained on biological processes in the matching age ranges and tissue (37). In addition, while biological processes across development can be nonlinear (60), our statistical analyses of within-individual change were limited to linear comparisons as we had only two repeated measures within individuals.

### Conclusion.

We observed significant acceleration in biological aging during the COVID-19 pandemic in children, adolescents, and adults. We identified age-specific contributors, such as social isolation in adolescents, and an overall complex interplay of largely preexisting social, psychological, and physical health factors that may underlie this acceleration. Long-term follow-up will be needed to confirm whether these changes persist and predict adverse health outcomes later in life.

## Materials and Methods

Please see more detailed descriptions in *SI Appendix*.

### Study Design.

We used data from the TECS project of the German TwinLife study (36). TwinLife is a longitudinal, cross-sequential study with an extended twin family design, including annual survey data from monozygotic (MZ) and dizygotic (DZ) same-sex twin pairs and their families across Germany. The twin sample comprises four age groups with twins born in 1990–1993, 1997/1998, 2003/2004, and 2009/2010. From December 2018 to April 2020, a first saliva sample was collected from TwinLife participants for genotyping, as part of the TwinSNPs satellite project. A second saliva sample was collected from TwinLife participants between September 2021 and May 2022. The timing of the saliva collection, along with periods of COVID-19 containment measures in Germany, is illustrated in *SI Appendix*, Fig. S9.

For this study, we included TwinLife participants with valid DNAm data available from both collections of saliva and for whom complete information on subjective pandemic-related stressors was available (35). This resulted in a sample size of *n* = 1,028 twins, after quality control, data preprocessing and exclusion of participants with their first saliva sample collected after the start of the COVID-19 pandemic in Germany, in March 2020. Our final analytic sample included 359 children, 385 adolescents, 157 young adults, and 127 adults. For our age stratified analyses, we combined the two latter age groups (hereafter referred to as adults, *n* = 284) to achieve sample sizes comparable to our child and adolescent age group (*SI Appendix*, sections 2.1 and 2.3).

### Genotyping and DNA Methylation Assessment.

Saliva samples were collected using the Oragene® saliva self-collection kits (OG-500 and OG-600; DNA Genotek, Canada), genotyped using the Illumina Global Screening Arrays (GSA+MD-24v3.0-Psych-24v1.1, Illumina, San Diego, CA), and DNAm assessed using the Infinium MethylationEPIC BeadChip v1.0 (Illumina, San Diego, CA, USA). DNAm of both timepoints per participant was run on the same array and preprocessed together (*SI Appendix*, sections 2.2 and 2.3).

### Biological Aging.

We selected two chronological and two biological clocks (Table 2) that have been associated with mortality and/or psychosocial stressors in the literature and have previously been examined in young samples: Horvath (40), PedBE (41), GrimAge2 (42), and DunedinPACE (39) (*SI Appendix*, sections 2.4 and 2.5 and Figs. S2–S6 for clock performance based on chronological age).

### Pandemic Stressors.

We defined two objective and two subjective indicators of pandemic stress: 7-d incidence rate and the policy stringency index of government-imposed containment measures, as well as self-reported pandemic threat and burden (Table 2). The course of the COVID-19 pandemic in Germany is explained in detail in *SI Appendix*, section 2.6.

We matched public records of 7-d incidence rate and policy stringency to the regional district of our participant’s place of residence during the pandemic. For each participant, we thereby tracked the local trajectory of 7-d incidence rate and policy stringency and estimated exposure to these stressors during the first, second, and third lockdown using the area under the curve (AUC). In contrast to mean or sum scores, the AUC considers the temporal duration of the given exposure (61). Since effects may differ depending on the timing of the exposure, we computed the AUC for the first lockdown (March 1st to 11th of May 2020), second lockdown (November 2nd 2020 to June 1st 2021), cumulative (starting from the 1st of March 2020) and recent (starting 30 d prior to the saliva collection) exposure up to the date of the second saliva collection (*SI Appendix*, section 2.7 and Figs. S7 and S9).

Perceived pandemic threat and burden were based on self-reports during the second and third lockdown. Participants rated how threatened they felt by the pandemic across various areas of life, such as their health, social relationships, school performance, and economic situation (62). Pandemic burden was rated in terms of social isolation, lack of leisure activities, irregular daily routine, home schooling, and restriction of civil rights, among others (63). The number (between 4 to 7) and type of items varied slightly depending on age group, scale, and lockdown. To summarize pandemic threat and burden, we computed average scores across all items of the respective scale for each participant during the second and third lockdown (*SI Appendix*, section 2.8).

### Covariates, Demographic Background, and Risk Factors.

Table 2 provides an overview of all outcomes and predictors examined in this study. These included demographic background (genetic sex, SES), the home environment (self-reported quality home chaos, city size, for adults: whether they left the parental home), the polygenic score for subjective well-being (well-being PGS), and mental (anxiety and depressive symptoms) and physical (prepandemic BMI, epigenetic BMI, epigenetic smoking) health. The well-being PGS consists of genetic variants linked to a spectrum of well-being measures including life satisfaction, positive affect, neuroticism, and depressive symptoms. We previously identified it as the most predictive PGS for mental health burden during the COVID-19 pandemic in a nonclinical sample (23) (*SI Appendix*, sections 2.9 and 2.10).

### Statistical Analysis.

A detailed description of the statistical methods, including specific R-packages and model equations of each analysis step are provided in *SI Appendix*, section 3. All dependent and independent variables were standardized before analysis.

The statistical analyses were conducted in R (version 4.1.3) using a linear mixed model approach to account for repeated measures and family structure (64). In this approach, we use a random intercept for each individual, accounting for correlation among their repeated outcomes. The correlation due to genetic relatedness between individuals is accounted for by using a genetic relatedness matrix (GRM). The entries in this matrix are kinship coefficients that reflect genetic relatedness between individuals (i.e., varying around 1 for MZ twins, around 0.5 for DZ twins, and around 0 for individuals from different families). They specify the covariance of random intercepts per individual with random intercepts of other individuals (i.e., genetically related vs. unrelated). Associations of independent variables with the repeated outcome were tested in the fixed effects component of the model. *P*-values for the fixed effects are based on a Wald test. Statistical significance was tested at α < 0.05. Multiple testing was corrected for by multiplying the *P*-value by the effective number of independent tests (M_eff_). All analyses were run for the full sample and stratified by age group to explore developmental phase-specific associations. Epigenetic age acceleration (and pace of aging) was modeled following the guidelines by Krieger et al. (65) with the raw epigenetic clock estimates at prepandemic baseline and pandemic follow-up as repeated outcomes, controlling for chronological age and cell type in the fixed effects of the regression model.

To test whether biological age changed during the COVID-19 pandemic, we used epigenetic age as the dependent variable and time alongside chronological age, cell type, and sex as fixed effects (Model 1/M1). Time was coded as continuous variable in years, ranging from prepandemic baseline = 0 to pandemic follow-up = between 1.5 to 3 y, because the prepandemic date of the saliva collection differed between individuals (December 2018 to March 2020). A positive coefficient for time in this model indicates an increase and a negative coefficient a decrease in age acceleration or pace of aging from prepandemic baseline to pandemic follow-up, respectively. We repeated this analysis including epigenetic smoking and BMI as additional covariates (Model 2/M2) to examine whether the observed change remained significant. We applied this two-step approach (M1, M2) because BMI and smoking are known to affect epigenetic age (37, 66) and can be considered confounding factors. Yet, they also represent health behaviors of interest that may underlie the relationship between pandemic stressors and epigenetic aging (66). We then investigated whether the within-individual change in epigenetic age acceleration and pace of aging was dependent on pandemic stressors by adding a time x stressor interaction term. For this analysis, we also fitted M1 and M2. To exclude viral infection as the main driver of potentially significant findings, we excluded (self-reported) ever-infected households in a sensitivity analysis of significant findings. Additional robustness checks of significant findings included controlling for regional clustering and applying alternative DNAm batch correction without chip adjustment (samples were chip-matched).

For the significant association with perceived burden, we reran the analyses with the questionnaire items to identify which aspect of pandemic burden had driven the association. Moreover, we explored the effects of risk factors on epigenetic aging by examining main effects of each risk factor on epigenetic age at prepandemic baseline and pandemic follow-up as dependent variable, as well as the time x risk interaction term with repeated measures of epigenetic aging as outcome. In addition, we examined the relationship of factors and objective stressors with subjective stressors and mental health by testing for main effects on each self-report measure before and during the second and third lockdown, controlling for age and sex.

## Ethics Statements

The TwinLife study was approved by the ethics committee of the German Psychological Society (protocol number: RR 11.2009). The Ethics Committee of the University of Bielefeld (Application No. 2020-106) additionally approved the extension of TwinLife to include the supplementary COVID-19 surveys. The Ethics Committee of the Medical Faculty of the University of Bonn (No. 113/18) approved the study protocols for the collection and the analysis of the (epi)genetic data based on the saliva samples. All participants were informed in writing about the aims of the TwinLife study, the incentives, and that their participation was voluntary and could be withdrawn at any time. In addition, in all face-to-face surveys, participants received a two-page leaflet summarizing the procedures concerning data protection and privacy and their rights to demand the deletion of all data collected in line with German data integrity laws. Written material was sent to participants before all interviews. At the beginning of each interview, informed verbal consent was obtained from participants (and the participants’ legal guardians when the participants were underage) and documented. Prior to the collection of genetic data, the participants received extensive written information on, e.g., the procedures of saliva collection, the scope of the study, their right to refuse participation, and their right to withdraw their consent at any given time. For collection of genetic data, written informed consent was obtained from all participants and the participant’s legal guardian (for minors below age 18).

### Declaration of Generative AI and AI-Assisted Technologies in the Writing Process.

During the preparation of this work the author(s) used ChatGPT-4 in order to refine the scientific writing (grammar, smooth transitions, structure of paragraphs) of the introduction and discussion. After using this tool/service, the author(s) reviewed and edited the content as needed and take(s) full responsibility for the content of the publication.

## Supplementary Material

Appendix 01 (PDF)

## Data Availability

In accordance with data protection regulations and the participants’ consent forms, access to the data, including (epi)genetic and geolocalized data, is provided through the TwinLife Data Center at Bielefeld University. After completing a data request form via https://www.twin-life.de/documentation/10-molepigen (67), researchers will be granted access to the data as soon as they have provided a written commitment to comply with data protection regulations. The data access is provided to researchers free of charge. The analysis code is publicly available at OSF analysis_code_osf.io (68).
