# Supplementary material for: Epigenetic aging during the COVID-19 pandemic: A prospective longitudinal study with prepandemic baseline data in children, adolescents, and adults
Source: Proc Natl Acad Sci U S A. 2026 Nov 3;123(45):e2604211123. doi: 10.1073/pnas.2604211123 (PMC13752343; doi:10.1073/pnas.2604211123)
Supplement: Supplementary file 1 [file pnas.2604211123.sapp.pdf]

PNAS

Supporting Information for

Epigenetic aging during the COVID-19 pandemic: A prospective longitudinal study with pre-pandemic baseline data in children, adolescents, and adults

Alicia M. Schowe*[1,2], Mirko Ruks[3,4], Charlotte K.L. Disselkamp[5], Dmitry V. Kuznetsov[3,6], Jana Instinske[4,7], Theresa Rohm[7], Andreas J. Forstner[5,8], Markus M. Nöthen[5], Bastian Mönkediek[3,4,9], Christian Kandler[4,7,9], Darina Czamara*[#1], Elisabeth B. Binder*[#1]

[1]Dept. Genes and Environment, Max-Planck-Institute of Psychiatry, 80804 Munich, Germany
[2]Graduate School of Systemic Neuroscience, Ludwig-Maximilians Universität, 82152 Planegg, Germany
[3]Faculty of Sociology, Bielefeld University, 33615 Bielefeld, Germany
[4]Dept. of Psychology, Bielefeld University, 33615 Bielefeld, Germany
[5]Institute of Human Genetics, University of Bonn, School of Medicine & University Hospital Bonn, 53127 Bonn, Germany
[6]Center for Environmental Neuroscience, Max Planck Institute for Human Development, 14195, Berlin, Germany
[7]Dept. of Psychology, University of Bremen, 28359 Bremen, Germany
[8]Institute of Neuroscience and Medicine (INM-1), Research Center Jülich, 52428 Jülich, Germany
[9]Joint Institute for Individualisation in a Changing Environment (JICE), University of Münster & Bielefeld University, Münster/Bielefeld, Germany

[#]Shared last authorship

*Corresponding authors: Alicia M. Schowe, Darina Czamara, Elisabeth B. Binder.
Email: alicia_schowe@psych.mpg.de, darina@pszch.mpg.de, binder@psych.mpg.de

This PDF file includes:

Supporting text
Figures S1 to S11
Tables S1 to S16
SI References

Table of content

1. STUDY DESIGN 3

Figure S1. TwinLife Study Design. 4

2. PROCEDURES 5

2.1 SALIVA COLLECTION 5

2.2 GENOTYPING, GENETIC RELATEDNESS, AND THE POLYGENIC SCORE FOR WELL-BEING..........5

2.3 DNA METHYLATION 5

2.4 BIOLOGICAL AGING: EPIGENETIC CLOCK SELECTION AND COMPUTATION..............................6

Table S1: Background of epigenetic clocks examined in our analytic sample.....................7

2.5 BIOLOGICAL AGING: EPIGENETIC CLOCK PERFORMANCE 8

Figure S2. Median absolute error by age group. 9

Figure S3. Mean deviation by age group. 10

Figure S4. Association between epigenetic and chronological age. 11

Figure S5. Epigenetic age acceleration and pace of aging by age group. 12

Figure S6. Associations between epigenetic clocks at pre-pandemic baseline and follow-up. 13

2.6 PANDEMIC STRESSORS: THE COURSE OF COVID-19 IN GERMANY 14

Figure S7. Median 7-Day incidence of confirmed COVID-19 cases in Germany and TECS 15

Figure S8. Median COVID-19 policy stringency in Germany and for the TECS sample. .16

2.7 FIGURE S9. TIMING OF INVESTIGATED PANDEMIC STRESSORS IN RELATION TO THE TWINLIFE STUDY DESIGN..17

2.8 PANDEMIC STRESSORS: MEASUREMENT OF 7-DAY INCIDENCE RATE AND POLICY STRINGENCY 18

2.9 PANDEMIC STRESSORS: SELF-REPORTED PANDEMIC THREAT AND BURDEN 18

2.10 COVARIATES. 18

2.11 DEMOGRAPHIC BACKGROUND AND RISK FACTORS 19

Genetic sex. 19

BMI 19

Socioeconomic status. 19

Home environment. 19

Anxiety symptoms. 19

Depressive symptoms....20
2.12 SURVEY ITEMS....20
Table S2. Individual survey items....21
3. STATISTICAL ANALYSIS....26
3.1 R-PACKAGES....26
Table S3. R-packages....26
3.2 EFFECTIVE NUMBER OF INDEPENDENT TESTS....26
3.3 MODEL EQUATIONS....26
Figure S10. Simplified DAG of hypothesized relationships....27
Change in epigenetic aging....28
Pandemic stressors and change in epigenetic aging....28
Individual risk factor, pandemic stressors, and epigenetic age before and during the pandemic....29
Individual risk factors, and objective pandemic stressor effects on anxiety pandemic threat and burden....29
4. RESULTS....30
4.1 DESCRIPTIVE STATISTICS....30
Table S4. Descriptive statistics of COVID-19 pandemic subjective and objective pandemic stressors in the TECS sample....30
Table S5. Descriptive statistics of perceived home chaos and mental well-being....32
Table S6: Descriptive statistics of raw and residualized epigenetic age based on pre-pandemic and pandemic saliva sampling....33
4.2 BIOLOGICAL AGING DURING THE COVID-19 PANDEMIC....34
Table S7: Change in epigenetic aging during the COVID-19 pandemic....35
Table S8: Change in epigenetic aging during the COVID-19 pandemic after excluding ever-infected households....36
4.3 PANDEMIC STRESSORS AND CHANGE IN BIOLOGICAL AGING....37
Table S9. Effects of pandemic stressors on within-individual change in epigenetic aging....38
Table S10. Associations between single burden items and change in epigenetic aging in adolescents....41
4.4 ASSOCIATIONS OF RISK FACTORS AND EPIGENETIC AGING....42
Table S11. Associations between risk factors and pre-pandemic epigenetic aging....42

Table S12. Associations between risk factors, pandemic stressors, and epigenetic aging at pandemic follow-up. 44
Table S13. Associations between risk factors and change in epigenetic aging from pre-pandemic baseline to pandemic follow-up. 46
4.5 ASSOCIATIONS OF RISK FACTORS AND OBJECTIVE STRESSORS WITH SUBJECTIVE WELL-BEING AND STRESSORS 48
Table S14. Associations of risk factors with pre-pandemic anxiety and depression at pre-pandemic baseline 49
Table S15. Associations of risk factors and objective stressors with anxiety and subjective stressors during the second lockdown. 50
Table S16. Associations of risk factors and objective stressors with anxiety and subjective stressors during the third lockdown. 52
4.6 FIGURE S11. HEATMAP OF ALL ASSOCIATIONS OF OBJECTIVE PANDEMIC STRESSORS AND INDIVIDUAL RISK FACTORS WITH EPIGENETIC AGING AND PANDEMIC PERCEPTION 54
5. REFERENCES 56

1. Study Design

A detailed description of the TwinLife study is provided Mönkediek et al.(1) and Frach et al.(2). The twin sample comprises four birth cohorts with twins born in 2009/2010 (cohort 1), 2003/2004 (cohort 2), 1997/1998 (cohort 3) and 1990-1993 (cohort 4). Each of the four birth cohorts includes two birth years (subsample A and B), interviewed in consecutive years (see Figure S1). The first data collection took place in form of a household interview (face-to-face 1= F2F 1) from October 2014 to April 2016, where the twins were 5, 11, 17, and 23 years of age, respectively, followed by a computer-assisted telephone interview (CATI 1) from November 2015 to April 2017. The time interval between two data collections is always one year, and participants are interviewed alternately at home and by telephone (i.e., F2F 2 in 2016–2018, CATI 2 in 2017–2019, F2F 3 in 2018–2020, CATI 3 in 2019–2021, and F2F 4 in 2020–2022). Moreover, three COVID-19 supplementary surveys were conducted. The first supplemental COVID-19 survey (Cov1) retrospectively assessed behaviour, attitudes, health, stressors and socio-economic changes during the first phase of strict government-imposed containment measures from March 2020 to June 2020 and was conducted from July to November 2020. The second supplemental COVID-19 survey (Cov2) was part of the F2F 4 and CATI 3 data collection. The third supplemental COVID-19 survey (Cov3) did not overlap with a regular data collection of the TwinLife study and was sent to the TwinLife participants by post, along with saliva self-collection kits for the second saliva collection, from September to December 2021. Subsequent surveys are not considered in the present study, and subsequent saliva samples are, to date, not yet processed for genotyping or DNA methylation assessment (see Figure S1).

Figure S1. TwinLife Study Design.

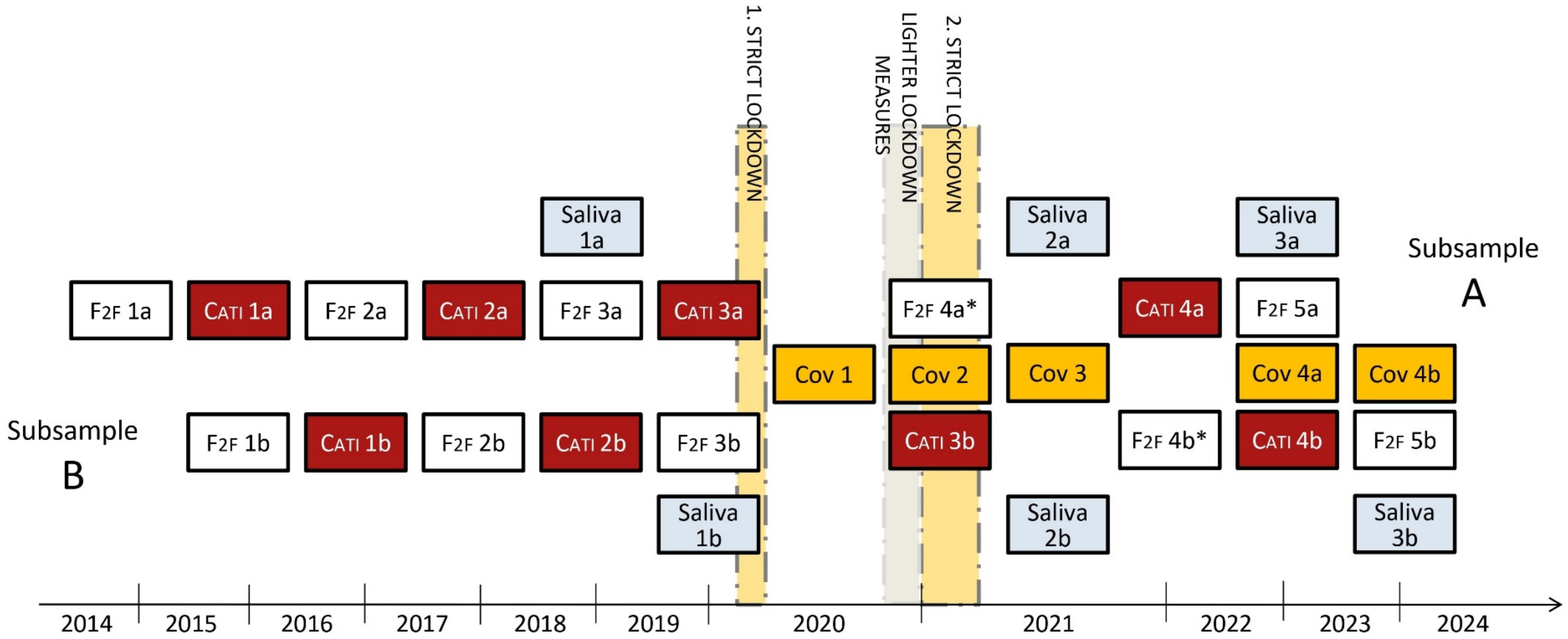

Note: Illustration of the repeated survey and saliva collection occasions in the TwinLife sample. Each of the four age cohorts (i.e., the same-sex twin pairs of 5, 11, 17, and 23 years of age at the time of the first survey) includes two birth years (subsample A and B) who are interviewed in consecutive years. To date, only the first two saliva samples have been used for genotyping and DNA methylation assessment. F2F = face-to-face interview, CATI = computer-assisted telephone interview, saliva = collection of saliva via self-collection kit, Cov = COVID-19 supplementary surveys. *The fourth face-to-face interview in the household had to be substituted with a CATI & CAWI (computer assisted web interview) due to the COVID-19 pandemic. In the fifth face-to-face interview, participants could choose between a conventional household visit and a CATI & CAWI variant of the survey; 1. strict lockdown and 2. strict lockdown: contact restrictions in private life, travel restrictions, closure of schools, gastronomy, retail, service sector, cultural institutions (e.g., museums, cinema, concerts) and social sports activities; lighter lockdown measures: contact restrictions in private life and closure of gastronomy, cultural institutions and social sports activities. Reprinted with permission from Rohm et al. (3).

2. Procedures

2.1 Saliva collection

Two saliva samples were collected from TwinLife participants (including twins and their extended family members) using Oragene® saliva self-collection kits (OG-500 and OG-600; DNA Genotek, Canada). The first saliva sample was collected during the regular F2F 3 interviews from December 2018 to April 2020. During the household visit, the interviewers accompanied the procedure of saliva collection, and a short video explained the study goals and procedures of the saliva collection. For the second saliva collection, TwinLife participants were contacted via post. Letters included the consent forms, the saliva self-collection kits, and detailed instructions for saliva self-collection. This second saliva collection took place from September 2021 to May 2022. DNA extraction, genotyping and further genomic data processing was performed at the Institute of Human Genetics and the Institute for Genomic Statistics and Bioinformatics of the University of Bonn and the University Hospital Bonn, Germany.

2.2 Genotyping, genetic relatedness, and the polygenic score for well-being

DNA was extracted according to standard procedures. Genotyping was performed using Illumina Global Screening Arrays (GSA+MD-24v3.0-Psych-24v1.1, Illumina, San Diego, CA, USA), customized to include additional markers relevant to psychiatric disorders, according to the manufacturer's protocol. Quality control and preprocessing of genotype data were performed as described in Deppe et al.(4). In total, raw genotype data was generated for 5,927 TwinLife samples. At the sample level, individuals were excluded on the basis of a low overall genotyping rate, deviating autosomal heterozygosity, a mismatch between genetically inferred and self-reported sex, discordance between genetically inferred and reported family structure (relatedness), and population substructure (retaining only individuals of European ancestry and excluding principal-component outliers). After these steps, 5,421 samples remained. The analytic sample for the current study consists of a subset of these genotyped individuals, selected for DNA methylation assessments as part of the TECS project using the following criteria: the individual is an MZ or DZ twin with a salivary sample available at both timepoints, with valid consent for saliva collection and participation at both the second and third TwinLife supplemental COVID-19 survey (Cov2 and Cov3). Furthermore, both saliva samples had > 1,000 ng DNA, showed no discoloration and had a 260/280 ratio of < 1.6. Considering these quality indicators, complete twin pairs were prioritized for DNA methylation assessment (n = 498 twin pairs). Remaining array slots were filled with individual twin samples (n = 68 twins, see DNA methylation and epigenetic aging).

The polygenic score (PGS) for the well-being spectrum (WBS PGS) was generated using PRS-CS(5) and PLINK(6) as previously described.(4, 7) Briefly, PRS-CS was run in fully Bayesian automated mode (PRS-CS-auto): the global shrinkage parameter φ was learned directly from the GWAS summary statistics rather than fixed a priori, with all other parameters left at their defaults and the 1000 Genomes European panel used as the LD reference. Moreover, to control for genetic relatedness in our main analyses, we estimated a genetic relatedness matrix (GRM) from the genome-wide autosomal single nucleotide polymorphisms (SNPs) of our analytic sample using GCTA (version 1.93.3).(8) This GRM was fitted as a random effects matrix in all linear mixed models (see statistical analysis).

2.3 DNA methylation

DNA methylation was measured using the DNA of 2,128 samples (2 samples each in 1,064 twins) extracted according to the Oragene® saliva self-collection kits (OG-500 and OG-600; DNA Genotek, Canada) protocol. Samples were randomized by chronological age, biological sex, zygosity, and complete versus single twin pair across 23 plates (266 arrays) using the R package Omixer (version 1.4.0)(9). The family identifier was used as a blocking variable to ensure that all samples and timepoints of a twin pair ran on the same batch/array. Bisulfite-conversion of 500 ng DNA was performed using the D5033 EZ-96 DNA Methylation-Lightning Kit (Deep-Well; Zymo Research Corp., Irvine, USA) and methylation profiles assessed using the Infinium MethylationEPIC BeadChip v1.0 (Illumina, San Diego, CA, USA).

Preprocessing of all methylation samples (*n*=2,128) was conducted following the steps by Maksimovic et al.(10) (see https://github.molgen.mpg.de/mpip/EPIC_Preprocessing_Pipeline.git). Scan intensity

signals as stored in .idat files were loaded into R and transformed into beta-values within the R package *minfi* (version 1.40.0).(11) Samples with a mean detection *p*-value > 0.05 were excluded (n = 3). Additionally, we excluded samples showing sex mismatches between estimated sex from methylation data and phenotypic sex from TwinLife questionnaire self-assessments (n = 14). Beta values were normalised using stratified quantile normalization(12), followed by BMIQ.(13) Probes on sex chromosomes (n = 16,263), probes containing SNPs (n = 24,038), cross-hybridizing (n = 46,867) and polymorphic probes (n = 297) according to Chen et al.(14) and McCartney et al.(15) and probes with a detection *p*-value of > 0.01 in one or more samples (n = 79,922) were removed. Afterwards beta-values were transformed into M-values, and batch-effects were removed using *Combat*.(16) For this, we computed a principal component analysis (PCA) on the M-values and checked which batch variables were most strongly associated with the first five principal components. The strongest batch effects were iteratively removed (plate, chip, row). Corrected M-values were re-transformed into beta values. Lastly, we applied *MixupMapper*(17) to the genotype and methylation data to check for possible sample mix-ups. Four mix-ups were detected. For these four cases, both samples (baseline and follow-up) were removed (total of N = 8 individual samples). The resulting dataset consisted of 2,102 samples of n = 1,055 participants (of which n = 489 were complete twin pairs, 263 MZ, 226 DZ pairs) and 698,472 probes.

2.4 Biological aging: epigenetic clock selection and computation

Using the normalized batch-corrected beta values, we computed four commonly used measures of epigenetic aging: Horvath(18), PedBE(19), GrimAge2 (an improved version of the original GrimAge)(20), and DunedinPACE(21) (see Table S1). These four clocks comprise two chronological clocks and two biological clocks that have been previously associated with aging- and stress-related phenotypes and have been applied to young cohorts.(20, 22–31) Notably, PedBE has been specifically trained in children and adolescents which make up most of our sample. Horvath DNAmAge, the PedBE clock, and GrimAge2 represent epigenetic age in years. Positive residuals of epigenetic age regressed on chronological age indicate epigenetic age acceleration (as epigenetic age exceeds chronological age). Conversely, negative residuals of epigenetic age regressed on chronological age indicate epigenetic age deceleration. In contrast to the other three clocks, DunedinPACE was trained on within-individual change trajectories of biomarkers of aging from 23 to 46 years of age.(32, 33) By design, it may thus be specifically sensitive to capture within-individual aging independent of generation-specific influences that may be confounded with clocks trained on cross-sectional data. A DunedinPACE value of 1 represents aging at the same rate as chronological aging and values larger or smaller than 1 represent a faster or smaller rate of aging, respectively.

The Horvath and PedBE clocks were calculated using the R-package methylclock (version 1.01(34)). Version 2 of GrimAge was computed using code from Lu et al. (2022) provided by A. Lu and S. Horvath. DunedinPACE was calculated with the DunedinPACE R-package (https://github.com/danbelsky/DunedinPACE/.) Detailed background of the training sample and available CpG sites in our sample are presented in Table S1. To check the robustness of our epigenetic clocks regarding our batch correction approach, we additionally computed our four clocks (see section 2.5) based on the normalized batch-corrected beta values without adjusting for chip (as samples were blocked together on the same chip) and re-ran our analyses in a sensitivity analysis of significant findings (see section 4.2 and 4.3).

Table S1: Background of epigenetic clocks examined in our analytic sample

		Training dataset			TECS	
Clock	Reference	Tissue	Age range (years)	Phenotype	Available CpGs/ original clock CpGs	*r* chron. age
Chronological clocks						
Horvath	Horvath(35)	Multiple incl. blood and saliva	1–101	Chronological age	334/353	0.80
PedBE	McEwen et al(19)	Buccal	0-20	Chronological age	94/94	0.77
Biological clocks						
GrimAge2	Lu et al.(20)	Blood	40 - 92	12 biomarkers of aging (10 of GrimAge version 1 plus log C-reactive protein and log hemoglobin A1C)	1,030/1,030	0.80
DunedinPACE	Belsky et al.(32)	Blood	26, 32, 38, and 45	Longitudinal change in 19 biomarkers of aging (e.g., blood pressure, leptin levels)	173/173	0.08

Note. Overview of all the computed and evaluated epigenetic clocks. *r* = Pearson's correlation coefficient.

2.5 Biological aging: epigenetic clock performance
We evaluated the performance of our selected epigenetic clocks in our saliva samples based on correlation with chronological age, and median absolute error (MAE) and median deviation relative to chronological age. In general, for clocks that indicate epigenetic age in years like Horvath, PedBE, and GrimAge2, a high correlation with chronological age as well as low MAE and low median deviation are thought to indicate good performance.(18) Correlation coefficients of all clocks with chronological age are shown in Table S1, MAE and median deviation are shown in Figure S2 and Figure S3.

Horvath performed well in all age groups (children: MAE = 2.9-2.8, delta = 3.1-2.9; adolescents: MAE = 3.3-3.7, delta = 2.2-2.7; adults: MAE = 3.2-3.5, delta = 0.8-1.4) whereas PedBE performed well in children and adolescents but not in adults (children: MAE = 1.1-2.0, delta = 1.0-2.1; adolescents: MAE = 2.3-2.9, delta = -2.2 - -2.9; adults: MAE = 8.2-9.5, delta = -9.9 - -8.7). The latter is consistent with the specific purpose of age prediction in children that PedBE was designed for. In our main analysis, we thus did not investigate PedBE for our adult samples. Although the MAE and median deviation for GrimAge2 are high in our young cohort, this clock was still highly correlated with chronological age indicating good tracking of the aging process in years as noted in previous studies(29) (see also Figure S11). In contrast to chronological and other biological clocks, DunedinPACE is trained on within-individual change trajectories of biomarkers of aging from 23 to 46 years of age.(32, 33) A DunedinPACE value of 1 represents aging at the same rate as chronological aging and values larger or smaller than 1 represent a faster or smaller rate of aging, respectively. As expected from the original study, we found a positive correlation with chronological age in our sample.(32)

Scatterplots of the relationship between chronological age and epigenetic age of our four selected clocks (Horvath, PedBE, GrimAge2, and DunedinPACE) are shown in Figure S4. Except for Horvath and DunedinPACE (r = -0.11 at baseline and r = -0.16 at follow-up), all clocks were positively correlated with each other (from r = 0.24 to r = 0.60) and over time (from r = 0.19 to r = 0.80, see Figure S5A). All correlations were positive after correcting clock estimates for cell types (between clocks: r = 0.06 to r = 0.73; over time: r = 0.46 to r = 0.84, see Figure S5).

Figure S2. Median absolute error by age group.

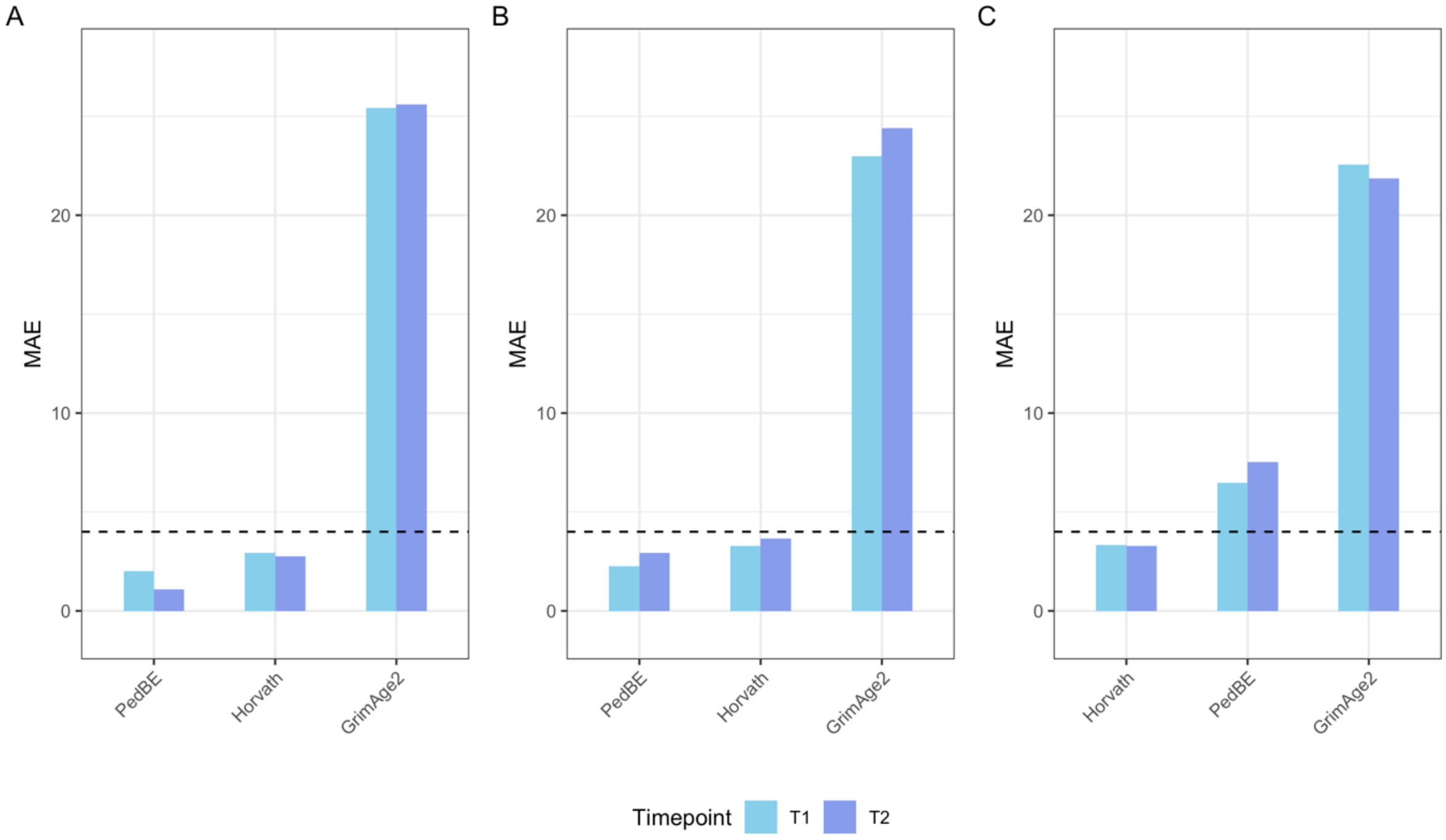

Note. Epigenetic clocks measuring epigenetic age in years sorted in ascending order by median absolute error (MAE; absolute difference between chronological and epigenetic age) for (A) children (chronological age T1: 8.58 to 10.2 years, T2: 10.2 to 12.8 years), (B) adolescents (chronological age T1: 14.8 to 16.2 years, T2: 16.8 to 18.8 years), and (C) adults (chronological age T1: 20.8 to 29.2 years, T2: 22.8 to 31.8 years). Notably, for DunedinPACE, median deviation cannot be computed as this clock measures pace of aging where a value of 1 indicates aging at the same rate as chronological age. The dashed line represents an MAE = 4. An MAE < 4 is thought to indicate good performance for chronological clocks.(35) T1 = first pre-pandemic saliva sample, T2 = second pandemic saliva sample.

Figure S3. Mean deviation by age group.

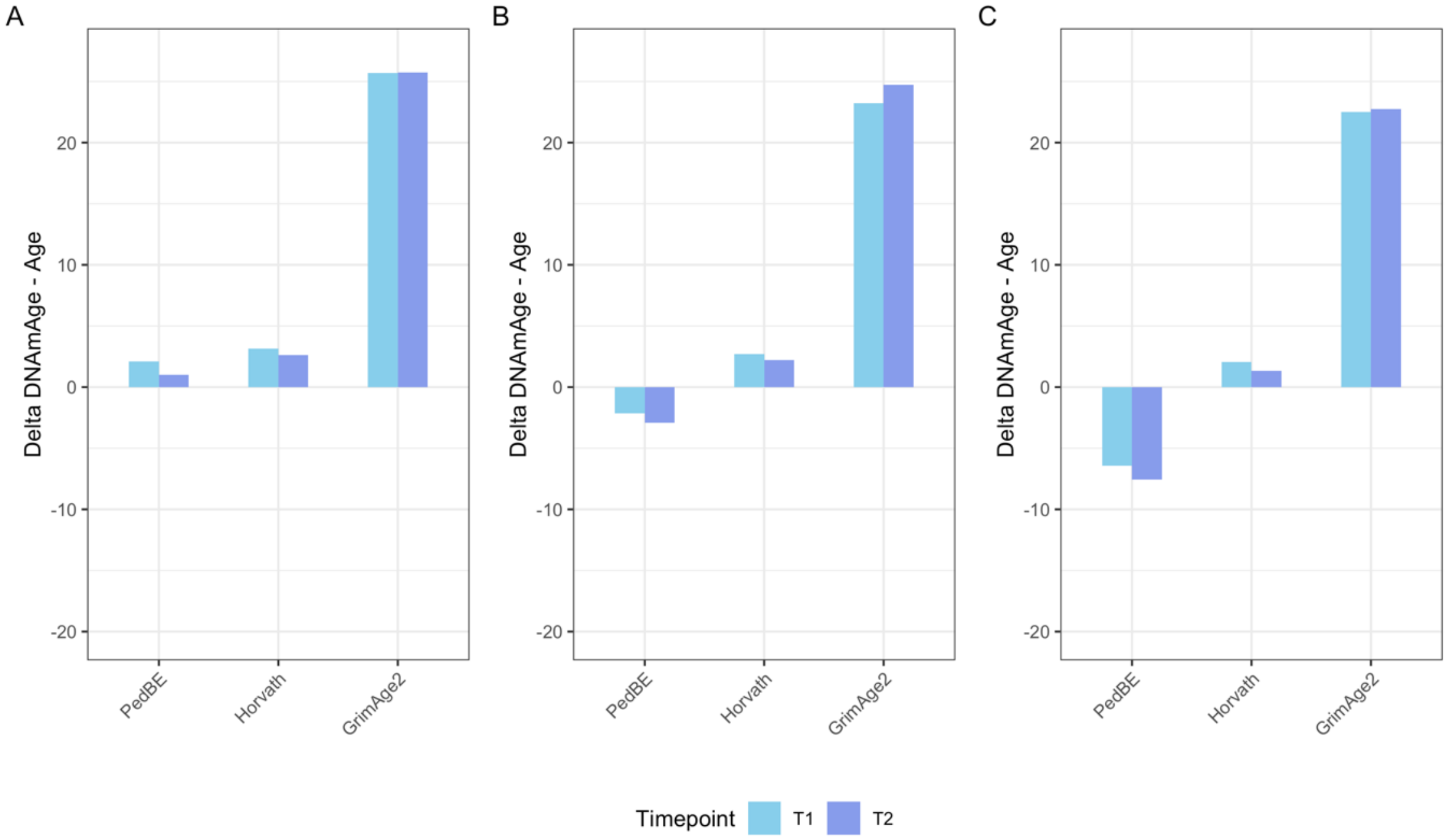

Note. Epigenetic clocks measuring epigenetic age in years sorted in ascending order by median deviation (epigenetic age minus chronological age) for (A) children (chronological age T1: 8.58 to 10.2 years, T2: 10.2 to 12.8 years), (B) adolescents (chronological age T1: 14.8 to 16.2 years, T2: 16.8 to 18.8 years), and (C) adults (chronological age T1: 20.8 to 29.2 years, T2: 22.8 to 31.8 years). Notably, for DunedinPACE, median deviation cannot be computed as this clock measures pace of aging where a value of 1 indicates aging at the same rate as chronological age. T1 = first pre-pandemic saliva sample, T2 = second pandemic saliva sample

Figure S4. Association between epigenetic and chronological age.

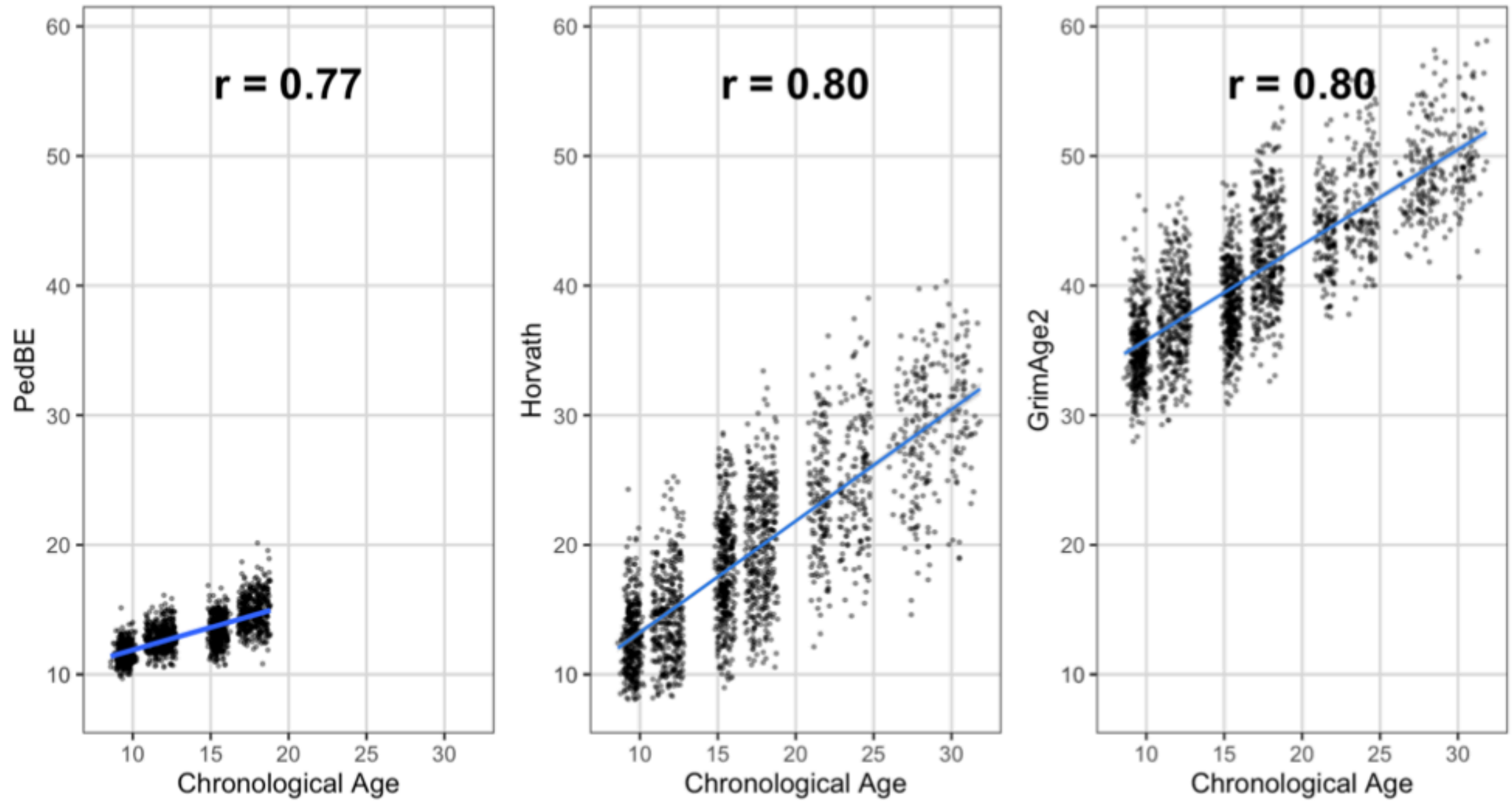

Note. Scatterplots of the epigenetic age and chronological age for each of the four epigenetic clock estimates. Epigenetic age is represented in years. The blue line indicates the predicted epigenetic age based on chronological age using linear regression. Age estimates are clustered by age group and timepoint.

Figure S5. Epigenetic age acceleration and pace of aging by age group.

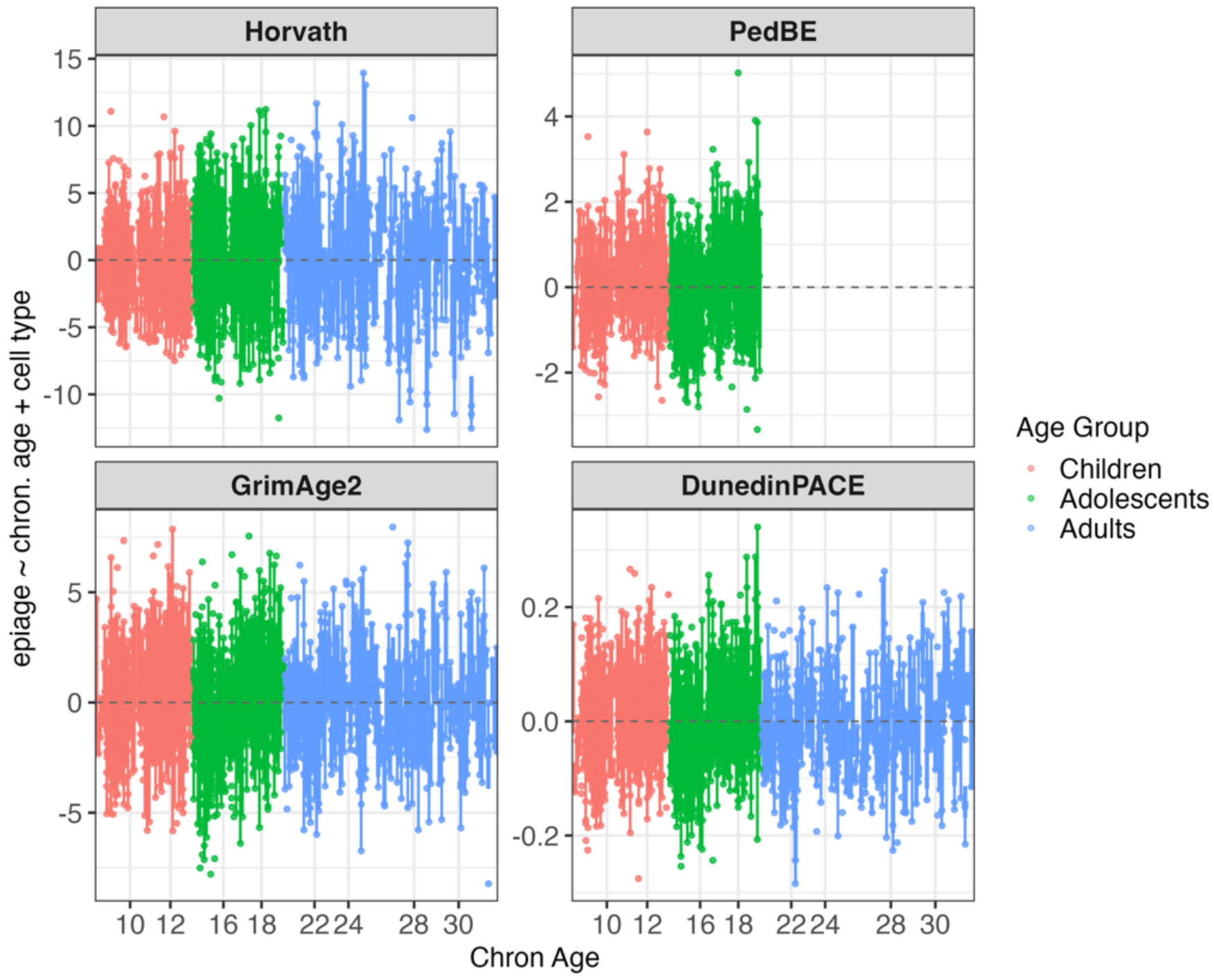

Note. Horvath, PedBE, and GrimAge2 epigenetic age (in years) and Dunedin pace of aging residuals after regressing on chronological age and cell type. Each line indicates the interquartile range (25th–75th percentiles) and the dots denote observations beyond this range (colored by age group) for each chronological age. Observations are colored in red for children, green for adolescents, blue for adults.

Figure S6. Associations between epigenetic clocks at pre-pandemic baseline and follow-up.

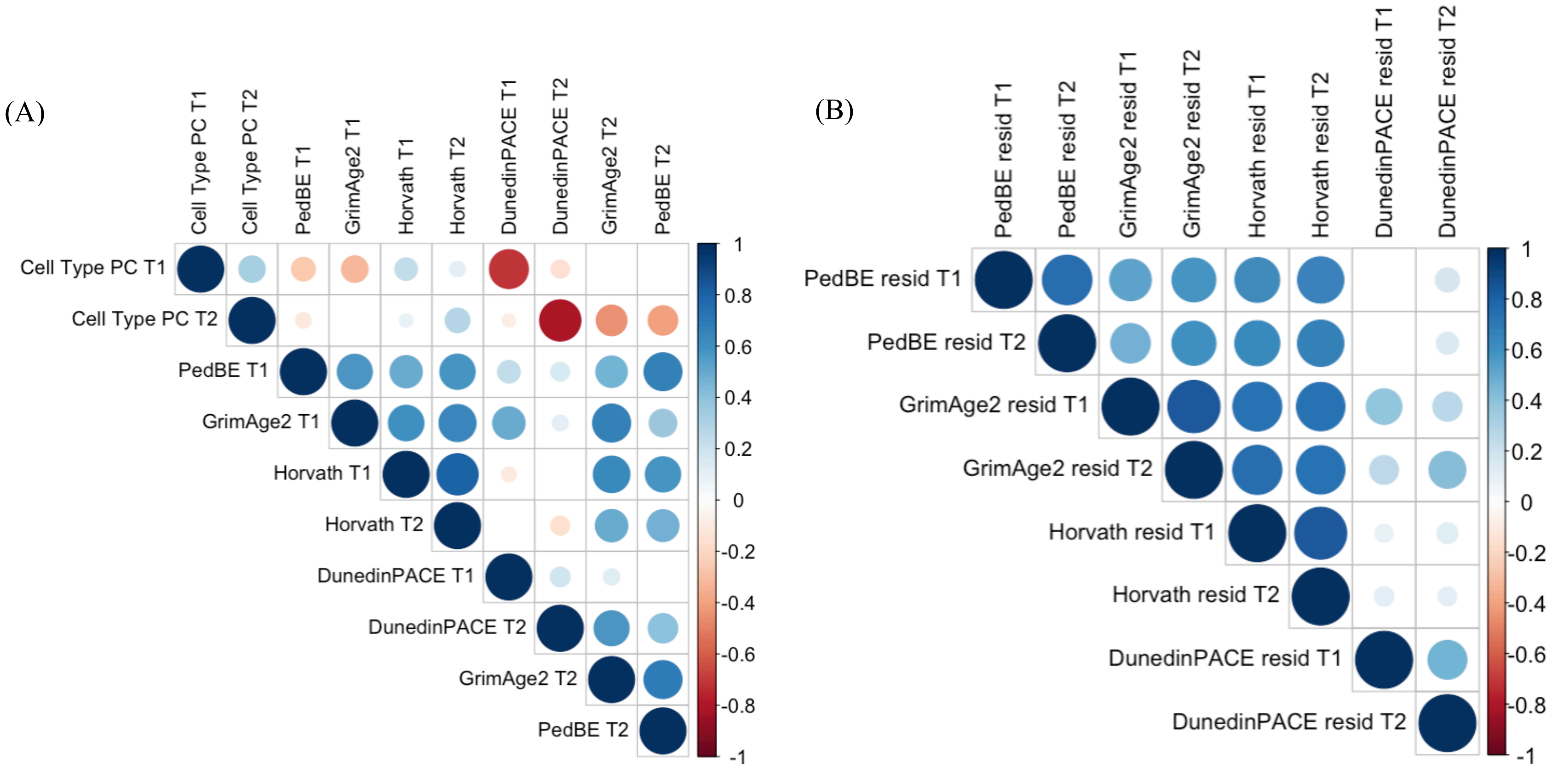

Note. Pearson correlation coefficients ranging from 1 (dark blue) to -1 (dark red) before (A) and after (B) cell type correction. Significant correlations at $p < .05$ are indicated by a coloured circle. Non-significant association are indicated by a blank rectangle. T1 = first pre-pandemic saliva sample, T2 = second pandemic saliva sample, PC = principal component.

2.6 Pandemic stressors: The course of COVID-19 in Germany

The COVID-19 pandemic developed in several phases in Germany,(36, 37) with 7-day incidence rates and an policy stringency index of government-imposed containment measures comparable to other European countries.(38, 39) In Germany, the first infection wave began in March 2020 (CW 10) accompanied by a phase of strict containment measures (1st lockdown) of approx. 2 months (until CW 20) with strict physical distancing guidelines. The following summer of 2020 was characterized by low 7-day incidence rate and less restrictive measures (e.g., obligation to wear a mask in public). The second infection wave began in the fall of 2020 (CW 40) and lasted until February 2021 (CW 8), followed by an immediate third wave until early summer 2021 (CW 23). The second and third infection waves were accompanied by lighter governmental measures (e.g., mandatory COVID-19 testing or access-restrictions for non-vaccinated persons). In June 2021 (CW 24), there was another phase with lower infection rates before the fourth wave started in August 2021 (CW 31), again accompanied by lighter governmental containment measures compared to the beginning of the pandemic, and no new closure of schools or other public places. However, these lighter governmental containment measures towards the end of the pandemic lasted longer and were stricter compared to other European Countries like the United Kingdom which had already lifted all containment measures in June 2021.(39) In April 2023, the German federal government officially declared the end of the COVID-19 pandemic. Figure S6 and S7 show the median 7-day incidence rate and the median policy stringency of government-imposed containment measures for nationwide, and the TECS sample used in our study.

In Germany, most variability in the stringency of COVID-19 measures and mortality burden occurred at the federal state ("Bundesland") level.(40–43) Specifically, while national agreements set a framework, the federal states varied by enacting stricter or more relaxed rules depending on the local conditions. However, the degree of this variability oscillated between more centralized, unified approaches and periods of decentralisation, where states and even local districts had more discretion. For example, local districts could, in principle, apply stricter rules than the federal states under the so-called "incidence rule" if the case numbers exceeded a certain threshold though actual policy differences at this level were less common.

Figure S7. Median 7-Day incidence of confirmed COVID-19 cases in Germany and TECS

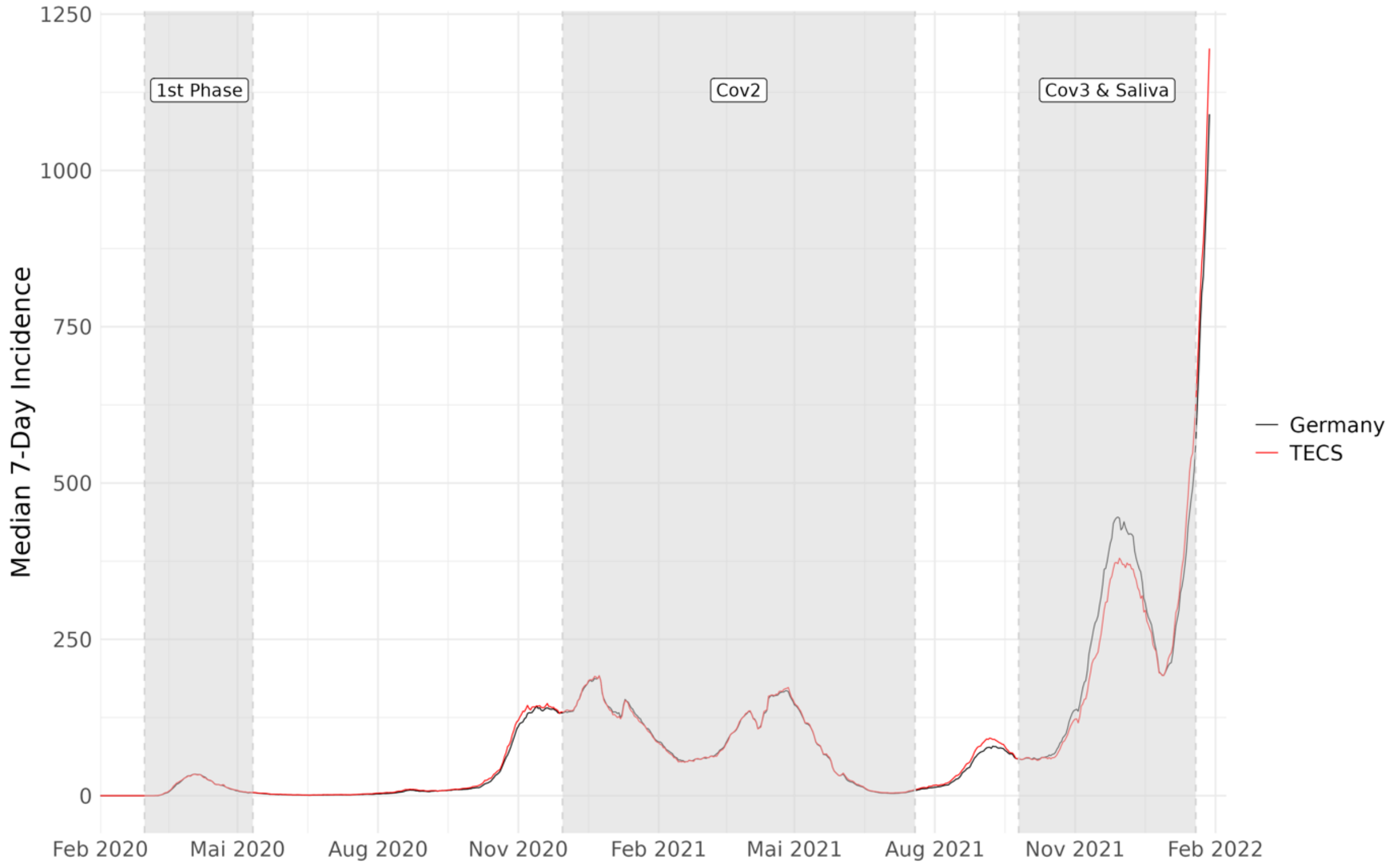

Note. Median 7-day incidence (i.e., the number of reported COVID-19 cases of the last 7 days divided by the number of inhabitants times 100,000) by date from February 2020 to February 2022 across all regions in Germany (black line, AUC = 65,761) and regions represented in the TECS sample of the current study (red line, AUC = 65,445). Light grey areas represent the time interval between the first and last date of the first phase of the COVID-19 pandemic, the first and last date of the Cov2 questionnaire, and the first and last date of the Cov3 and saliva collection. Due to limited testing, the number of confirmed cases is likely lower than the true number of new infections. TECS = TwinLife Epigenetic Change Satellite, AUC = Area Under the Curve.

Figure S8. Median COVID-19 policy stringency in Germany and for the TECS sample.

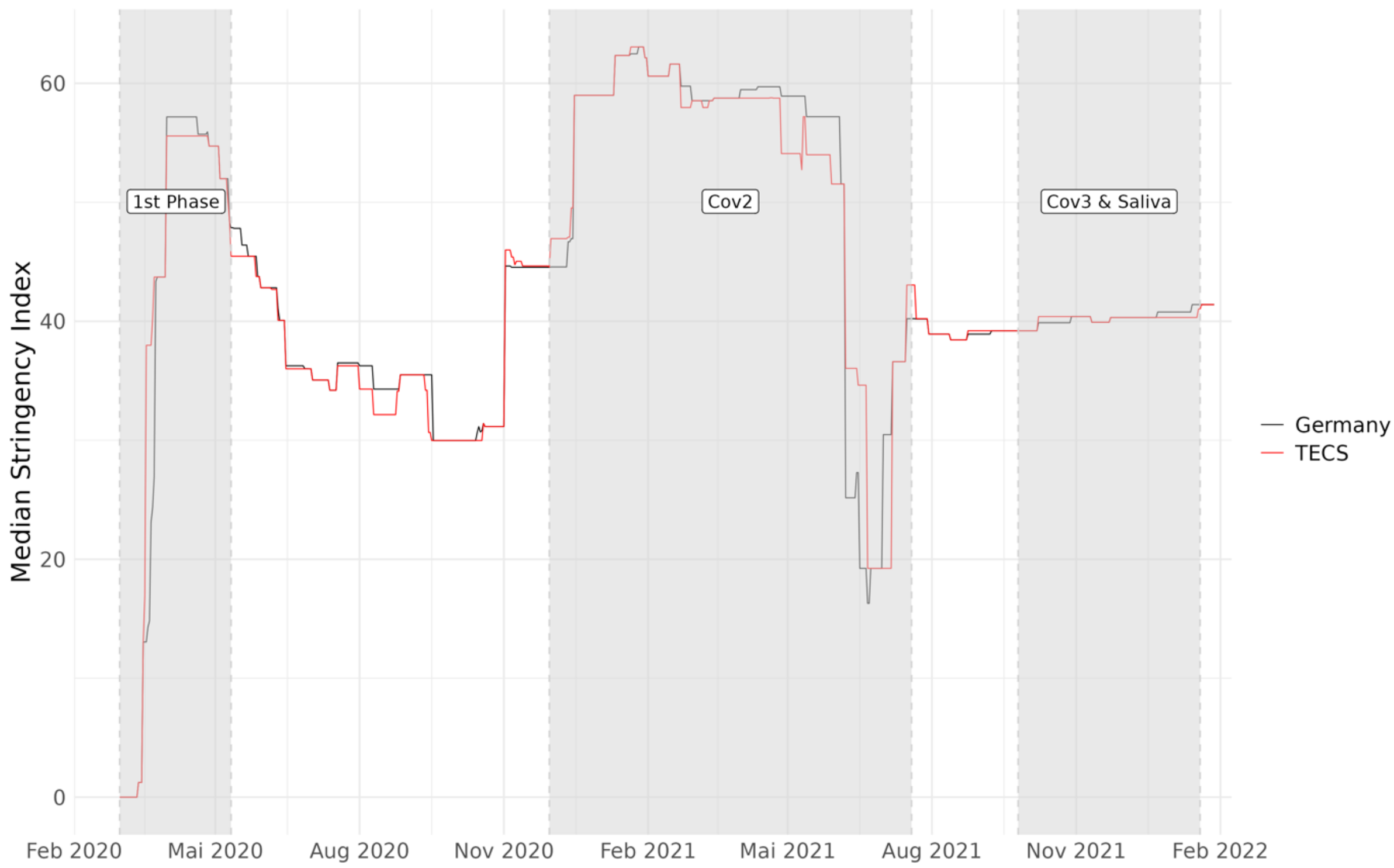

Note. Median COVID-19 stringency index (i.e., policy stringency of government-imposed containment measures across various areas of life, scored on a scale from 0 to 100) by date from February 2020 to February 2022 across all regions in Germany (black line, AUC = 30,122) and regions represented in the TECS sample of the current study (red line, AUC = 30,085). Light grey areas represent the time interval between the first and last date of the first phase of the COVID-19 pandemic, the first and last date of the Cov2 questionnaire, and the first and last date of the Cov3 and saliva collection. TECS = TwinLife Epigenetic Change Satellite, AUC = Area Under the Curve.

2.7 Figure S9. Timing of investigated pandemic stressors in relation to the TwinLife study design

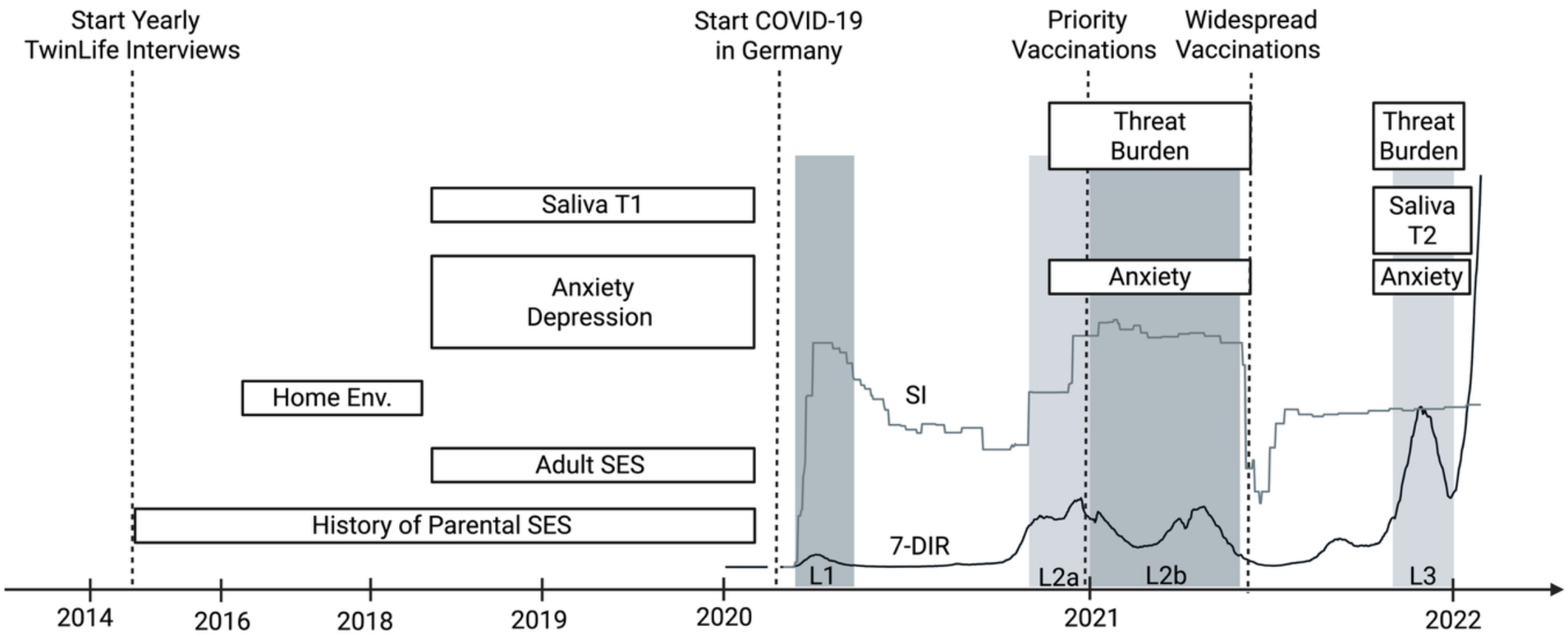

Note. Overview of the timing and measurements included in the present study, from the initiation of the TwinLife study in 2014 up to the second saliva collection. TwinLife families have participated in yearly interviews (see supplementary Figure S1 for a detailed timeline of all surveys). Saliva samples from TwinLife participants were collected through trained interviewers during household interviews for genotyping and DNA methylation assessment (part of the TwinSNPs satellite project) before the first lockdown in Germany on 1st of March 2020. For this study, we excluded the 18 individuals for whom saliva was collected after the 1st of March 2020. A second saliva sample was collected from TwinLife participants via a self-collection kit (Oragene kit, 2 ml), with written instructions enclosed and the collection kit sent by post from September 2021 and May 2022. TwinLife participants were asked to participate in COVID-19 supplementary surveys (i.e., computer-assisted telephone or web interviews), including questions on the subjective burden and threat due to the COVID-19 pandemic, home environment, and anxiety. In Germany, vaccinations were available from the 27th of December 2020 with initial prioritization of groups at risk, including elderly (> 80 years of age), residents and staff of nursing homes and health care workers. From March 2021, vaccinations were gradually available to more groups, like essential works (teachers and police officers) and younger individuals with pre-existing health conditions. From June 6th, 2021, vaccinations were widely available for anyone older than 12 years of age except for specific groups such as pregnant women. Daily 7-day incidence rate and the policy stringency index of government-imposed containment measures for our study period were extracted from public records of the Robert-Koch-Institute (https://github.com/robert-koch-institut/COVID-19_7-Tage-Inzidenz_in_Deutschland) and infas (www.healthcare-datenplattform.de). Lockdown measures in Germany were comparable to other European countries though stricter on unvaccinated individuals from autumn 2021. For more details on the development of the COVID-19 pandemic in Germany, see the SI Appendix, section 3. SES = socioeconomic status, 7-DIR = 7-day incidence rate, SI = stringency index, L1 = first strict lockdown period highlighted in dark grey, L2a = second lockdown period characterized by less strict measures highlighted in light grey, L2b = second lockdown period characterized by stricter measures, L3 = third less strict lockdown mostly characterized by restrictions for unvaccinated individuals. Figure created with BioRender.com.

2.8 Pandemic stressors: Measurement of 7-day incidence rate and policy stringency
As briefly described in the main text, we downloaded the local 7-day incidence rates (i.e., the number of reported COVID-19 cases of the last 7 days divided by the number of inhabitants times 100,000) from the public repository of the Robert-Koch-Institute (RKI)(44) and obtained COVID-19 stringency indices of government-imposed containment measures from the "healthcare daten plattform" maintained by *infas 360 GmbH* (www.healthcare-datenplattform.de). The latter dataset includes date and regional district-specific records of the policy stringency from March 2020 to December 2022. We used the total policy stringency that combines restrictions across various areas of daily life (e.g., public events, closure of restaurants and schools, obligatory face masks) on a scale from 0 to 100. To summarise cumulative exposure to policy stringency and 7-day incidence rate over time, we computed the area under the curve (AUC). This is a data reduction technique that has been shown to be more predictive of health outcomes compared to traditional composite scores of cumulative stress such as sum scores.(45) In total, we computed 8 measures of objective indicators of pandemic stress for our main analysis: Cumulative 7-day incidence rate and policy stringency during the first lockdown (i.e., AUC from March 1st to May 11th 2020), cumulative 7-day incidence rate and policy stringency during the second lockdown (i.e., AUC from November 2nd 2020 to June 1st 2021), cumulative 7-day incidence rate and policy stringency from the start of the pandemic up to the date of the second saliva sampling during the third lockdown, and recent 7-day incidence rate and policy stringency in the 30 days prior to the date of the second saliva sampling. In addition, for our exploratory analyses, we computed recent exposure to 7-day incidence rate and policy stringency in the 30 days prior to the date of Cov2 and Cov3 assessment. The date of Cov2 and Cov3 was based on self-reports and the date of the second saliva collection was based on the date on which the collected saliva reached the survey institute *infas* by post. On average, differences in the objective exposure between Cov3 and the date of the saliva collection were small (see Table S3) and the collected saliva reached *infas* on average 4.3 days (median = 4 days) after the date of the Cov3 questionnaire completion, although the saliva postal date ranged from 28 days prior to 115 days after the Cov3 self-reports completion.

2.9 Pandemic stressors: Self-reported pandemic threat and burden
All items of the supplementary COVID-19 questionnaires in TwinLife can be found at https://paneldata.org/twinlife/datasets/. Items defining perceived threat originated from the Mannheim Corona Study (MCS).(46) On a scale ranging from 1 "no threat to me at all" to 10 "extreme threat to me" participants were asked to rate the pandemic regarding their health, school performance, and social relationships (3 items, cohort 1) or their health, economic situation, opportunities for education and training, personal freedom, social life and occupational opportunities (6 items, cohort 2 to 4). The item on occupational opportunities was excluded for the analyses in this study as the number of valid answers from twins was very low in all three cohorts. All items were positively correlated at Cov2 (r = 0.14 to r = 0.61) and Cov3 (r = 0.29 to r = 0.66).

The measurement of pandemic related burdens was adapted from the questionnaire of the study "Psychological and physical long-term effects around COVID-19".(47) On a scale ranging from 1 "not bad at all" to 10 "very bad", children rated the burden of the COVID-19 pandemic in terms of social isolation, lack of sporting activity, irregular daily routine and home schooling at Cov2 (4 items). All other participants reported the burden of the pandemic regarding social isolation, separation from important people, lack of leisure activities, unregulated daily schedule, and restriction of civil rights at Cov2 (5 items). At Cov3, all participants additionally rated the burden of wearing face masks in public, regular viral testing, and the COVID-19 pandemic in general up to the time of the survey (in total, 6 items for children and 7 for all other participants). All items were positively correlated at Cov2 (r = 0.28 to r = 0.64) and at Cov3 (r = 0.18 to r = 0.71).

To generate a single measure of perceived COVID-19 threat and burden, we computed an average score across all items of the respective scale for each participant at Cov2 and Cov3 (see Table S3 for means and standard deviations).

2.10 Covariates.
We used DNA methylation values to estimate cell type composition, smoking, exposure, and BMI. Cell type composition was estimated using the *epidish*() function of the R-package EpiDish (version 2.10).(48, 49) Since the three cell types composing saliva tissue (i.e., leukocytes, epithelial cells, and fibroblasts) are highly correlated, we obtained the first principal component (for each occasion separately, explaining 99.6% and 99.7% of cell type variation at baseline and follow-up, respectively) to control for cell type in all our analyses. An epigenetic marker of BMI was computed based the

weighted sum of 369 out of a total of 397 (92.9%) CpG sites that were significantly associated with BMI in an epigenome-wide association study by Do et al.(50) The resulting epigenetic marker was significantly associated with BMI, measured at F2F3 in our sample (children: $r = 0.19$, p = 0.0003; adolescents: $r = 0.21$, p < 0.0001; adults: $r = 0.26$, p < 0.0001). For epigenetic smoking exposure(51), we reverse coded the beta values of the cg05575921 probe, so that a higher score indicates greater smoking exposure. In addition, genetic sex and BMI was obtained from the TwinLife survey at first saliva sampling. Chronological age at sampling was computed was saliva collection month/year minus birth month/year.

2.11 Demographic background and risk factors

Demographic and risk factors were obtained from the TwinLife surveys. All individual questionnaire items of the household surveys from all survey waves are provided at https://paneldata.org/twinlife/. In addition, the TwinLife scales manual provides a detailed description of all scales used in this study.(52)

Genetic sex. The genetic sex was defined based on self-reported sex documented at pre-pandemic baseline and genetically confirmed using the individual genotype data. Information of self-reported gender identity at pre-pandemic baseline or pandemic follow-up was not available for this study.

BMI. In F2F1 and F2F2 participants were asked to fill in their current height [in cm] and weight [in kg] in an open-ended format. For children < 14 years, one parent was asked to provide the child's current height and weight. Data collection and quality control are described in Klatzka et al.(53). From F2F3 onwards, current height and weight was only provided for children < 13 years. For this study, we included BMI (kg/m2) as a covariate in our analyses based on the most recent available report at F2F3 for each participant.

Socioeconomic status. To include a measure of the history of available parental socioeconomic resources as a potential protective factor of COVID-19-related adversity, we computed a composite score of socioeconomic status (SES) based on the average parental highest education (highest degree transformed to years of education), parental highest occupation (based on the international socio-economic index of occupational status [ISEI])(54), and household net income (HH income) from F2F1 to F2F3 (shortly before the COVID-19 pandemic, see Figure S1). At each assessment, all three measures were first z-score transformed and then summed up to a single composite score of SES (if any of the three measures was missing, the composite SES was set to NA) and then averaged across all assessments. Moreover, for twins who had already moved out of their parental home at F2F3, we computed their individual SES by summing up their z-score transformed personal gross income, occupation (based on the ISEI), and highest degree of education (in years) measured at F2F3 (if any of the three measures was missing, the composite SES was set to NA).

Home environment. Perceived household chaos as indicator of the home environment was measured with an adapted version of the Chaos, Hubbub and Order Scale (CHAOS).(55) This scale assesses the degree of "environmental confusion" (e.g., noise or crowding) in children's homes and can be used to rate chaotic home environments. The most recent assessment of the home environment before the COVID-19 pandemic was F2F2 (see Figure S1). For this study, we only included self-report items of the twins themselves. Five statements regarding the atmosphere at home (e.g., "It's a real zoo in our home.", "You can't hear yourself think in our home.") were rated by participants up to 21 years of age and participants still living with their parents on a scale from "1 not correct at all" to "5 fully correct". For children up to 13 years of age at F2F2, the CHAOS scale included a sixth item ("We have the same bedtime routine every night"). Positively phrased statements indicating the regular bed routine, calm atmosphere, and order were revers coded before averaging scores across all items, so that a higher score indicates more chaos at home. After reverse coding, all times were positively correlated, ranging from $r = 0.01$ to $r = 0.49$.

Anxiety symptoms. In TwinLife, the items used to measure anxiety symptoms were adapted from the GAD-7 for generalized anxiety disorder.(56) At pre-pandemic baseline (F2F3) and during the second lockdown (Cov2), participants (only those > 11 years) rated their level of nervousness and control over worrying on a scale from "1 not at all" to "4 almost every day". During the third lockdown (Cov3), also the youngest cohort was asked to provide answers to these items. At each timepoint, we computed an average score across the two items for each participant. If one item was missing, we set

the mean to missing. The two items were positively correlated at pre-pandemic baseline (*r* = 0.51), Cov2 (*r* = 0.58), and Cov3 (*r* = 0.59, youngest cohort *r* = 0.46).

Depressive symptoms. TwinLife items measuring depressive symptoms were inspired by the German adaption of Becks Depression Inventory – Fast Screen (BDI-FS).(57) However, the response format was changed. At pre-pandemic baseline (F2F3), participants (only those > 11 years) rated seven items regarding different aspects of their emotional states in the last two weeks on a scale from "1 never" to "4 almost always". All items were positively correlated (between *r* = 0.26 and *r* = 0.63) and computed as an average score for each participant.

2.12 Survey items

Table S2 shows the individual questionnaire items and response options that were included in the present study in the order they were assessed, both in their original language (German) and translated to English. A complete overview of all individual questionnaire items of the household surveys from all survey waves is provided at https://paneldata.org/twinlife/.

Table S2. Individual survey items.

Construct	Survey Wave	Question:<br>German original<br>*English translation*	Item:<br>German original<br>*English translation*	Response Options:<br>German original<br>*English translation*
Home environment	F2F2	Im Folgenden moechte ich gerne mit dir ueber Dein Zuhause sprechen. Bitte gib an, inwiefern diese Saetze auf Dein Zuhause zutreffen. Antworte bitte anhand der folgenden Skala. Der Wert 1 bedeutet: stimmt ueberhaupt nicht. Das waere dann der 'Daumen runter'. Der Wert 5 bedeutet: stimmt genau. Das waere dann der 'Daumen hoch'. Mit den Werten zwischen 1 und 5 kannst Du mir wieder sagen, wenn deine Meinung irgendwo dazwischen liegt.<br><br>*In the following I would like to talk to you about your home. Please indicate how these sentences apply to your home. Please answer using the following scale. The value 1 means: not true at all. This would be a 'thumbs down'. A value of 5 means: exactly right. That would be the 'thumbs up'. With the values between 1 and 5 you can tell me again if your opinion is somewhere in between.*	1. Bei uns läuft das Schlafengehen jeden Abend gleich ab (z.B. jeden Abend dieselbe Schlafenszeit, ein Bad vorm Schlafengehen, eine Geschichte (vor-)lesen, ein Gebet sprechen...).<br>*1. We have the same bedtime routine every night (e.g. going to bed at the same time every evening, having a bath before going to bed, reading a story, saying our prayers ...).*	Likert scale from 1 to 5, with:<br><br>1 = Stimmt überhaupt nicht<br>*1 = Not true at all*<br><br>5 = Stimmt genau<br>*5 = Exactly right*
			2. Bei uns zuhause kann man nicht in Ruhe nachdenken.<br>*2. You can't hear yourself think in our home.*	
			3.Bei uns zuhause laeuft alles sehr wild und ungeordnet.<br>*3.Everything is very wild and disorganized in our house.*	
			4. Normalerweise haben wir zuhause alles im Griff.<br>*4. We are usually able to stay on top of things.*	
			5. Irgendwo bei uns im Haus laeuft fast immer ein Fernseher.<br>*5. There is usually a television turned on somewhere in our home.*	
			6. Bei uns zuhause ist die Stimmung ruhig und entspannt.<br>*6. The atmosphere in our home is calm.*	
Depressive symptoms	F2F3	Manchmal fuehlt man sich niedergeschlagen, traurig oder angespannt. Wir interessien uns dafuer,	1. Ich bin traurig.<br>*1. I am sad.*	
			2. Ich sehe mutlos in die Zukunft.<br>*2. I am pessimistic about my future.*	

		wie das bei Dir/Ihnen ist. Wie haeufig treffen folgende Aussagen bezogen auf die letzten zwei Wochen zu?<br><br>*Sometimes you feel down, sad or tense. We are interested in what this is like for you. Over the past two weeks, how often have the following statements applied to you?*	3. Ich fuehle mich als Versager/in.<br>*3. I feel like a failure.*<br><br>4. Es fällt mir schwer, etwas zu genießen.<br>*4. I find it difficult to enjoy anything.*<br><br>5. Ich bin von mir enttäuscht.<br>5. *I am disappointed in myself.*<br><br>6. Ich werfe mir Fehler und Schwächen vor.<br>*6. I blame myself for mistakes and weaknesses.*	Likert Scale from 1 to 4, with:<br><br>1 = Nie<br>*1 = Never*<br><br>4 = Immer<br>*4 = Almost always*
Anxiety Symptoms	F2F3	Wie oft fuehltest Du Dich im Verlauf der letzten zwei Wochen durch die folgenden Beschwerden beeintraechtigt?<br><br>*During the last two weeks, how often did you feel affected by the following complaints?*	1. Nervositaet, Aengstlichkeit oder Anspannung.<br>*1. Nervousness, anxiety or tension*<br><br>2. Nicht in der Lage sein, Sorgen zu stoppen oder zu kontrollieren.<br>*2. Not being able to stop or control worries.*	1 = Nie<br>*1 = Never*<br><br>2 = An einzelnen Tagen<br>*2 = On single days*<br><br>3 = Mehr als die Hälfte der Tage<br>*3 = More than half of the days*<br><br>4 = Fast jeden Tag<br>*4 = Almost every Day*
	Cov2	Wie oft fuehlen Sie sich derzeit durch die folgenden Beschwerden beeintraechtigt?<br><br>*How often do you currently feel affected by the following complaints?*	1. Nervositaet, Aengstlichkeit oder Anspannung.<br>*1. Nervousness, anxiety or tension.*<br><br>2. Nicht in der Lage sein, Sorgen zu stoppen oder zu kontrollieren.<br>*2. Not being able to stop or control worries.*	
	Cov 3	Age >= 16 years:<br>Wie oft fuehlen Sie sich derzeit durch die folgenden Beschwerden beeintraechtigt?<br><br>*How often do you currently feel affected by the following complaints?*<br><br>Age <=15 years:<br>Wie oft hast du Dich in den letzten Tagen so gefuehlt?	1. Nervosität, Ängstlichkeit oder Anspannung.<br>*1. Nervousness, anxiety or tension.*<br><br>2. Nicht in der Lage sein, Sorgen zu stoppen oder zu kontrollieren.<br>*2. Not being able to stop or control worries.*	

		How many times have you felt like this in the last few days?		
Perceived Burden	Cov2	Aage >= 16 years:<br>Die Corona-Pandemie betrifft auch weiterhin den Alltag vieler Menschen. Sofern Sie die folgenden Einschraenkungen derzeit erleben, wie sehr belasten Sie diese? Sie koennen Ihre Antworten von 1 “Keine Belastung” bis 10 “Extreme Belastung” abstufen.<br><br>*The COVID-19 pandemic continues to affect the daily lives of many people. To the extent that you are currently experiencing the following limitations, how much of a burden do they have on you? You can grade your answers from 1 “no burden” to 10 “extreme burden”.*	1. Soziale Isolation / Einsamkeit<br>*1. Social isolation / loneliness*	Likert scale, with:<br><br>1 = Keine Belastung<br>*1 = No burden*<br><br>10 = Extreme Belastung<br>*10 = Extreme burden*
			2. Getrenntsein von wichtigen Personen<br>*2. being separated from important people*	
			3. fehlende Freizeitaktivitaeten wie Sport<br>*3. lack of leisure activities such as sports*	
			4. ungeregelter Tagesablauf / Wochenrhythmus<br>*4. unregulated daily routine / weekly rhythm*	
			8. Einschraenkung von Buergerrechten<br>*8. restriction of civil rights*	
		Age <= 15 years:<br>Die Corona-Pandemie betrifft auch weiterhin den Alltag vieler Menschen. Sofern Du die folgenden Einschraenkungen derzeit erlebst, wie schlimm sind diese fuer Dich? Du kannst Deine Antworten von 1 "Gar nicht schlimm" bis 10 "Sehr schlimm" abstufen.<br><br>*The COVID-19 pandemic continues to affect the daily lives of many people. If you are currently experiencing the following limitations, how bad are they for you? You can grade your answers from 1 "not bad at all" to 10 "very bad".*	1. Freunde und Verwandte seltener sehen<br>*1. See friends and relatives less often*	Likert scale, with:<br><br>1 = Schlimm<br>*1 = Not bad at all*<br><br>10 = Sehr schlimm<br>*10 = Very bad*
			2. Nicht mehr so oft zum Sport gehen<br>*2. No longer go to the gym so often*	
			3. Einen ungeregelten Tagesablauf haben<br>*3. Have an unregulated daily routine*	
			4. Schulunterricht (auch) zu Hause zu haben<br>*4. To have schooling (also) at home*	
	Cov3	If age >= 16 years:	1. Soziale Isolation / Einsamkeit<br>*1. Social isolation / loneliness*	Likert scale, with:

		Die Corona-Pandemie betrifft auch weiterhin den Alltag vieler Menschen. Sofern Sie die folgenden Einschraenkungen derzeit erleben, wie sehr belasten Sie diese? Sie koennen Ihre Antworten von 1 "Keine Belastung" bis 10 "Extreme Belastung" abstufen.<br><br>*The COVID-19 pandemic continues to affect the daily lives of many people. To the extent that you are currently experiencing the following limitations, how much of a burden do they have on you? You can grade your answers from 1 "no burden" to 10 "extreme burden"*	2. Getrenntsein von wichtigen Personen<br>*2. Being separated from important people*	1 = Keine Belastung<br>*1 = No burden*<br><br>10 = Extreme Belastung<br>*10 = Extreme burden*
			3. Fehlende Freizeitaktivitaeten wie Sport<br>*3. Lack of leisure activities such as sports*	
			4. Ungeregelter Tagesablauf / Wochenrhythmus<br>*4. Unregulated daily routine / weekly rhythm*	
			8. Einschraenkung von Buergerrechten<br>*8. Restriction of civil rights*	
			9. Maskenpflicht<br>*9. Compulsory mask-wearing*	
			10. Corona-Testpflicht<br>*10. mandatory corona testing*	
		If age >= 16 years:<br>Alles in allem, wie stark hat Sie die Corona-Pandemie mit all ihren Auswirkungen bisher belastet?<br><br>Overall, how much of a burden has the COVID-19 pandemic and all its implications been on you so far?	1. Die Corona Pandemic insgesamt<br>*1. The pandemic overall*	
		If age <= 15 years:<br>Die Corona-Pandemie betrifft auch weiterhin den Alltag vieler Menschen. Sofern Du die folgenden Einschränkungen derzeit erlebst, wie schlimm sind diese für Dich? Du kannst Deine Antworten von 1 "Gar nicht schlimm" bis 10 "Sehr schlimm" abstufen.	1. Freunde und Verwandte seltener sehen<br>*1. See friends and relatives less often*	Likert scale, with:<br><br>1 = Schlimm<br>*1 = Not bad at all*<br><br>10 = Sehr schlimm<br>*10 = Very bad*
			2. Nicht mehr so oft zum Sport gehen<br>*2. No longer go to the gym so often*	

		The COVID-19 pandemic continues to affect the daily lives of many people. If you are currently experiencing the following limitations, how bad are they for you? You can grade your answers from 1 "not bad at all" to 10 "very bad".	3. Einen ungeregelten Tagesablauf haben<br>*3. Have an unregulated daily routine*	
			4. Schulunterricht (auch) zu Hause zu haben<br>*4. To have schooling (also) at home*	
Perceived Threat	Cov2, Cov3	If age >= 16 years:<br>Wie sehr empfinden Sie die Corona-Pandemie als Bedrohung fuer... Sie können Ihre Antworten von 1 “Keine Bedrohung” bis 10 “Extreme Bedrohung” einstufen.<br><br>*How much do you perceive the COVID-19 pandemic as a threat to... You can grade your answers from 1 'no threat to me at all' to 10 'extreme threat to me'.*	1. Ihre Gesundheit<br>*1. Your health*	Likert scale, with:<br><br>1 = Keine Bedrohung<br>*1 = No threat at all*<br><br>10 = Extreme Bedrohung<br>*10 = Extreme threat to me*
			2. Ihre wirtschaftliche Lage<br>*2. Your economic situation*	
			3. Ihre Chancen auf Aus- und Weiterbildung<br>*3. Your opportunities for education and training*	
			4. Ihre perönliche Freiheit<br>*4. Your personal freedom*	
			5. Ihr Sozialleben (z.B. Ihre Moeglichkeiten neue Leute kennen zu lernen, bestehende Kontakte zu pflegen)<br>*5. Your social life (e.g., your possibilities for meeting new people, maintaining existing contacts)*	
	Cov2, Cov3	If age <= 15 years:<br>Wie sehr empfindest Du das Coronavirus als Bedrohung fuer... Du kannst Deine Antworten von 1 “Keine Bedrohung” bis 10 “Extreme Bedrohung” einstufen.<br><br>*How much do you perceive the coronavirus as a threat to... You can grade your answers from 1 'no threat to me at all' to 10 'extreme threat to me'.*	1. Ihre Gesundheit<br>*1. Your health*	
			2. Deine Leistungen in der Schule<br>*2. Your performance at school*	
			3. Deine Beziehungen zu anderen Menschen<br>*3. Your relationships with other people*	
Note: In F2F waves, survey questions were administered by interviewers in the respective household. The Covid-19 supplementary surveys were administered in an online-telephone mixed format or a paper-pencil-online mixed format.				

3. Statistical analysis

All analyses were run in R (version 4.1.3, 2022-03-10).(58) Each of the analyses detailed below were run in the full sample ("All"), as well as stratified by age group. Since items for perceived threat and burden due to the COVID-19 pandemic differed between children and the other participants, full sample analysis of these subjective pandemic stressors was only combined for adolescents and adults. To obtain standardized beta coefficients, all variables were scaled to a mean of zero and a standard deviation of 1 prior to analysis.

3.1 R-packages

Please note that this is not a comprehensive list and only includes the main packages used for analysis. For a comprehensive overview of all packages and analysis code, please see analysis_code_osf.io.

Table S3. R-packages.

Package	Analysis
coxme(59)	lmekin() function used to run regression analyses controlling for genetic relatedness
ggplot2(60), patchwork(61), corrplot(62)	Data visualization
dplyr(63), data.table(64), tibble(65), tidyverse(63), gtsummary(66)	Data processing
poolr(67)	Multiple testing correction using spectral decomposition according to Li and Ji(68)

3.2 Effective number of independent tests

We applied multiple testing correction using spectral decomposition according to Li and Ji(68). This method estimates the effective number of independent tests (M_{eff}) based on the eigenvalues of a correlation matrix. This correlation matrix can be either based on the correlations between individual observations of the tested variables or correlations between tests statistics across several predictors and outcomes. Multiple testing correction is then applied by multiplying the original p-value by M_{eff}.

In our first analysis (i.e., testing for change in biological aging within individuals), we determined M_{eff} based on the correlation between our four epigenetic clocks across all timepoints and individuals, resulting in the adjustment with an $M_{eff} = 4$. For all subsequent analyses, we always had multiple predictors and outcomes and could thus derive M_{eff} from the correlation matrix of actual test statistics (i.e., the standardized beta coefficients). For our main analysis of pandemic stressors and epigenetic aging, this resulted in an $M_{eff} = 9$. In our exploratory analysis of risk factors and epigenetic aging at pre-pandemic baseline, pandemic follow-up, and change in epigenetic aging, we calculated an $M_{eff} = 11$. Finally, for our exploratory analysis of the effects of objective stressors and risk factors on subjective stressors and mental well-being, we calculated an $M_{eff} = 8$.

3.3 Model equations

All our analyses are based on linear mixed-effects models using *lmekin* from the coxme package.(59) The directed acyclic graph (DAG) in Figure S10 provides an overview of the relationships examined using correlational analyses.

Figure S10. Simplified DAG of hypothesized relationships.

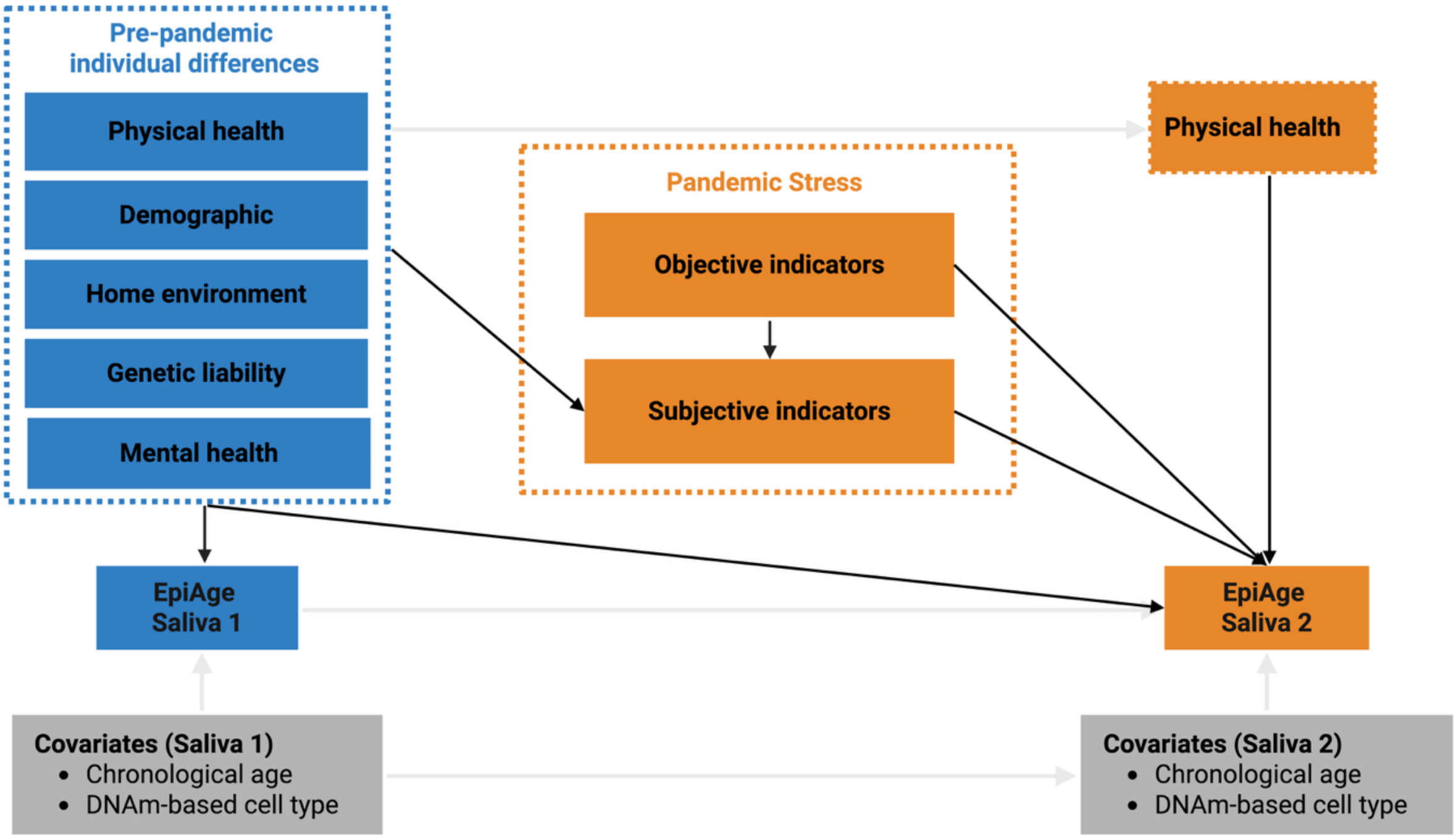

Note. The simplified directed acyclic graph (DAG) provides an overview of all hypothesized that we examined in linear mixed models (see model equations in section 3.3 for detailed statistical analysis). Tested associations are illustrated with black arrows. The grey arrows indicate associations we correct for in our analyses such as the effect of cell type and chronological age. Though hypotheses are directed, all analyses were correlational so that we cannot infer causal relationships. The blue squares refer to any pre-pandemic assessment, the orange squares to assessments during the COVID-19 pandemic, and the grey squares to covariates. EpiAge represents the epigenetic age derived from four epigenetic clocks: Horvath DNAmAge, Pediatric-buccal-epigenetic clock, GrimAge2, and DunedinPACE (see section 2.4). A description of individual variables is provided in section 2.8 to 2.11, with pre-pandemic physical health: BMI, DNAm-based BMI, and DNAm-based smoking exposure; demographic background: genetic sex, parental SES, and personal SES (for adults); pre-pandemic home environment: home chaos, city size, living with parents, genetic liability: PGS for well-being; pre-pandemic mental health: anxiety and depressive symptoms; objective indicators: 7-day incidence rate and policy stringency during the first, second, and third lockdown; subjective indicators: perceived pandemic threat and burden during the second and third lockdown; and physical health during the pandemic: DNAm-based BMI and smoking exposure. BMI = body-mass-index, SES = socioeconomic status, DNAm = DNA methylation.

Change in epigenetic aging. To test whether biological age changed during the COVID-19 pandemic, we used epigenetic age as the dependent variable and included time, chronological age, cell type proportions, and sex as fixed effects (Model 1). We repeated the analysis including epigenetic smoking and pre-pandemic BMI as additional covariates (Model 2) to examine whether the observed change remained significant. Time was coded as a continuous variable in years, ranging from pre-pandemic baseline (coded as 0) to pandemic follow-up (between 1.5 and 3 years), because the pre-pandemic saliva collection date differed between individuals (December 2018 to March 2020). A positive coefficient for time indicates an increase and a negative coefficient indicates a decrease in age acceleration or pace of aging from pre-pandemic baseline to pandemic follow-up. Please note that epigenetic age, chronological age, epigenetic smoking, and cell type proportions are time varying variables, varying within individuals over time *t,* as well as between individuals *i*. Time, sex, and BMI are time invariant covariates, varying between individuals *i*. For each epigenetic clock, we fitted:

Model 1:
$$Y_{it} = \beta_0 + \beta_1 ChronAge_{it} + \beta_2 CellType_{it} + \beta_3 Sex_i + \boldsymbol{\beta_4 Time_i} + u_i + \epsilon_{it}$$

Model 2:
$$Y_{it} = \beta_0 + \beta_1 ChronAge_{it} + \beta_2 CellType_{it} + \beta_3 Sex_i + \boldsymbol{\beta_4 Time_i} + \beta_5 BMI_i + \beta_6 Smoke_{it} + u_i + \epsilon_{it}$$

with
Y_{it} = Repeated measure of epigenetic age of individual i per timepoint t
β_0 = Intercept
β_1 = Effect of chronological age for individual i per timepoint t
β_2 = Effect of cell type for individual i per timepoint t
β_3 = Effect of biological sex for individual i, reference group = male
β_4 = Effect of time for individual i
β_5 = Effect of pre-pandemic body-mass-index (BMI) for individual i
β_6 = Effect of smoking for individual i per timepoint t

$u_i \sim N(0, \sigma^2 G)$ = Individual-specific random intercept variance, where **G** is the genetic relatedness matrix (GRM, i.e., the between individual variance-covariance matrix is weighted by kinship coefficients)
$\epsilon_{it} \sim N(0, \sigma^2)$ = Residual unexplained within- and between individual variance

Pandemic stressors and change in epigenetic aging. To investigate whether between-individual changes in epigenetic aging were associated with pandemic-related stressors, we included an interaction between time and the respective stressor (i.e., time invariant covariates, varying between individuals *i*). Please note that pandemic stressors did not exist at pre-pandemic baseline, and the respective stressor is therefore a time invariant variable measured during the first, second, or third lockdown. The interaction effect (time*stressor) indicates the effect per stressor on epigenetic age acceleration throughout the COVID-19 pandemic. For each epigenetic clock and each subjective stressor, we fitted:

Model 1:
$$Y_{it} = \beta_0 + \beta_1 ChronAge_{it} + \beta_2 CellType_{it} + \beta_3 Sex_i + \beta_4 Time_i + \boldsymbol{\beta_5 Time_i * Stressor_i} + u_i + \epsilon_{it}$$

Model 2:
$$Y_{it} = \beta_0 + \beta_1 ChronAge_{it} + \beta_2 CellType_{it} + \beta_3 Sex_i + \beta_4 Time_i + \boldsymbol{\beta_5 Time_i * Stressor_i} + \beta_6 BMI_i + \beta_7 Smoke_{it} + u_i + \epsilon_{it}$$

with
Y_{it} = Repeated measure of epigenetic age of individual i per timepoint t
β_0 = Intercept
β_1 = Effect of chronological age for individual i per timepoint t
β_2 = Effect of cell type for individual i per timepoint t
β_3 = Effect of biological sex for individual i, reference group = male
β_4 = Effect of time for individual i
β_5 = Effect of stressor for individual i on change in epigenetic age

β_6 = Effect of pre-pandemic body-mass-index (BMI) for individual i
β_7 = Effect of smoking for individual i per timepoint t

$u_i \sim N(0, \sigma^2 G)$ = Individual-specific random intercept variance, where **G** is the genetic relatedness matrix (GRM; i.e., between individual variance-covariance matrix is weighted by kinship coefficients)
$\epsilon_{it} \sim N(0, \sigma^2)$ = Residual variance; unexplained within (*t*)- and between (*i*)-individual

Individual risk factor, pandemic stressors, and epigenetic age before and during the pandemic. We explored the effects of risk factors on epigenetic aging by examining their main effects on epigenetic age at pre-pandemic baseline and pandemic follow-up separately as dependent variable. For pandemic stressors, we only tested for effects at pandemic follow-up. For each epigenetic clock, pandemic stressor, risk factor and timepoint we fitted:

$$Y = \beta_0 + \beta_1 ChronAge + \beta_2 CellType + \beta_3 Sex + \boldsymbol{\beta_4 Ind.Risk} + u$$

with
Y = Epigenetic age
β_0 = Intercept
β_1 = Effect of chronological age
β_2 = Effect of cell type
β_3 = Effect of biological sex, reference group = male
β_4 = Effect of risk factor

$u \sim N(0, \sigma^2 G)$ = Random intercept variance, where **G** is the genetic relatedness matrix (GRM; i.e., variance-covariance matrix is weighted by kinship coefficients)

Individual risk factors, and objective pandemic stressor effects on anxiety pandemic threat and burden. We tested the effects of several factors (i.e., sex, parental or personal SES [for adults], still living at parental home [for adults], city size, home environment, WBS PGS, anxiety, depressive symptoms, pre-pandemic BMI, epigenetic smoking and epigenetic BMI) separately on pre-pandemic anxiety and depressive symptoms, as well as anxiety, perceived pandemic threat and burden during the second and third lockdown, controlling for age and sex. In addition, we tested the effects of objective pandemic stressors (7-day incidence rate and policy stringency from the first, second, and third lockdown on anxiety, perceived pandemic threat and burden during the second and third lockdown. For each outcome Y (i.e., pre-pandemic anxiety and pre-pandemic depressive symptoms, as well as anxiety, perceived pandemic threat and burden during the second and third lockdown), we fitted:

$$Y = \beta_0 + \beta_1 ChronAge + \beta_2 Sex + \boldsymbol{\beta_3 Predictor} + u$$

with
Y = Outcome
β_0 = Intercept
β_1 = Effect of chronological age
β_2 = Effect of biological sex, reference group = male
β_3 = Effect of predictor

$u \sim N(0, \sigma^2 G)$ = Random intercept variance, where **G** is the genetic relatedness matrix (GRM; i.e., variance-covariance matrix is weighted by kinship coefficients)

4. Results

4.1 Descriptive statistics

Table S4 shows the means and standard deviations of the objective and subjective stressors investigated in the current study for children, adolescents, and adults. Cumulative objective pandemic stress was defined as the area under the curve (AUC) of either 7-day incidence or the policy stringency index of government-imposed containment measures from the start of the pandemic in Germany (in March 2020) up to the respective date of sampling. Recent objective pandemic stress was defined as the AUC from 30 days prior to the respective date of sampling up to the date of sampling. In addition, we computed lockdown specific pandemic stress as the AUC from the start to the end date of the of the respective lockdown. Table S5 shows the means and standard deviations for pre-pandemic home environment, and anxiety and depressive symptoms, as well as anxiety during the second and third lockdown. Overall, scores of all the examined self-reported scales indicated low to moderate average levels of psychological distress before and during the COVID-19 pandemic in our sample.

Table S4. Descriptive statistics of COVID-19 pandemic subjective and objective pandemic stressors in the TECS sample

	1st phase M (SD)	Cov2 M (SD)	Cov3 M (SD)	Second saliva sampling M (SD)
**Perceived threat**				
Children		4.02 (1.92)	3.55 (1.74)	
Adolescents		4.49 (1.55)	4.29 (1.68)	
Adults		4.91 (1.37)	4.78 (1.58)	
**Perceived burden**				
Children		5.10 (1.86)	4.45 (1.80)	
Adolescents		4.57 (1.73)	3.91 (1.78)	
Adults		4.84 (1.74)	4.43 (1.76)	
**7-day incidence rate: lockdowns[a]**				
Children	1388.24 (748.67)	26112.44 (6483.02)		
Adolescents	1260.17 (580.60)	26574.33 (6422.74)		
Adults	1519.46 (861.02)	26903.46 (6911.10)		
**Policy stringency: lockdowns[a]**				
Children	2692.19 (338.28)	11423.10 (662.08)		
Adolescents	2685.69 (371.64)	11350.39 (629.22)		
Adults	2706.29 (353.97)	11582.67 (826.81)		
**7-day incidence rate: cumulative[b]**				
Children		16184.48 (6895.33)	36904.24 (8462.62)	37111.06 (8629.51)
Adolescents		17575.76 (6771.14)	37630.71 (8140.34)	37831.16 (8306.19)
Adults		17728.80 (7010.36)	37712.57 (8735.76)	38854.64 (9592.57)
**Policy stringency: cumulative[b]**				
Children		12986.00 (2439.46)	25007.32 (1424.73)	25143.61 (1414.83)
Adolescents		13658.49 (2966.18)	24960.98 (1508.32)	25071.76 (1436.34)
Adults		13466.32 (2798.16)	24946.52 (1624.67)	25276.68 (1653.54)
**7-day incidence rate: recent[c]**				
Children		3601.95 (1734.65)	2550.71 (1311.10)	2463.68 (1249.54)
Adolescents		3586.21 (2056.16)	2580.02 (1263.57)	2457.98 (1181.25)
Adults		4042.60 (1954.53)	2671.59 (1175.15)	2600.83 (1332.66)
**Policy stringency: recent[c]**				
Children		1584.47 (252.06)	1159.28 (206.69)	1163.45 (205.15)
Adolescents		1596.64 (248.14)	1165.61 (210.25)	1168.33 (208.35)

Adults		1640.15 (225.06)	1124.63 (214.66)	1133.68 (212.57)

Note: Perceived threat and burden scores are based on self-reported items on a scale ranging from 1 to 10.
[a]AUC of 7-day incidence rate and policy stringency for the first lockdown (March 1st to 11th of May 2020) and second lockdown (November 2nd 2020 to June 1st 2021).
[b]AUC of the 7-day incidence rate and policy stringency from 1st of March 2020 to Cov2 assessment, Cov3 assessment, and saliva collection for participants' place of residence.
[c]AUC of the 7-day incidence rate or policy stringency 30 days prior the participant's assessment at Cov2, Cov3, and saliva collection for their place of residence.

AUC = area under the curve, M = mean, SD = standard deviation.

Table S5. Descriptive statistics of perceived home chaos and mental well-being

	Children, M (SD)	Adolescents, M (SD)	Adults, M (SD)
Home environment F2F2[a]	2.31 (0.68)	2.14 (0.62)	2.05 (0.64)
Depression F2F3[b]		1.63 (0.46)	1.53 (0.43)
Anxiety F2F3[c]		1.66 (0.62)	1.68 (0.61)
Anxiety Cov2[d]		1.54 (0.64)	1.48 (0.58)
Anxiety Cov3[e]	1.59 (0.63)	1.69 (0.70)	1.59 (0.66)

Note. A higher score on the home chaos scale indicates higher levels of chaos (range 1 to 5). Higher scores on the anxiety (range 1 to 4) and depressive symptoms (range 1 to 4) scales indicate more severe symptoms.
[a]Information missing for 12 children, 25 adolescents, and 169 adults.
[b]Information missing for all children, 45 adolescents, and 7 adults.
[c]Information missing for all children, 26 adolescents, and 3 adults.
[d]Information missing for all children and 1 adolescent.
[e]Information missing for 3 children and 1 adolescent.
M = mean, SD = standard deviation.

**Table S6: Descriptive statistics of raw and residualized epigenetic age based on pre-pandemic and pandemic saliva sampling.**				
	Raw chronological and epigenetic age M (SD)		Epigenetic age residuals M (SD)	
	Pre-pandemic	Pandemic	Pre-pandemic	Pandemic
Chronological age				
All	15.82 (6.03)	18.15 (6.06)		
Children	9.51 (0.32)	11.84 (0.58)		
Adolescents	15.47 (0.31)	17.76 (0.57)		
Adults	24.28 (3.05)	26.63 (3.12)		
Horvath				
All	18.32 (6.54)	20.10 (6.75)	0.01 (0.42)	-0.01 (0.46)
Children	12.65 (2.82)	14.48 (3.48)	-0.01 (0.31)	0.01 (0.39)
Adolescents	18.17 (4.05)	19.98 (4.50)	-0.01 (0.43)	0.01 (0.47)
Adults	25.71 (5.24)	27.38 (5.39)	0.01 (0.48)	-0.01 (0.47)
PedBE				
All	12.50 (1.32)	13.89 (1.59)	-0.17 (0.47)	0.17 (0.53)
Children	11.61 (0.82)	12.85 (1.03)	-0.02 (0.36)	0.02 (0.41)
Adolescents	13.32 (1.15)	14.86 (1.39)	-0.02 (0.42)	0.02 (0.53)
Adults				
GrimAge2				
All	39.50 (5.36)	42.37 (5.70)	-0.09 (0.37)	0.09 (0.39)
Children	35.20 (2.84)	37.57 (3.52)	-0.00 (0.35)	0.00 (0.41)
Adolescents	38.70 (3.17)	42.50 (3.92)	-0.01 (0.34)	0.01 (0.36)
Adults	46.01 (3.69)	48.25 (4.21)	-0.05 (0.35)	0.05 (0.36)
DunedinPACE				
All	1.15 (0.12)	1.20 (0.14)	-0.15 (0.48)	0.15 (0.50)
Children	1.15 (0.11)	1.18 (0.14)	-0.00 (0.46)	0.00 (0.48)
Adolescents	1.13 (0.12)	1.21 (0.15)	-0.03 (0.49)	0.03 (0.52)
Adults	1.17 (0.12)	1.20 (0.15)	-0.10 (0.50)	0.10 (0.51)
Note. “All” for PedBE refers to children and adolescents combined (as explained in section 2.5). Epigenetic age residuals refer to the residuals after regression epigenetic age on chronological age and cell type at both timepoints combined. M = mean, SD = standard deviation.				

4.2 Biological aging during the COVID-19 pandemic.

Table S6 shows means and standard deviations of the raw epigenetic clock estimates and clock estimates residualised for chronological age and cell type. As expected, epigenetic age increases with chronological age, as also illustrated in the scatterplots in Figure S4. To test for changes in the biological aging relative to chronological age, our primary interest lies in the epigenetic age residuals after controlling for chronological age and cell types, which indicate acceleration beyond or below the observed increase in chronological aging in our sample. These residuals are also plotted in Figure 2 of the main text. In Table S7, we summarize the regression results for change in epigenetic aging from pre-pandemic baseline to pandemic follow-up (see section 3.3 for the specific model equations). To exclude viral infection as primary driver of the observed changes in biological aging, we re-ran the same analyses after excluding ever-infected households (see Table S8). The direction of all effects was consistent and remained statistically significant, except for PedBE age acceleration in adolescents (β = 0.14, p = 0.11). Likewise, without batch correction for chip in the pre-processing of DNAm (see section 2.4), effects were consistent in direction and remained statistically significant except for PedBE in adolescents (β = 0.02, p = 0.66).

**Table S7: Change in epigenetic aging during the COVID-19 pandemic**								
	Model 1				Model 2			
	β	SE	p	p.adj	β	SE	p	p.adj
Horvath								
All	-0.01	0.01	0.16	0.64	0.02	0.01	0.03	0.10
Children	0.11	0.06	0.05	0.19	0.14	0.06	0.01	0.06
Adolescents	0.15	0.08	0.04	0.17	0.15	0.07	0.04	0.18
Adults	-0.01	0.02	0.67	1	0.02	0.02	0.46	1.00
PedBE								
All	0.17	0.01	<0.0001	<0.0001	0.14	0.01	<0.0001	<0.0001
Children	0.33	0.07	<0.0001	<0.0001	0.31	0.07	<0.0001	<0.0001
Adolescents	0.24	0.08	0.0045	0.02	0.24	0.08	0.0044	0.02
Adults								
GrimAge2								
All	0.08	0.01	<0.0001	<0.0001	0.05	0.01	<0.0001	<0.0001
Children	-0.01	0.05	0.83	1.00	-0.06	0.04	0.19	0.76
Adolescents	0.19	0.05	0.0003	0.001	0.13	0.05	0.01	0.03
Adults	0.05	0.01	0.0007	0.0029	0.03	0.01	0.06	0.24
DunedinPACE								
All	0.13	0.01	<0.0001	<0.0001	0.1	0.01	<0.0001	<0.0001
Children	0.00	0.08	0.98	1.00	-0.02	0.07	0.74	1.00
Adolescents	0.3	0.08	0.0002	0.0007	0.22	0.08	0.0033	0.01
Adults	0.1	0.02	<0.0001	<0.0001	0.08	0.02	<0.0001	0.0002

Note. Regression results testing change in epigenetic aging from pre-pandemic to pandemic assessment. The statistical parameters refer to the coefficient of time, representing change in epigenetic aging, in the linear mixed models adjusting for genetic relatedness using a GRM in addition to the fixed effects (see section 3.3, M_{eff} = 4).

SE = standard error; p = p-value, p.adj. = adjusted p-value (p-values * M_{eff}), BMI, body-mass-index

Table S8: Change in epigenetic aging during the COVID-19 pandemic after excluding ever-infected households

	Model 1[a]				Model 2[b]			
	β	SE	p	p.adj	β	SE	p	p.adj
Horvath								
All	-0.01	0.01	0.19	0.76	0.02	0.01	0.03	0.11
Children	0.03	0.06	0.58	1.00	0.06	0.06	0.33	1.00
Adolescents	0.03	0.08	0.68	1.00	0.1	0.08	0.21	0.84
Adults	0.00	0.02	0.88	1.00	0.02	0.02	0.38	1.00
PedBE								
All	0.17	0.01	<0.0001	<0.0001	0.14	0.01	<0.0001	<0.0001
Children	0.33	0.07	<0.0001	<0.0001	0.27	0.07	0.0002	0.0007
Adolescents	0.14	0.09	0.11	0.44	0.13	0.09	0.13	0.52
Adults								
GrimAge2								
All	0.08	0.01	<0.0001	<0.0001	0.05	0.01	<0.0001	<0.0001
Children	-0.06	0.05	0.28	1.00	-0.11	0.05	0.03	0.10
Adolescents	0.22	0.05	<0.0001	0.0002	0.14	0.05	0.0042	0.02
Adults	0.05	0.02	0.0019	0.01	0.03	0.02	0.05	0.22
DunedinPACE								
All	0.12	0.01	<0.0001	<0.0001	0.09	0.01	<0.0001	<0.0001
Children	-0.04	0.08	0.59	1.00	-0.09	0.08	0.26	1.00
Adolescents	0.33	0.08	<0.0001	<0.0001	0.27	0.07	0.0002	0.0009
Adults	0.10	0.02	<0.0001	<0.0001	0.09	0.02	<0.0001	0.0002

Note. Regression results testing change in epigenetic aging from pre-pandemic to pandemic assessment after excluding all ever-infected households. Ever-infected households were categorized [yes/no] based on supplementary COVID-19 questionnaires during the third lockdown. The statistical parameters refer to the coefficient of time, representing change in epigenetic aging, in the linear mixed models adjusting for genetic relatedness using a GRM and fixed effects (see section 3.3, M_{eff} = 4).

SE = standard error; p = p-value, p.adj. = adjusted p-value (p-values * M_{eff}), BMI, body-mass-index

4.3 Pandemic stressors and change in biological aging

Table S9 presents all the statistical analysis results of the effects of pandemic stressors on within-individual change in epigenetic age acceleration and pace of aging in Model 1 (controlling for chronological age, cell type, and biological sex) and Model 2 (controlling for chronological age, cell type, biological sex, BMI, and smoking). As described in the main text, only change in PedBE (M1: β = -0.05, p = 0.0003, *p.adj* = 0.0023) and DunedinPACE (M1: β = 0.04, p = 0.0008, *p.adj* = 0.01) was significantly associated with pandemic stressors after multiple testing correction (see Figure S11). After excluding ever-infected households from the analyses, the interaction term for cumulative 7-day incidence rate and PedBE (M1: β = -0.06, p = 0.0002) as well as perceived burden and DunedinPACE (M1: β = 0.03, p = 0.03) remained statistically significant in adolescents. The interaction term of 7-day incidence rate and PedBE also remained significant when accounting for regional clustering by including a random intercept for federal state in the regression model (M1: β = -0.05, p = 0.0003). In addition, we tested whether the effects were robust to the batch correction method. Specifically, since samples ran together on the same chip, we re-ran our analyses excluding chip correction from the pre-preprocessing steps (see section 2.4). Both the interaction of perceived burden and DunedinPACE (M1: β = 0.04, p = 0.0009) and the interaction of 7-day incidence rate and PedBE remained significant (M1: β = -0.023, p = 0.001).

Results of our exploratory analyses of the association of individual burden items with change in the respective clocks are shown in Table S9. For DunedinPACE, interaction terms of time with restriction of civil rights (β = 0.03, p = 0.02) and with separation from important people were nominally significant (β = 0.03, p = 0.03). For PedBE, separation from important people (β = -0.03, p = 0.02) as well as social isolation (β = -0.04, p = 0.004) were nominally significant.

Associations between pandemic stressors and epigenetic age during the pandemic (without accounting for pre-pandemic differences) are presented in Table S12. Also using this simplified analysis approach (see section 3.3 for the respective model equations), we observed the negative association of 7-day incidence rate and PedBE age acceleration (M1: β = -0.11, p = 0.003) as well as the positive association between perceived burden and DunedinPACE (M1: β = 0.09, p = 0.01) in adolescents. In addition, a higher policy stringency in the past month prior to the second saliva sampling was significantly associated with higher Horvath age acceleration in adults (M1: β = 0.11, p = 0.0038, *p.adj = 0.045*). However, in our main analysis, there was no significant interaction term of time x policy stringency after multiple testing correction (M1: β = 0.09, p = 0.01, *p.adj* = 0.09) and we did not observe a significant change in Horvath age acceleration from pre-pandemic baseline (M1: β = -0.01, p = 0.67, *p.adj* = 1.00).

Table S9. Effects of pandemic stressors on within-individual change in epigenetic aging.

		Model 1								Model 2							
		Horvath		PedBE		GrimAge2		DunedinPACE		Horvath		PedBE		GrimAge2		DunedinPACE	
**Predictor**	**Age group**	β	p	β	p	β	p	β	p	β	p	β	p	β	p	β	p
7-day incidence rate (1st lockdown)	All	-0.01	0.38	-0.01	0.38	0.00	0.49	-0.01	0.38	0.00	0.53	0.00	0.83	0.00	0.97	-0.01	0.36
	Children	0.00	0.7	0.01	0.53	0.00	0.95	0.00	0.92	0.01	0.52	0.00	0.68	0.00	0.77	0.00	0.80
	Adolescents	0.00	0.84	-0.03	0.13	-0.02	0.17	-0.02	0.21	-0.01	0.35	-0.01	0.61	0.00	0.89	-0.01	0.68
	Adults	0.00	0.81			0.00	0.73	0.00	0.96	0.00	0.91			0.00	0.62	0.00	0.98
Policy stringency (1st lockdown)	All	0.01	0.14	0.00	0.62	0.01	0.12	0.01	0.47	0.01	0.34	-0.01	0.42	0.01	0.23	0.00	0.65
	Children	0.00	0.83	0.00	0.99	0.01	0.38	0.01	0.58	0.00	0.77	0.00	0.94	0.01	0.42	0.01	0.6
	Adolescents	0.00	0.91	-0.01	0.45	0.00	0.79	0.00	0.83	0.00	0.98	-0.01	0.30	0.00	0.93	0	0.95
	Adults	0.02	0.16			0.01	0.26	-0.01	0.72	0.02	0.12			0.01	0.29	-0.01	0.6
7-day incidence rate (2nd lockdwon)	All	0.00	0.82	-0.02	0.05	0.00	0.82	0.00	0.61	0.00	0.62	-0.01	0.12	0.00	0.64	0.01	0.44
	Children	-0.01	0.5	0	0.89	0.00	0.74	0.00	0.88	-0.01	0.46	0.00	0.91	0.00	0.72	0.00	0.76
	Adolescents	0.00	1	-0.03	0.01	0.01	0.54	0.01	0.47	0.00	0.88	-0.03	0.01	0.01	0.43	0.01	0.33
	Adults	-0.01	0.56			0.00	0.77	0.01	0.55	-0.01	0.53			0.01	0.53	0.01	0.35
Policy stringency (2nd lockdown)	All	0.00	0.5	0.00	0.68	0.00	0.67	0.00	0.88	0.00	0.65	0.01	0.56	0.00	0.91	0.00	0.75
	Children	0.00	0.9	0.00	0.79	-0.01	0.28	-0.01	0.28	0.00	0.84	0.00	0.75	-0.01	0.47	-0.01	0.41
	Adolescents	0.01	0.54	0.00	0.74	-0.01	0.54	0.00	0.96	0.00	0.93	0.00	0.87	0.00	0.74	0.00	0.84
	Adults	-0.01	0.26			0.01	0.17	0.02	0.16	-0.01	0.31			0.01	0.17	0.02	0.13
Threat (2nd lockdown)	All	-0.01	0.61	-0.02	0.10	0.01	0.37	0.00	0.73	0.00	0.93	-0.03	0.02	0.00	0.72	0.00	0.71
	Children	0.00	0.93	0.00	0.81	0.00	0.79	0.01	0.42	0.00	0.98	0.00	0.86	0.00	0.48	0.01	0.23
	Adolescents	-0.01	0.33	-0.02	0.10	0.01	0.21	0.02	0.14	-0.02	0.19	-0.03	0.02	0.01	0.4	0.01	0.62
	Adults	-0.01	0.57			0.00	0.79	-0.01	0.65	-0.01	0.49			0.00	0.96	-0.01	0.70
Burden (2nd lockdown)	All	-0.01	0.43	-0.02	0.13	0.01	0.11	0.00	0.96	0.00	0.76	-0.02	0.20	0.01	0.2	-0.01	0.51
	Children	0.00	0.73	0.00	0.98	-0.01	0.34	0.00	0.72	-0.01	0.48	0.00	0.76	0.00	0.91	0.00	0.70
	Adolescents	0.00	0.85	-0.02	0.13	0.02	0.05	0.01	0.36	0.00	0.88	-0.02	0.20	0.02	0.07	0.01	0.61
	Adults	-0.01	0.41			0.00	0.67	-0.02	0.30	-0.01	0.61			0.00	0.9	-0.02	0.20

Table S9. Effects of pandemic stressors on within-individual change in epigenetic aging.

		Model 1								Model 2							
		Horvath		PedBE		GrimAge2		DunedinPACE		Horvath		PedBE		GrimAge2		DunedinPACE	
**Predictor**	**Age group**	β	p	β	p	β	p	β	p	β	p	β	p	β	p	β	p
Threat (3rd lockdwon)	All	-0.01	0.18	-0.03	0.07	0.00	0.94	-0.01	0.48	-0.01	0.23	-0.02	0.18	0.00	0.67	0.00	0.83
	Children	-0.02	0.09	0.01	0.54	0.01	0.38	0.01	0.26	-0.01	0.21	0.00	0.80	0.00	0.95	0.01	0.61
	Adolescents	-0.02	0.12	-0.03	0.07	0.00	0.75	0.00	0.84	-0.02	0.12	-0.02	0.18	0.00	0.63	0.00	0.77
	Adults	-0.02	0.34			0.01	0.47	0.00	0.87	-0.02	0.31			0.01	0.34	0.01	0.68
Burden (3rd lockdown)	All	-0.02	0.08	-0.03	0.01	0.01	0.19	0.02	0.05	-0.01	0.16	-0.03	0.02	0.01	0.31	0.01	0.16
	Children	-0.02	0.07	0.01	0.39	0.01	0.2	0.00	0.99	-0.02	0.11	0.01	0.51	0.01	0.33	-0.01	0.57
	Adolescents	-0.01	0.35	-0.03	0.01	0.02	0.01	**0.04**	**0.0008**	-0.01	0.29	-0.03	0.02	0.02	0.05	**0.03**	**0.01**
	Adults	-0.02	0.11			0.00	0.78	0.00	0.77	-0.02	0.15			0.00	0.89	-0.01	0.52
7-day incidence rate: recent (3rd lockdown)	All	0.00	0.84	-0.01	0.21	0.01	0.06	0.01	0.27	0.00	0.92	-0.01	0.35	0.01	0.03	0.01	0.35
	Children	-0.01	0.59	0.00	0.67	0.01	0.37	0.01	0.62	-0.01	0.56	0.00	0.66	0.01	0.29	0.01	0.65
	Adolescents	0.00	0.88	-0.03	0.02	0.00	0.76	-0.01	0.56	-0.01	0.41	-0.03	0.04	0.00	0.57	0.00	1.00
	Adults	0.00	0.73			0.02	0.05	0.02	0.2	0.01	0.61			0.02	0.05	0.01	0.25
Policy stringency: recent (3rd lockdwon)	All	0.02	0.02	-0.01	0.10	0.00	0.91	0.00	0.56	0.02	0.02	-0.02	0.09	0.00	0.68	-0.01	0.49
	Children	0.01	0.31	0.00	0.75	-0.01	0.15	-0.01	0.53	0.01	0.42	0.00	0.90	-0.01	0.19	-0.01	0.63
	Adolescents	0.00	0.91	-0.02	0.16	0.00	0.92	0.00	0.73	0.00	0.88	-0.01	0.35	0.00	0.82	0.00	0.94
	Adults	0.04	0.02			0.01	0.54	-0.01	0.55	0.04	0.01			0.00	0.75	-0.01	0.33
7-day incidence rate: cumulative (3rd lockdwon)	All	0.00	0.60	-0.02	0.09	0.00	0.53	0.00	0.63	0.00	0.95	-0.01	0.16	0.01	0.27	0.01	0.36
	Children	0.00	0.80	0.01	0.49	0.00	0.83	0.01	0.58	0.00	0.84	0.01	0.52	0.00	0.99	0.00	0.75
	Adolescents	0.00	0.90	**-0.05**	**0.0003**	0.00	0.65	0.00	0.76	0.00	0.99	**-0.04**	**0.0015**	0.01	0.38	0.01	0.45
	Adults	0.00	0.71			0.00	0.66	0.00	0.78	-0.01	0.62			0.01	0.36	0.01	0.57
Policy stringency:	All	0.01	0.08	0.00	0.93	0.00	0.49	0.00	0.63	0.01	0.08	0.00	1.00	0.00	0.36	-0.01	0.43
	Children	0.01	0.32	0.01	0.32	-0.02	0.05	-0.01	0.41	0.01	0.32	0.01	0.30	-0.02	0.04	-0.01	0.42
	Adolescents	0.00	0.79	-0.01	0.58	-0.01	0.27	-0.01	0.58	0.00	0.81	-0.01	0.69	-0.01	0.35	-0.01	0.70

Table S9. Effects of pandemic stressors on within-individual change in epigenetic aging.

		Model 1								Model 2							
		Horvath		PedBE		GrimAge2		DunedinPACE		Horvath		PedBE		GrimAge2		DunedinPACE	
**Predictor**	**Age group**	**β**	**p**	**β**	**p**	**β**	**p**	**β**	**p**	**β**	**p**	**β**	**p**	**β**	**p**	**β**	**p**
cumulative (3rd lockdown)	Adults	0.03	0.06			0.01	0.17	0.00	0.82	0.03	0.04			0.01	0.19	0.00	0.92

Note. Regression results testing effects of pandemic stressors on change in epigenetic aging from pre-pandemic to pandemic assessment. Statistical parameters are based on the time*stressor interaction term of the linear mixed models adjusting for genetic relatedness using a GRM in addition to the fixed effects (see section 3.3 for specific model equations, Meff = 9). For objective stressors, recent exposure refers to the AUC in the last 30 days prior to the second saliva sampling during the second lockdown. Cumulative exposure refers to the AUC from the start of the COVID-19 pandemic (March 1st, 2020) up to pandemic follow-up.

AUC = Area Under the Curve. SE = standard error; p = p-value.

Table S10. Associations between single burden items and change in epigenetic aging in adolescents

**Predictor**	**Clock**	**β**	**SE**	**p**
DunedinPACE	Restriction of civil rights	0.03	0.01	0.02
	Separation important people	0.03	0.01	0.03
	Lack of leisure activities	0.03	0.01	0.06
	Social isolation	0.02	0.01	0.21
	Unregulated routine	0.00	0.01	0.79
GrimAge2	Restriction of civil rights	0.02	0.01	0.01
	Social isolation	0.01	0.01	0.14
	Lack of leisure activities	0.01	0.01	0.16
	Separation important people	0.01	0.01	0.24
	Unregulated routine	0.00	0.01	0.77
Horvath	Lack of leisure activities	-0.02	0.01	0.11
	Restriction of civil rights	0.01	0.01	0.38
	Separation important people	-0.01	0.01	0.41
	Social isolation	0.00	0.01	0.78
	Unregulated routine	0.00	0.01	0.84
PedBE	Social isolation	-0.04	0.01	0.004
	Separation important people	-0.03	0.01	0.02
	Lack of leisure activities	-0.02	0.01	0.14
	Restriction of civil rights	-0.02	0.01	0.24
	Unregulated routine	-0.01	0.01	0.67

Note. Regression results testing effects of single burden items on change in epigenetic aging from pre-pandemic to pandemic assessment. Statistical parameters are derived time*burden item interaction term of the linear mixed models. Results are ordered by clock and level of significance, from most to least significant per clock.

4.4 Associations of risk factors and epigenetic aging

In addition to pandemic stressors, we explored the effects of several risk factors on epigenetic aging. Tables S11 to S13 show the regression results plotted in Figure 3 of the main text and Figure S11.

Table S11. Associations between risk factors and pre-pandemic epigenetic aging.

		Horvath		PedBE		GrimAge2		DunedinPACE	
Predictor	Age group	β	p	β	p	β	p	β	p
Female [ref = male]	All	0.03	0.35	0.02	0.71	-0.03	0.19	0.09	0.02
	Children	0.04	0.37	0.03	0.52	-0.09	0.02	0.00	0.96
	Adolescents	0.07	0.22	0.00	0.97	-0.06	0.14	0.02	0.77
	Adults	-0.03	0.66			0.08	0.10	0.31	<0.0001
Parental SES	All	0.02	0.30	-0.02	0.35	-0.03	0.03	-0.04	0.03
	Children	0.04	0.07	0.00	0.90	-0.04	0.08	-0.04	0.29
	Adolescents	0.00	0.97	-0.02	0.52	-0.03	0.12	-0.04	0.21
	Adults	0.01	0.77			-0.02	0.33	-0.05	0.19
Personal SES	Adults	0.05	0.48			-0.06	0.2	-0.09	0.21
Parental home [ref = no]	Adults	0.02	0.85			-0.12	0.02	-0.1	0.22
City: 20,000-99,999 [ref = <19,999]	All	-0.03	0.56	-0.02	0.72	-0.06	0.11	-0.12	0.02
	Children	0.15	0.01	0.09	0.20	-0.07	0.17	-0.18	0.03
	Adolescents	-0.08	0.31	-0.14	0.11	0.04	0.45	-0.08	0.37
	Adults	-0.23	0.04			-0.13	0.08	-0.01	0.92
City: 100,000-499,999 [ref = <19,999]	All	0.04	0.42	-0.03	0.60	-0.07	0.09	-0.12	0.04
	Children	-0.01	0.85	0.00	0.99	-0.05	0.38	-0.10	0.32
	Adolescents	0.01	0.95	-0.13	0.17	0.04	0.55	-0.02	0.82
	Adults	0.08	0.48			-0.18	0.02	-0.2	0.07
City: >500,000 [ref = <19,999]	All	0.01	0.85	-0.04	0.49	-0.09	0.02	-0.15	0.01
	Children	0.17	0.01	0.04	0.60	-0.07	0.21	-0.19	0.03
	Adolescents	-0.08	0.38	-0.16	0.08	-0.07	0.26	-0.14	0.12
	Adults	-0.10	0.39			-0.12	0.11	-0.10	0.36
Home chaos	All	0.03	0.08	0.01	0.62	0.02	0.12	0.02	0.43
	Children	0.04	0.06	0.02	0.47	0.00	0.91	0.01	0.83
	Adolescents	0.02	0.50	-0.01	0.82	0.05	0.02	0.04	0.24
	Adults	0.05	0.47			-0.01	0.73	-0.05	0.37
Well-being PGS	All	0.01	0.43	-0.01	0.67	0.01	0.44	0.02	0.25
	Children	0.01	0.58	0.01	0.77	0.00	0.95	-0.01	0.66
	Adolescents	-0.04	0.13	-0.04	0.16	-0.01	0.47	0.00	0.96
	Adults	0.09	0.02			0.03	0.24	0.05	0.19
Anxiety	All	-0.01	0.79	-0.02	0.43	-0.01	0.52	-0.06	0.01
	Adolescents	0.02	0.47	-0.02	0.43	0.00	0.95	-0.04	0.24
	Adults	-0.06	0.14			-0.01	0.63	-0.06	0.11
Depression	All	-0.01	0.62	-0.04	0.25	0.02	0.26	-0.01	0.61
	Adolescents	0.03	0.34	-0.04	0.25	0.00	0.83	-0.03	0.30
	Adults	-0.07	0.07			0.06	0.03	0.02	0.59
Epigenetic Smoking	All	-0.20	<0.0001	0.11	0.0028	0.25	<0.0001	0.3	<0.0001
	Children	-0.18	<0.0001	0.11	0.01	0.24	<0.0001	0.3	<0.0001
	Adolescents	-0.28	<0.0001	0.09	0.09	0.26	<0.0001	0.31	<0.0001
	Adults	-0.18	0.0003			0.21	<0.0001	0.25	<0.0001
Epigenetic BMI	All	0.00	0.92	-0.03	0.2	0.13	<0.0001	0.28	<0.0001
	Children	0.02	0.50	-0.02	0.53	0.06	0.0042	0.26	<0.0001
	Adolescents	-0.05	0.11	-0.06	0.07	0.15	<0.0001	0.29	<0.0001
	Adults	0.05	0.23			0.15	<0.0001	0.26	<0.0001
BMI	All	0.03	0.11	0.03	0.4	0.07	<0.0001	0.14	<0.0001
	Children	-0.03	0.37	0.00	0.94	0.02	0.62	0.06	0.30

	Adolescents	0.01	0.73	0.04	0.32	0.08	0.0023	0.09	0.030
	Adults	0.07	0.07			0.08	0.0015	0.21	<0.0001

Note. Regression results testing effects of risk factors on pre-pandemic epigenetic age acceleration and pace of aging. Statistical parameters are derived from the main effect of the given risk factor on the respective epigenetic clock in the linear mixed models adjusting for genetic relatedness using a GRM in addition to the fixed effects (see section 3.3, Meff = 12).

SES = socioeconomic status, PGS = polygenic score, BMI = body-mass-index, p = p-value.

**Table S12. Associations between risk factors, pandemic stressors, and epigenetic aging at pandemic follow-up.**									
		**Horvath**		**PedBE**		**GrimAge2**		**DunedinPACE**	
**Predictor**	**Age group**	**β**	**p**	**β**	**p**	**β**	**p**	**β**	**p**
Female [ref = male]	All	0.03	0.50	0.00	0.94	0.00	0.91	0.17	<0.0001
	Children	0.07	0.17	0.13	0.03	-0.07	0.11	0.02	0.79
	Adolescents	0.06	0.36	-0.09	0.22	0.02	0.66	0.22	0.0006
	Adults	-0.09	0.23			0.06	0.22	0.29	0.0001
Parental SES	All	0.03	0.08	-0.01	0.62	-0.04	0.0031	-0.08	0.0001
	Children	0.02	0.58	-0.01	0.82	0.00	0.87	-0.03	0.42
	Adolescents	0.03	0.44	-0.03	0.51	-0.07	0.0013	-0.08	0.01
	Adults	0.07	0.06			-0.04	0.11	-0.1	0.0039
Personal SES	Adults	0.04	0.64			-0.13	0.01	-0.15	0.04
Parental home [ref = no]	Adults	-0.04	0.63			0.02	0.75	0.13	0.13
City: 20,000-99,999 [ref = <19,999]	All	-0.10	0.06	-0.17	0.01	-0.07	0.07	-0.08	0.18
	Children	0.03	0.66	-0.07	0.40	0.08	0.21	-0.01	0.95
	Adolescents	-0.17	0.05	-0.23	0.02	-0.16	0.01	-0.13	0.15
	Adults	-0.22	0.06			-0.16	0.04	-0.09	0.41
City: 100,000-499,999 [ref = <19,999]	All	0.03	0.64	-0.06	0.44	-0.07	0.1	-0.07	0.27
	Children	0.06	0.49	0.10	0.29	0.08	0.23	0.09	0.41
	Adolescents	-0.05	0.63	-0.17	0.12	-0.15	0.02	-0.16	0.11
	Adults	0.05	0.62			-0.16	0.04	-0.13	0.24
City: >500,000 [ref = <19,999]	All	-0.01	0.82	-0.17	0.02	-0.04	0.29	-0.07	0.21
	Children	0.02	0.79	0.02	0.84	0.10	0.10	0.07	0.45
	Adolescents	-0.07	0.45	-0.33	0.0022	-0.19	0.0026	-0.24	0.01
	Adults	-0.02	0.88			-0.06	0.43	-0.09	0.42
Home chaos	All	-0.01	0.60	0.03	0.26	0.03	0.05	0.03	0.18
	Children	-0.01	0.76	0.04	0.22	0.05	0.02	0.04	0.18
	Adolescents	0.00	0.96	0.02	0.59	0.01	0.78	0.03	0.4
	Adults	-0.07	0.30			0.05	0.21	0.02	0.71
Well-being PGS	All	0.01	0.51	-0.04	0.06	0.03	0.04	0.05	0.01
	Children	-0.02	0.53	-0.01	0.64	0.03	0.26	0.02	0.45
	Adolescents	-0.01	0.87	-0.07	0.05	0.02	0.46	0.06	0.04
	Adults	0.08	0.04			0.03	0.3	0.05	0.23
Anxiety	All	0.01	0.79	0.02	0.63	0	0.9	-0.02	0.33
	Adolescents	-0.01	0.71	0.02	0.63	0.03	0.15	0.02	0.6
	Adults	0.02	0.67			-0.04	0.09	-0.07	0.06
Depression	All	-0.01	0.69	-0.03	0.43	0.01	0.47	0.02	0.43
	Adolescents	-0.01	0.83	-0.03	0.43	0.04	0.07	0.06	0.08
	Adults	-0.02	0.67			-0.02	0.48	-0.04	0.35
Epigenetic Smoking	All	-0.15	<0.0001	0.18	<0.0001	0.2	<0.0001	0.27	<0.0001
	Children	-0.08	0.03	0.16	0.0003	0.23	<0.0001	0.29	<0.0001
	Adolescents	-0.21	<0.0001	0.19	0.0004	0.19	<0.0001	0.29	<0.0001
	Adults	-0.14	0.0041			0.20	<0.0001	0.22	<0.0001
Epigenetic BMI	All	-0.01	0.48	-0.08	0.0011	0.16	<0.0001	0.31	<0.0001
	Children	0.04	0.17	-0.02	0.5	0.17	<0.0001	0.29	<0.0001
	Adolescents	-0.05	0.16	-0.11	0.0027	0.14	<0.0001	0.3	<0.0001
	Adults	-0.01	0.7			0.18	<0.0001	0.34	<0.0001
BMI	All	0.03	0.19	0.00	0.98	0.06	0.0001	0.17	<0.0001
	Children	0.03	0.48	-0.04	0.48	0.06	0.11	0.20	0.0006
	Adolescents	0.02	0.69	0.03	0.61	0.08	0.0032	0.18	<0.0001
	Adults	0.03	0.39			0.05	0.04	0.15	<0.0001
7-day incidence rate (1st lockdown)	All	-0.01	0.53	-0.01	0.71	-0.01	0.67	-0.02	0.35
	Children	0.01	0.84	0.00	0.95	0.00	0.95	0.01	0.79
	Adolescents	-0.02	0.69	-0.04	0.35	-0.03	0.25	-0.04	0.32
	Adults	-0.01	0.76			0.01	0.81	-0.01	0.7

Table S12. Associations between risk factors, pandemic stressors, and epigenetic aging at pandemic follow-up.

		Horvath		PedBE		GrimAge2		DunedinPACE	
**Predictor**	**Age group**	**β**	**p**	**β**	**p**	**β**	**p**	**β**	**p**
Policy stringency 7-day incidence rate (1st lockdown)	All	0.03	0.06	-0.01	0.7	0.03	0.06	0.02	0.34
	Children	0.02	0.48	0.02	0.6	0.03	0.19	0.03	0.34
	Adolescents	0.00	0.88	-0.02	0.62	0.00	0.93	0.00	0.95
	Adults	0.08	0.03			0.05	0.08	0.00	0.98
7-day incidence rate (2nd lockdown)	All	0.00	0.82	-0.05	0.06	0.01	0.43	0.03	0.2
	Children	-0.02	0.52	-0.01	0.65	0.00	0.94	0.02	0.5
	Adolescents	-0.01	0.77	-0.09	0.02	0.02	0.38	0.03	0.31
	Adults	0.01	0.71			0.01	0.58	0.04	0.28
Policy stringency (2nd lockdown)	All	-0.01	0.64	0.00	0.99	-0.01	0.69	-0.01	0.58
	Children	-0.01	0.85	0.00	0.95	-0.02	0.44	-0.03	0.36
	Adolescents	0.01	0.7	0.00	0.93	-0.02	0.4	-0.02	0.66
	Adults	-0.02	0.44			0.02	0.34	0.02	0.54
Threat (2nd lockdown)	All	0.00	0.87	-0.04	0.27	0.02	0.18	-0.01	0.77
	Children	-0.01	0.6	-0.02	0.54	-0.01	0.53	-0.02	0.6
	Adolescents	0.02	0.64	-0.04	0.27	0.04	0.11	0.02	0.48
	Adults	-0.05	0.23			0.01	0.84	-0.05	0.24
Burden (2nd lockdown)	All	-0.01	0.77	-0.06	0.16	0.02	0.32	0.00	0.87
	Children	0.00	0.91	-0.01	0.79	0.00	0.86	0.01	0.85
	Adolescents	-0.01	0.73	-0.06	0.16	0.02	0.31	0.03	0.47
	Adults	-0.04	0.41			-0.01	0.75	-0.05	0.3
Threat (3rd lockdown)	All	-0.04	0.09	-0.08	0.04	0.02	0.15	0.04	0.11
	Children	-0.04	0.11	-0.01	0.77	0.03	0.21	-0.01	0.85
	Adolescents	-0.04	0.22	-0.08	0.04	0.04	0.05	0.09	0.01
	Adults	-0.08	0.05			0.00	0.92	-0.02	0.62
Burden (3rd lockdown)	All	-0.03	0.21	-0.05	0.20	0.01	0.76	-0.01	0.83
	Children	-0.03	0.34	0.00	0.91	0.02	0.39	0.03	0.32
	Adolescents	-0.04	0.21	-0.05	0.20	0.00	0.98	0.01	0.88
	Adults	-0.04	0.35			0.01	0.74	-0.01	0.80
7-day incidence rate: cumulative (3rd lockdown)	All	0.01	0.67	-0.04	0.08	0.02	0.23	0.03	0.14
	Children	-0.01	0.85	0.01	0.71	0.01	0.7	0.05	0.13
	Adolescents	-0.01	0.76	-0.11	0.0029	0.02	0.47	0.02	0.59
	Adults	0.02	0.66			0.02	0.42	0.03	0.35
7-day incidence rate: recent (3rd lockdown)	All	0.00	0.9	-0.04	0.12	0.03	0.04	0.03	0.18
	Children	-0.01	0.82	0.01	0.86	0.03	0.23	0.03	0.39
	Adolescents	-0.04	0.26	-0.08	0.04	0.00	0.95	-0.02	0.53
	Adults	0.04	0.26			0.06	0.02	0.07	0.06
Policy stringency: cumulative (3rd lockdown)	All	0.04	0.04	-0.01	0.74	-0.01	0.57	-0.01	0.57
	Children	0.03	0.30	0.04	0.17	-0.03	0.22	-0.02	0.61
	Adolescents	0.00	0.90	-0.04	0.26	-0.03	0.15	-0.04	0.2
	Adults	0.09	0.01			0.03	0.13	0.01	0.81
Policy stringency: recent (3rd lockdown)	All	0.04	0.02	-0.04	0.09	0.00	0.74	-0.01	0.62
	Children	0.02	0.39	-0.01	0.64	-0.03	0.2	-0.03	0.31
	Adolescents	0.00	0.96	-0.05	0.20	-0.01	0.69	-0.03	0.40
	Adults	0.11	0.0038			0.02	0.36	0.00	0.92

Note. Regression results testing effects of risk factors on pandemic epigenetic age acceleration and pace of aging. Statistical parameters are derived from the main effect of the given risk factor on the respective epigenetic clock in the linear mixed models, adjusting for genetic relatedness using a GRM in addition to the fixed effects (see section 3.3, Meff = 11). For objective stressors, recent exposure refers to the AUC in the last 30 days prior to the second saliva sampling. Cumulative exposure refers to the AUC from the start of the COVID-19 pandemic (March 1st, 2020) up to the second saliva sampling.

SES = socioeconomic status, PGS = polygenic score, BMI, body-mass-index, AUC = Area Under the Curve, p = p-value.

Table S13. Associations between risk factors and change in epigenetic aging from pre-pandemic baseline to pandemic follow-up.

		Horvath		PedBE		GrimAge2		DunedinPACE	
Predictor	Age group	β	p	β	p	β	p	β	p
Female [ref = male]	All	0.00	0.82	0.00	0.86	0.01	0.39	0.04	0.03
	Children	0.03	0.28	0.05	0.09	0.01	0.72	0.01	0.71
	Adolescents	-0.01	0.76	-0.04	0.21	0.03	0.14	0.09	0.01
	Adults	-0.03	0.41			0.00	0.97	0.02	0.64
Parental SES	All	0.01	0.19	-0.01	0.28	-0.01	0.02	-0.03	0.0004
	Children	0.00	0.89	0.00	0.98	0.00	0.98	-0.01	0.33
	Adolescents	0.01	0.46	-0.01	0.38	-0.03	0.0046	-0.02	0.08
	Adults	0.02	0.09			-0.01	0.22	-0.03	0.02
Personal SES	Adults	0.00	0.98			-0.04	0.03	-0.03	0.22
Parental home [ref = no]	Adults	0.01	0.87			0.03	0.24	0.07	0.05
City: 20,000-99,999 [ref = <19,999]	All	-0.04	0.04	-0.08	0.0028	-0.03	0.10	-0.02	0.46
	Children	0.01	0.79	-0.03	0.27	0.04	0.08	0.02	0.67
	Adolescents	-0.07	0.04	-0.10	0.01	-0.08	0.0015	-0.05	0.19
	Adults	-0.07	0.10			-0.05	0.13	-0.02	0.65
City: 100,000-499,999 [ref = <19,999]	All	-0.01	0.78	-0.03	0.25	-0.02	0.17	-0.02	0.52
	Children	0.02	0.54	0.02	0.59	0.05	0.07	0.05	0.25
	Adolescents	-0.05	0.22	-0.09	0.03	-0.07	0.01	-0.07	0.09
	Adults	0.03	0.53			-0.04	0.18	-0.02	0.6
City: >500,000 [ref = <19,999]	All	-0.01	0.48	-0.07	0.01	-0.01	0.46	-0.01	0.66
	Children	-0.01	0.79	0.01	0.70	0.05	0.04	0.04	0.22
	Adolescents	-0.03	0.42	-0.15	<0.0001	-0.09	0.0007	-0.1	0.01
	Adults	0.00	1.00			-0.01	0.62	-0.03	0.52
Home chaos	All	-0.01	0.35	0.01	0.30	0.01	0.27	0.00	0.59
	Children	-0.01	0.47	0.02	0.12	0.02	0.01	0.02	0.09
	Adolescents	-0.01	0.62	0.01	0.62	0.00	0.84	0.01	0.48
	Adults	-0.03	0.31			0.02	0.16	0.00	0.83
Well-being PGS	All	0.00	0.62	-0.02	0.04	0.01	0.04	0.02	0.0017
	Children	-0.01	0.4	-0.01	0.30	0.01	0.35	0.01	0.58
	Adolescents	0.00	0.7	-0.04	0.0047	0.01	0.26	0.03	0.01
	Adults	0.02	0.28			0.00	0.63	0.01	0.41
Anxiety	All	0.01	0.42	0.00	1.00	0.01	0.18	0.00	0.74
	Adolescents	-0.01	0.42	0.00	1.00	0.02	0.08	0.01	0.32
	Adults	0.01	0.44			-0.01	0.44	-0.02	0.13
Depression	All	0.00	0.86	-0.01	0.27	0.01	0.20	0.01	0.22
	Adolescents	-0.01	0.69	-0.01	0.27	0.02	0.04	0.03	0.03
	Adults	-0.01	0.74			-0.02	0.11	-0.03	0.09
Epigenetic Smoking	All	-0.03	<0.0001	0.05	<0.0001	0.06	<0.0001	0.07	<0.0001
	Children	-0.02	0.05	0.06	<0.0001	0.07	<0.0001	0.07	<0.0001
	Adolescents	-0.05	0.0006	0.03	0.03	0.05	<0.0001	0.06	<0.0001
	Adults	-0.03	0.04			0.07	<0.0001	0.07	<0.0001
Epigenetic BMI	All	0.00	0.85	-0.03	0.0004	0.06	<0.0001	0.11	<0.0001
	Children	0.02	0.03	0.00	0.85	0.07	<0.0001	0.11	<0.0001
	Adolescents	-0.01	0.5	-0.05	0.0002	0.05	<0.0001	0.11	<0.0001
	Adults	-0.01	0.5			0.06	<0.0001	0.12	<0.0001
BMI	All	0.00	0.71	0.01	0.48	0.01	0.02	0.04	<0.0001
	Children	0.02	0.17	-0.01	0.62	0.02	0.16	0.08	0.0006
	Adolescents	0.01	0.73	0.00	0.85	0.03	0.01	0.07	<0.0001
	Adults	0.00	0.81			0.01	0.48	0.04	0.0038

Note. Regression results testing effects of risk factors on change in epigenetic aging from pre-pandemic to pandemic assessment. Statistical parameters are based on the time*risk interaction term of the linear mixed models adjusting for genetic relatedness using a GRM in addition to the fixed effects (see section 3.3, Meff = 11).

SES = socioeconomic status, PGS = polygenic score, BMI, body-mass-index, p = p-value.

4.5 Associations of risk factors and objective stressors with subjective well-being and stressors

Figure S11 illustrates the regression results of all tested risk factors and objective stressors with pre-pandemic and pandemic well-being and subjective stressors during the second and third lockdown (see section 3.3 for model equations). Pre-pandemic anxiety was significantly positively associated with perceived threat (second lockdown: r = 0.20; at third lockdown: r = 0.22) and burden (second lockdown: r = 0.21; third lockdown: r = 0.28) at both assessments during the pandemic but did not increase from pre-pandemic baseline (adolescents: β = 0.01, p = 0.53; adults: β = -0.48, p = 0.03).

Several of the risk factors (i.e., sex, parental SES, WBS PGS, anxiety, depressive symptoms, and home chaos) significantly predicted perceived threat and burden, or anxiety in at least one age group. The direction of these effects was consistent across the developmental phases and, when available, before and during the pandemic. For example, a higher genetic predisposition for well-being was associated with lower levels of anxiety before and during the COVID-19 pandemic, as well as lower pandemic threat during the second lockdown and burden during the second and third lockdown. City size, on the other hand, affected only the perception of pandemic-related threat and burden, but not anxiety. In contrast, objective pandemic stressors were only nominally significantly associated with higher levels of perceived threat and burden in adults and not in any other age group. With anxiety, we observed two significant associations during the third lockdown. Specifically, policy stringency during the first lockdown was negatively associated with anxiety in children during the third lockdown (β = -0.15, p = 0.0043, $p.adj$ = 0.03), whereas policy stringencyduring the second lockdown were positively associated with anxiety during the third lockdown in the full sample (β = 0.14, p = 0.002, $p.adj$ = 0.02).

In summary, psychological stress levels during the pandemic were mostly associated with pre-pandemic factors, and less associated with objective measures of pandemic-related restrictions. All regression results of these analyses are presented in Table S14 to S16.

Table S14. Associations of risk factors with pre-pandemic anxiety and depression at pre-pandemic baseline

		Depression		Anxiety	
**Predictor**	**Age group**	**β**	**p**	**β**	**p**
Female [ref = male]	All	0.25	0.01	0.38	<0.0001
	Adolescents	0.37	0.0018	0.47	<0.0001
	Adults	0.09	0.5	0.25	0.06
Parental SES	All	-0.13	0.0024	-0.05	0.25
	Adolescents	-0.04	0.59	0.00	0.99
	Adults	-0.2	0.0005	-0.12	0.04
Personal SES	Adults	-0.12	0.22	0.03	0.76
Parental home [ref = no]	Adults	0.24	0.06	0.08	0.53
City: 20,000-99,999 [ref = <19,999]	All	-0.01	0.97	-0.05	0.71
	Adolescents	0.04	0.79	-0.03	0.85
	Adults	-0.03	0.86	-0.12	0.52
City: 100,000-499,999 [ref = <19,999]	All	-0.01	0.95	0	0.97
	Adolescents	0.15	0.41	0.12	0.51
	Adults	-0.12	0.51	-0.19	0.29
City: > 500,000 [ref = <19,999]	All	0.01	0.96	-0.03	0.84
	Adolescents	0.10	0.56	-0.09	0.58
	Adults	-0.10	0.6	-0.01	0.97
Home chaos	All	0.15	0.0035	0.07	0.15
	Adolescents	0.15	0.01	0.08	0.17
	Adults	0.18	0.05	0.07	0.51
Well-being PGS	All	-0.08	0.08	-0.1	0.02
	Adolescents	0.04	0.48	0.00	0.98
	Adults	-0.21	0.0007	-0.23	0.0002
Anxiety	All	0.60	<0.0001		
	Adolescents	0.70	<0.0001		
	Adults	0.52	<0.0001		
Depression	All			0.57	<0.0001
	Adolescents			0.61	<0.0001
	Adults			0.55	<0.0001
Epigenetic Smoking	All	0.08	0.04	0.04	0.29
	Adolescents	0.01	0.89	-0.03	0.63
	Adults	0.15	0.0037	0.10	0.07
Epigenetic BMI	All	0.04	0.31	0.03	0.41
	Adolescents	0.04	0.50	0.04	0.45
	Adults	0.06	0.34	0.04	0.54
BMI	All	0.15	0.0014	0.07	0.15
	Adolescents	0.17	0.03	0.13	0.08
	Adults	0.16	0.01	0.01	0.88

Note. Regression results testing effects of risk factors on pre-pandemic anxiety and depression. Statistical parameters are derived from the main effect of the given risk factor on the respective outcome in the linear mixed models, adjusting for genetic relatedness using a GRM in addition to the fixed effects (see section 3.3, Meff = 8). Please note that anxiety and depressive symptoms were not measured in children at this timepoint.

SES = socioeconomic status, PGS = polygenic score, BMI, body-mass-index, p = p-value

Table S15. Associations of risk factors and objective stressors with anxiety and subjective stressors during the second lockdown.

		Anxiety		Burden		Threat	
**Predictor**	**Age group**	**β**	**p**	**β**	**p**	**β**	**p**
Female [ref = male]	All	0.5	<0.0001	0.26	0.0023	0.33	<0.0001
	Children			-0.01	0.91	0.02	0.88
	Adolescents	0.66	<0.0001	0.33	0.0028	0.46	<0.0001
	Adults	0.27	0.04	0.17	0.22	0.15	0.17
Parental SES	All	-0.07	0.09	-0.06	0.18	-0.09	0.01
	Children			0.10	0.15	-0.08	0.29
	Adolescents	-0.04	0.54	-0.09	0.13	-0.18	0.0005
	Adults	-0.09	0.10	-0.04	0.49	-0.01	0.8
Personal SES	Adults	-0.01	0.90	-0.13	0.26	-0.11	0.22
Parental home [ref = no]	Adults	0.17	0.17	-0.09	0.50	0.18	0.10
City: 20,000-99,999 [ref = <19,999]	All	0.08	0.49	-0.18	0.14	-0.03	0.77
	Children			-0.34	0.04	-0.39	0.03
	Adolescents	0.12	0.45	-0.15	0.34	0.01	0.94
	Adults	0.00	1.00	-0.25	0.18	-0.12	0.43
City: 100,000-499,999 [ref = <19,999]	All	0.01	0.93	-0.27	0.03	-0.29	0.01
	Children			-0.3	0.12	-0.58	0.004
	Adolescents	-0.10	0.57	-0.23	0.17	-0.27	0.08
	Adults	0.11	0.53	-0.36	0.04	-0.34	0.02
City: >500,000 [ref = <19,999]	All	0.12	0.32	-0.19	0.13	-0.16	0.12
	Children			-0.11	0.52	-0.21	0.24
	Adolescents	0.12	0.50	-0.15	0.38	-0.2	0.16
	Adults	0.10	0.58	-0.27	0.14	-0.13	0.39
Home environment	All	0.12	0.01	0.08	0.10	0.09	0.03
	Children			0.01	0.79	0.12	0.04
	Adolescents	0.08	0.17	0.08	0.12	0.17	0.0009
	Adults	0.22	0.01	0.05	0.59	-0.1	0.2
Well-being PGS	All	-0.12	0.0041	-0.07	0.09	-0.07	0.05
	Children			-0.06	0.31	-0.14	0.03
	Adolescents	-0.06	0.24	0.02	0.67	0.00	0.93
	Adults	-0.19	0.0019	-0.22	0.0006	-0.17	0.0007
Anxiety	All	0.26	<0.0001	0.09	0.02	0.03	0.33
	Adolescents	0.21	<0.0001	0.08	0.09	0.01	0.92
	Adults	0.35	<0.0001	0.09	0.14	0.06	0.23
Depression	All	0.28	<0.0001	0.06	0.12	0.07	0.04
	Adolescents	0.22	<0.0001	0.05	0.27	0.03	0.57
	Adults	0.38	<0.0001	0.09	0.15	0.14	0.01
Epigenetic Smoking	All	0.05	0.21	-0.03	0.43	-0.04	0.22
	Children			-0.01	0.85	0.00	0.97
	Adolescents	-0.01	0.87	-0.08	0.11	-0.06	0.18
	Adults	0.1	0.05	0.02	0.75	-0.02	0.63
Epigenetic BMI	All	0.00	0.92	-0.02	0.56	0.02	0.57
	Children			-0.05	0.35	-0.09	0.17
	Adolescents	0.04	0.44	0.02	0.66	0.03	0.51
	Adults	-0.03	0.60	-0.07	0.22	0.04	0.46
BMI	All	0.09	0.05	0.02	0.7	0.07	0.08
	Children			-0.09	0.42	-0.14	0.24

Table S15. Associations of risk factors and objective stressors with anxiety and subjective stressors during the second lockdown.

		Anxiety		Burden		Threat	
**Predictor**	**Age group**	**β**	**p**	**β**	**p**	**β**	**p**
	Adolescents	0.09	0.20	0.13	0.06	0.21	0.0012
	Adults	0.10	0.09	-0.08	0.19	-0.03	0.57
7-day incidence rate (1st lockdown)	All	0.00	0.96	-0.04	0.34	-0.01	0.79
	Children			-0.04	0.53	-0.11	0.09
	Adolescents	-0.01	0.93	-0.07	0.29	-0.04	0.51
	Adults	0.00	0.98	-0.02	0.66	0.01	0.8
Policy stringency (1st lockdown)	All	-0.02	0.71	0.00	0.94	-0.02	0.57
	Children			0.01	0.85	-0.11	0.09
	Adolescents	0.01	0.85	0.06	0.22	-0.03	0.57
	Adults	-0.06	0.35	-0.09	0.14	-0.02	0.63
7-day incidence rate: cumulative (2nd lockdown)	All	0.09	0.03	0.05	0.27	-0.03	0.39
	Children			0.01	0.86	-0.05	0.43
	Adolescents	0.04	0.53	0.00	0.99	-0.07	0.16
	Adults	0.15	0.01	0.1	0.1	0.02	0.64
7-day incidence rate: recent (2nd lockdown)	All	-0.01	0.87	-0.03	0.53	0.03	0.34
	Children			0.05	0.48	0.02	0.74
	Adolescents	0.00	0.93	0.00	0.94	0.05	0.29
	Adults	-0.03	0.63	-0.08	0.22	-0.01	0.84
Policy stringency: cumulative (2nd lockdown)	All	0.05	0.25	0.07	0.07	-0.04	0.3
	Children			-0.04	0.57	0.02	0.82
	Adolescents	0.00	0.99	0.04	0.43	-0.07	0.12
	Adults	0.10	0.08	0.12	0.05	0.03	0.58
Policy stringency: recent (2nd lockdown)	All	0.06	0.13	0.05	0.23	-0.02	0.62
	Children			-0.01	0.81	0.03	0.62
	Adolescents	0.02	0.74	-0.03	0.61	-0.04	0.46
	Adults	0.15	0.03	0.17	0.01	0.02	0.78

Note. Regression results testing effects of risk factors on anxiety, and perceived threat and burden during the second lockdown. Statistical parameters are derived from the main effect of the given risk factor or objective stressor on the respective outcome in the linear mixed models, adjusting for genetic relatedness using a GRM in addition to the fixed effects (see section 3.3, Meff = 8). For objective stressors, recent exposure refers to the AUC in the last 30 days prior to date of the self-reported symptoms during the second lockdown. Cumulative exposure refers to the AUC from the start of the COVID-19 pandemic (March 1st, 2020) up to date of the self-reported symptoms during the second lockdown. Please note that anxiety symptoms were not assessed in children at this timepoint.

SES = socioeconomic status, PGS = polygenic score, BMI, body-mass-index, AUC = Area Under the Curve; p = p-value.

Table S16. Associations of risk factors and objective stressors with anxiety and subjective stressors during the third lockdown.

		Anxiety		Burden		Threat	
**Predictor**	**Age group**	**β**	**p**	**β**	**p**	**β**	**p**
Female [ref = male]	All	0.36	<0.0001	0.22	0.02	0.18	0.03
	Children	0.26	0.01	-0.18	0.15	-0.19	0.12
	Adolescents	0.57	<0.0001	0.43	0.0001	0.33	0.0023
	Adults	0.04	0.76	-0.09	0.51	-0.03	0.80
Parental SES	All	-0.03	0.51	-0.02	0.61	0.03	0.46
	Children	-0.05	0.41	-0.01	0.84	-0.04	0.55
	Adolescents	0.05	0.41	-0.07	0.24	-0.04	0.54
	Adults	-0.11	0.08	0.00	1.00	0.08	0.15
Personal SES	Adults	-0.05	0.62	-0.17	0.11	0.10	0.37
Parental home [ref = no]	Adults	0.20	0.14	0.05	0.66	0.16	0.20
City: 20,000-99,999 [ref = <19,999]	All	0.04	0.77	-0.2	0.11	-0.17	0.16
	Children	-0.07	0.63	-0.18	0.28	-0.56	0.0006
	Adolescents	0.13	0.45	-0.08	0.62	-0.06	0.72
	Adults	-0.13	0.48	-0.43	0.02	-0.36	0.04
City: 100,000-499,999 [ref = <19,999]	All	-0.03	0.79	-0.15	0.24	-0.18	0.14
	Children	-0.26	0.12	-0.38	0.05	-0.46	0.01
	Adolescents	-0.05	0.79	-0.14	0.42	-0.14	0.41
	Adults	-0.07	0.72	-0.23	0.2	-0.28	0.1
City: >500,000 [ref = <19,999]	All	-0.03	0.83	-0.09	0.5	-0.16	0.17
	Children	-0.28	0.06	-0.05	0.75	-0.39	0.02
	Adolescents	-0.02	0.92	-0.08	0.66	-0.12	0.48
	Adults	-0.09	0.63	-0.17	0.34	-0.25	0.14
Home chaos	All	0.05	0.36	0.01	0.77	0.04	0.38
	Children	0.02	0.74	0.06	0.23	0.06	0.21
	Adolescents	0.01	0.83	-0.06	0.28	0.01	0.85
	Adults	0.17	0.11	0.27	0.002	0.16	0.06
Well-being PGS	All	-0.09	0.03	-0.08	0.07	-0.02	0.54
	Children	-0.15	0.0044	-0.11	0.07	-0.09	0.12
	Adolescents	-0.05	0.35	-0.03	0.63	0.02	0.71
	Adults	-0.15	0.02	-0.15	0.02	-0.10	0.11
Anxiety	All	0.34	<0.0001	0.06	0.08	0.08	0.03
	Adolescents	0.34	<0.0001	0.03	0.59	0.03	0.57
	Adults	0.37	<0.0001	0.10	0.07	0.13	0.02
Depression	All	0.36	<0.0001	0.09	0.01	0.04	0.3
	Adolescents	0.31	<0.0001	0.08	0.10	0.02	0.75
	Adults	0.46	<0.0001	0.10	0.07	0.08	0.15
Epigenetic Smoking	All	0.06	0.12	0.03	0.43	0.00	0.92
	Children	-0.02	0.75	0.03	0.58	0.12	0.02
	Adolescents	0.05	0.32	0.02	0.68	0.00	0.91
	Adults	0.07	0.2	0.04	0.40	0.00	0.97
Epigenetic BMI	All	-0.02	0.61	0.03	0.40	-0.01	0.88
	Children	0.06	0.27	0.07	0.16	0.00	0.94
	Adolescents	0.01	0.91	0.09	0.06	0.00	0.96
	Adults	-0.05	0.44	-0.02	0.70	0.00	0.95
BMI	All	-0.01	0.86	0.02	0.75	0.02	0.61
	Children	0.04	0.67	0.08	0.45	-0.15	0.14
	Adolescents	-0.01	0.87	0.09	0.21	0.10	0.16
	Adults	-0.01	0.92	-0.05	0.44	-0.04	0.46
7-day incidence rate (1st lockdown)	All	0.01	0.74	-0.03	0.47	0.00	0.95
	Children	-0.07	0.19	-0.01	0.87	-0.08	0.22
	Adolescents	0.05	0.5	-0.05	0.51	-0.03	0.70
	Adults	0.00	0.94	-0.02	0.68	0.01	0.84
	All	-0.02	0.68	0.06	0.13	0.07	0.07

Table S16. Associations of risk factors and objective stressors with anxiety and subjective stressors during the third lockdown.

		Anxiety		Burden		Threat	
**Predictor**	**Age group**	**β**	**p**	**β**	**p**	**β**	**p**
Policy stringency (1st lockdown)	Children	-0.15	0.0043	-0.08	0.21	-0.04	0.55
	Adolescents	0.02	0.77	0.09	0.09	0.05	0.38
	Adults	-0.06	0.35	0.01	0.84	0.10	0.08
7-day incidence rate: cumulative (2nd lockdown)	All	0.10	0.03	-0.01	0.8	0.00	0.99
	Children	-0.03	0.55	0.07	0.29	-0.09	0.15
	Adolescents	0.03	0.66	-0.03	0.57	-0.07	0.21
	Adults	0.16	0.01	0.01	0.92	0.08	0.19
7-day incidence rate: recent (2nd lockdown)	All	0.01	0.82	0.02	0.59	0.00	0.93
	Children	-0.01	0.88	-0.01	0.84	0.08	0.24
	Adolescents	0.01	0.89	0.02	0.67	-0.01	0.89
	Adults	0.02	0.8	0.00	0.98	-0.01	0.91
Policy stringency: cumulative (2nd lockdown)	All	0.05	0.21	-0.06	0.12	-0.03	0.48
	Children	-0.07	0.23	0.04	0.61	-0.09	0.20
	Adolescents	0.00	0.98	-0.07	0.19	-0.09	0.09
	Adults	0.11	0.1	-0.05	0.4	0.06	0.32
Policy stringency: recent (2nd lockdown)	All	0.14	0.0017	-0.04	0.32	0.03	0.46
	Children	-0.09	0.1	-0.01	0.84	-0.09	0.14
	Adolescents	0.12	0.03	-0.04	0.47	0.03	0.64
	Adults	0.16	0.02	-0.05	0.43	0.03	0.63
7-day incidence rate: cumulative (3rd lockdown)	All	0.08	0.08	0.02	0.7	0.01	0.73
	Children	-0.01	0.88	0.05	0.45	-0.08	0.18
	Adolescents	0.06	0.28	0.00	0.98	0.00	0.99
	Adults	0.09	0.16	0.04	0.52	0.04	0.51
7-day incidence rate: recent (3rd lockdown)	All	0.07	0.1	0.03	0.45	0.02	0.55
	Children	-0.09	0.06	0.05	0.35	0.02	0.71
	Adolescents	0.08	0.15	0.00	0.99	-0.01	0.79
	Adults	0.03	0.62	0.08	0.17	0.08	0.19
Policy stringency: cumulative (3rd lockdown)	All	-0.03	0.43	-0.01	0.84	0.02	0.54
	Children	-0.06	0.26	-0.02	0.77	0.05	0.4
	Adolescents	-0.03	0.63	-0.02	0.74	-0.02	0.75
	Adults	-0.02	0.71	0.03	0.64	0.09	0.07
Policy stringency: recent (3rd lockdown)	All	-0.05	0.28	0.03	0.45	0.01	0.8
	Children	0.09	0.11	0.08	0.2	0.11	0.06
	Adolescents	-0.04	0.48	0.03	0.64	0.03	0.53
	Adults	-0.04	0.48	0.05	0.44	-0.01	0.87

Note. Regression results testing effects of risk factors on anxiety, and perceived threat and burden during the third lockdown. Statistical parameters are derived from the main effect of the given risk factor or objective stressor on the respective outcome in the linear mixed models, adjusting for genetic relatedness using a GRM in addition to the fixed effects (see section 3.3, Meff = 8). For objective stressors, recent exposure refers to the AUC in the last 30 days prior to date of the self-reported symptoms during the second lockdown. Cumulative exposure refers to the AUC from the start of the COVID-19 pandemic (March 1st, 2020) up to date of the self-reported symptoms during the second lockdown.

SES = socioeconomic status, PGS = polygenic score, BMI, body-mass-index, AUC = Area Under the Curve; p = p-value.

4.6 Figure S11. Heatmap of all associations of objective pandemic stressors and individual risk factors with epigenetic aging and pandemic perception

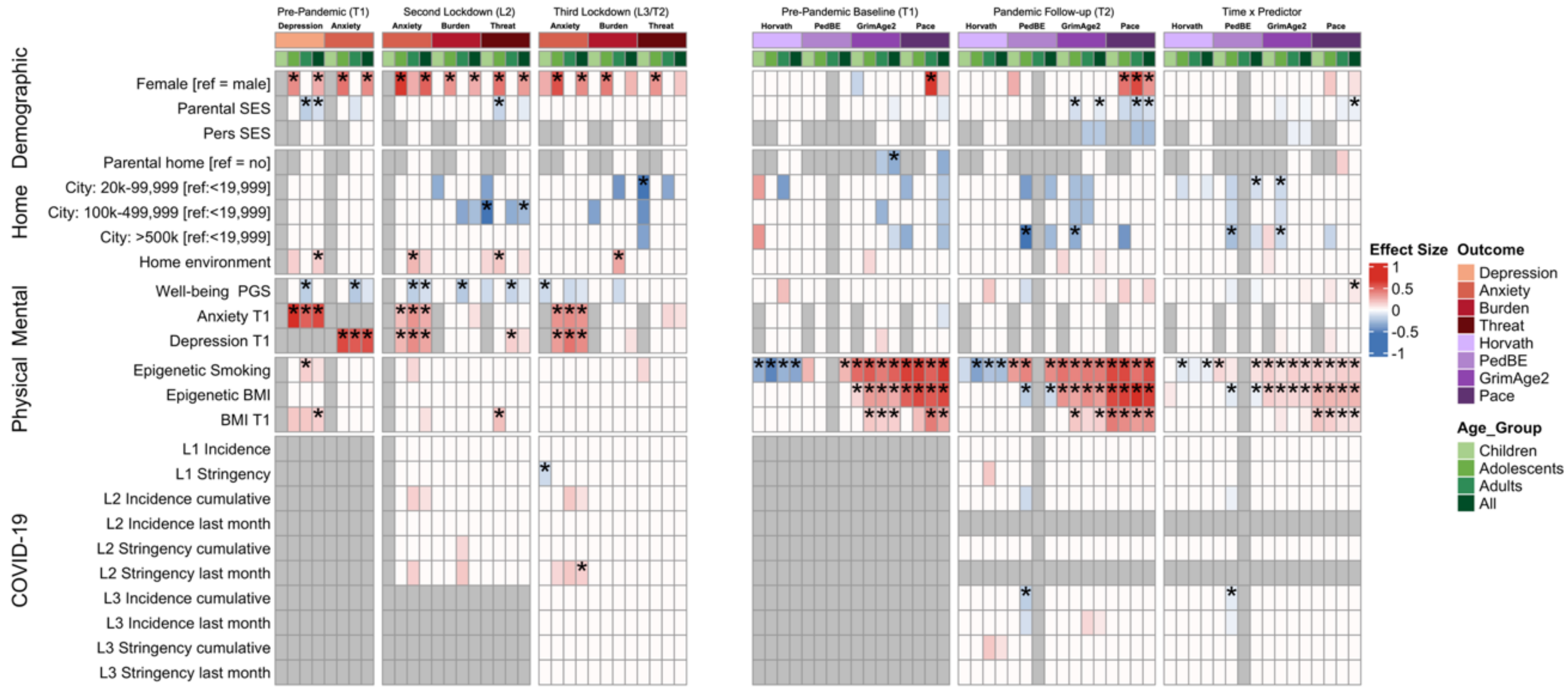

The heatmap shows all nominally significant positive (coloured in red) or negative (coloured in blue) associations of demographic, home environment, and mental and physical health-related vulnerability factors as well as objective stressors with perceived pandemic stress, anxiety, and depression (left) as well as epigenetic age acceleration and pace of aging (right). Effect size of the standardized beta coefficients is illustrated by colour intensity. Non-significant associations are left blank. Not tested associations (due to missingness or timing of the occurrence of the given predictor) are coloured in grey. For the self-reported outcomes, objective stressors for the first lockdown are defined as the AUC from March 1st to 11th of May 2020. For the second and third lockdown, we computed cumulative (starting from the 1st of March 2020, "cumulative") and recent (starting 30 days prior to the Cov3 self-reports, "last month") exposure up to the date of the self-report assessment using the AUC. For most participants, the Cov3 self-report during the third lockdown coincided with the saliva sampling (see SI Appendix, section 4.1). For the epigenetic outcomes, objective stressors are defined as follows: For the first and second lockdown, exposure to 7-day incidence rate and policy stringency are summarized as the AUC from March 1st to 11th of May 2020 and from November 2nd 2020 to June 1st 2021, respectively. For the third lockdown, we computed cumulative (starting from the 1st of March 2020, "cumulative") and recent (starting 30 days prior to the

saliva collection, “last month”) exposure up to the date of the second (pandemic) saliva collection using the AUC. The corresponding statistics of these results are provided in the SI Appendix, section 4.4 and 4.5 (Table S10 to S15). AUC = area under the curve, SES = socioeconomic status, PGS = polygenic score, BMI = body-mass-index, L1 = first lockdown, L2 = second lockdown, L3 = third lockdown. *$p < 0.05$ after multiple testing correction (self-reports: $M_{eff} = 8$, epigenetic clocks: $M_{eff} = 11$).

5. References

1. B. Mönkediek, *et al.*, The German Twin Family Panel TwinLife: Current State and (Epi-)genetically Informative Satellite Projects. *Twin Res. Hum. Genet. Off. J. Int. Soc. Twin Stud.* 1–16 (2026). https://doi.org/10.1017/thg.2026.10064.

2. L. Frach, *et al.*, (Epi-)Genomic Data in the German TwinLife Study: TwinSNPs and TECS Cohort Profiles. [Preprint] (2026). Available at: https://www.biorxiv.org/content/10.64898/2026.02.20.704007v1 [Accessed 24 February 2026].

3. T. Rohm, *et al.*, Data from the German TwinLife Study: Genetic and Social Origins of Educational Predictors, Processes, and Outcomes. **11**, 4 (2023).

4. M. Deppe, *et al.*, Personality and well-being are genetically associated: Extending previous twin and molecular genetic studies. *Eur. J. Personal.* **40**, 935–952 (2026).

5. T. Ge, C.-Y. Chen, Y. Ni, Y.-C. A. Feng, J. W. Smoller, Polygenic prediction via Bayesian regression and continuous shrinkage priors. *Nat. Commun.* **10**, 1776 (2019).

6. S. Purcell, *et al.*, PLINK: A Tool Set for Whole-Genome Association and Population-Based Linkage Analyses. *Am. J. Hum. Genet.* **81**, 559–575 (2007).

7. M. Deppe, *et al.*, Personality and well-being are genetically associated: Extending previous twin and molecular genetic studies. [Preprint] (2024). Available at: https://osf.io/6ctm4 [Accessed 21 January 2025].

8. J. Yang, S. H. Lee, M. E. Goddard, P. M. Visscher, GCTA: A Tool for Genome-wide Complex Trait Analysis. *Am. J. Hum. Genet.* **88**, 76–82 (2011).

9. L. Sinke, D. Cats, B. T. Heijmans, Omixer: multivariate and reproducible sample randomization to proactively counter batch effects in omics studies. *Bioinformatics* **37**, 3051–3052 (2021).

10. J. Maksimovic, B. Phipson, A. Oshlack, A cross-package Bioconductor workflow for analysing methylation array data. *F1000Research* **5**, 1281 (2016).

11. M. J. Aryee, *et al.*, Minfi: a flexible and comprehensive Bioconductor package for the analysis of Infinium DNA methylation microarrays. *Bioinforma. Oxf. Engl.* **30**, 1363–1369 (2014).

12. N. Touleimat, J. Tost, Complete pipeline for Infinium(®) Human Methylation 450K BeadChip data processing using subset quantile normalization for accurate DNA methylation estimation. *Epigenomics* **4**, 325–341 (2012).

13. A. E. Teschendorff, *et al.*, A beta-mixture quantile normalization method for correcting probe design bias in Illumina Infinium 450 k DNA methylation data. *Bioinforma. Oxf. Engl.* **29**, 189–196 (2013).

14. Y. Chen, *et al.*, Discovery of cross-reactive probes and polymorphic CpGs in the Illumina Infinium HumanMethylation450 microarray. *Epigenetics* **8**, 203–209 (2013).

15. D. L. McCartney, *et al.*, Identification of polymorphic and off-target probe binding sites on the Illumina Infinium MethylationEPIC BeadChip. *Genomics Data* **9**, 22–24 (2016).

16. J. T. Leek, W. E. Johnson, H. S. Parker, A. E. Jaffe, J. D. Storey, The sva package for removing batch effects and other unwanted variation in high-throughput experiments. *Bioinforma. Oxf. Engl.* **28**, 882–883 (2012).

17. H.-J. Westra, *et al.*, MixupMapper: correcting sample mix-ups in genome-wide datasets increases power to detect small genetic effects. *Bioinforma. Oxf. Engl.* **27**, 2104–2111 (2011).

18. S. Horvath, DNA methylation age of human tissues and cell types. *Genome Biol.* **14**, R115 (2013).

19. L. M. McEwen, *et al.*, The PedBE clock accurately estimates DNA methylation age in pediatric buccal cells. *Proc. Natl. Acad. Sci.* **117**, 23329–23335 (2020).

20. A. T. Lu, *et al.*, DNA methylation GrimAge version 2. *Aging* **14**, 9484–9549 (2022).

21. D. W. Belsky, *et al.*, DunedinPACE, a DNA methylation biomarker of the pace of aging. *ELIFE* **11**, e73420 (2022).

22. R. J. Musci, *et al.*, Using Epigenetic Clocks to Characterize Biological Aging in Studies of Children and Childhood Exposures: a Systematic Review. *Prev. Sci.* **24**, 1398–1423 (2023).

23. J. Ryan, J. Wrigglesworth, J. Loong, P. D. Fransquet, R. L. Woods, A Systematic Review and Meta-analysis of Environmental, Lifestyle, and Health Factors Associated With DNA Methylation Age. *J. Gerontol. Ser. -Biol. Sci. Med. Sci.* **75**, 481–494 (2020).

24. F. Dammering, *et al.*, The pediatric buccal epigenetic clock identifies significant ageing acceleration in children with internalizing disorder and maltreatment exposure. *Neurobiol. Stress* **15**, 100394 (2021).

25. C. McCrory, *et al.*, GrimAge Outperforms Other Epigenetic Clocks in the Prediction of Age-Related Clinical Phenotypes and All-Cause Mortality. *J. Gerontol. Ser. A* **76**, 741–749 (2021).

26. A. T. Lu, *et al.*, DNA methylation GrimAge strongly predicts lifespan and healthspan. *Aging* **11**, 303–327 (2019).

27. J. D. Faul, *et al.*, Epigenetic-based age acceleration in a representative sample of older Americans: Associations with aging-related morbidity and mortality. *Proc. Natl. Acad. Sci.* **120**, e2215840120 (2023).

28. A. J. deSteiguer, *et al.*, Stability of Aging- and Cognition-Related Methylation Profile Scores Across Two Waves in Children and Adolescents. *Child Dev.* **96**, 1189–1206 (2025).

29. L. Raffington, *et al.*, Associations of socioeconomic disparities with buccal DNA-methylation measures of biological aging. *Clin. Epigenetics* **15**, 70 (2023).

30. L. Raffington, *et al.*, Socially Stratified Epigenetic Profiles Are Associated With Cognitive Functioning in Children and Adolescents. *Psychol. Sci.* **34**, 170–185 (2023).

31. N. C. dos S. Oliveira, S. Katrinli, S. G. de Assis, A. K. Smith, F. Serpeloni, Community and domestic violence are associated with DNA methylation GrimAge acceleration and heart rate variability in adolescents. *Eur. J. PSYCHOTRAUMATOLOGY* **14**, 2202054 (2023).

32. D. W. Belsky, *et al.*, DunedinPACE, a DNA methylation biomarker of the pace of aging. *eLife* **11**, e73420 (2022).

33. D. W. Belsky, *et al.*, Quantification of the pace of biological aging in humans through a blood test, the DunedinPoAm DNA methylation algorithm. *eLife* **9**, e54870 (2020).

34. D. Pelegí-Sisó, P. de Prado, J. Ronkainen, M. Bustamante, J. R. González, methylclock: a Bioconductor package to estimate DNA methylation age. *Bioinformatics* **37**, 1759–1760 (2021).

35. S. Horvath, DNA methylation age of human tissues and cell types. *Genome Biol.* **14**, 3156 (2013).

36. J. Schilling, *et al.*, Die verschiedenen Phasen der COVID-19-Pandemie in Deutschland: Eine deskriptive Analyse von Januar 2020 bis Februar 2021. *Bundesgesundheitsblatt Gesundheitsforschung Gesundheitsschutz* **64**, 1093 (2021).

37. J. Schilling, S. Buda, K. Tolksdorf, Zweite Aktualisierung der „Retrospektiven Phaseneinteilung der COVID-19- Pandemie in Deutschland“. (2022).

38. L. B. Aknin, *et al.*, Policy stringency and mental health during the COVID-19 pandemic: a longitudinal analysis of data from 15 countries. *Lancet Public Health* **7**, e417–e426 (2022).

39. D. Meintrup, M. Nowak-Machen, S. Borgmann, A Comparison of Germany and the United Kingdom Indicates That More SARS-CoV-2 Circulation and Less Restrictions in the Warm Season Might Reduce Overall COVID-19 Burden. *Life* **12**, 953 (2022).

40. S. Kuhlmann, J. and Franzke, Multi-level responses to COVID-19: crisis coordination in Germany from an intergovernmental perspective. *Local Gov. Stud.* **48**, 312–334 (2022).

41. C. Egger, R. Magni-Berton, “A Comparative Journey into COVID-19 Policies in Europe” in *Covid-19 Containment Policies in Europe*, C. Egger, R. Magni-Berton, E. de Saint-Phalle, Eds. (Springer Nature Switzerland, 2024), pp. 1–20.

42. M. Kolobova, D. Jdanov, D. Jasilionis, V. M. Shkolnikov, R. Rau, Variation in mortality burden of the COVID-19 pandemic across federal states in Germany. *Eur. J. Public Health* **33**, 930–936 (2023).

43. S. Volk, Political Performances of Control During COVID-19: Controlling and Contesting Democracy in Germany. *Front. Polit. Sci.* **3** (2021).

44. R. Koch-Institut, 7-Tage-Inzidenz der COVID-19-Fälle in Deutschland. Zenodo. https://doi.org/10.5281/zenodo.12631992. Deposited 3 July 2024.

45. F. D. Mann, *et al.*, A Novel Approach to Model Cumulative Stress: Area Under the s-factor Curve. *Soc. Sci. Med. 1982* **348**, 116787 (2024).

46. A. G. Blom, *et al.*, Mannheim Corona StudyMannheimer Corona-Studie. GESIS Data Archive. https://doi.org/10.4232/1.13700. Deposited 2021.

47. M. Witthöft, S. M. Jungmann, S. Germer, A.-K. Bräscher, Early Adverse Effects of Behavioural Preventive Strategies During the COVID-19 Pandemic in Germany: An Online General Population Survey. *Clin. Psychol. Eur.* **4**, e7205 (2022).

48. A. E. Teschendorff, C. E. Breeze, S. C. Zheng, S. Beck, A comparison of reference-based algorithms for correcting cell-type heterogeneity in Epigenome-Wide Association Studies. *BMC Bioinformatics* **18**, 105 (2017).

49. A. E. Teschendorff, S. C. Zheng, Cell-type deconvolution in epigenome-wide association studies: a review and recommendations. *Epigenomics* **9**, 757–768 (2017).

50. W. L. Do, *et al.*, Epigenome-wide meta-analysis of BMI in nine cohorts: Examining the utility of epigenetically predicted BMI. *Am. J. Hum. Genet.* **110**, 273–283 (2023).

51. R. Philibert, M. Dogan, S. R. H. Beach, J. A. Mills, J. D. Long, AHRR methylation predicts smoking status and smoking intensity in both saliva and blood DNA. *Am. J. Med. Genet. Part B Neuropsychiatr. Genet. Off. Publ. Int. Soc. Psychiatr. Genet.* **183**, 51–60 (2020).

52. C. H. Klatzka, *et al.*, “TwinLife Scales Manual. All data collections” (2023).

53. C. H. Klatzka, L. Paulus, F. Lenau, E. Hahn, “Documentation TwinLife Data: Height, Weight, and BMI. F2F2” (2021).

54. H. B. G. Ganzeboom, D. J. Treiman, Internationally Comparable Measures of Occupational Status for the 1988 International Standard Classification of Occupations. *Soc. Sci. Res.* **25**, 201–239 (1996).

55. A. D. Johnson, A. Martin, J. Brooks-Gunn, S. A. Petrill, Order in the House! Associations among Household Chaos, the Home Literacy Environment, Maternal Reading Ability, and Children's Early Reading. *Merrill-Palmer Q.* **54**, 445–472 (2008).

56. R. L. Spitzer, K. Kroenke, J. B. W. Williams, B. Löwe, A Brief Measure for Assessing Generalized Anxiety Disorder: The GAD-7. *Arch. Intern. Med.* **166**, 1092–1097 (2006).

57. A. T. Beck, R. A. Steer, G. K. Brown, BDI – FastScreen for Medical Patients. https://doi.org/10.1037/t14984-000. Deposited 8 October 2012.

58. R Core Team, R: A Language and Environment for Statistical Computing. (2020).

59. llll

60. H. Wickham, *Ggplot2: Elegant Graphics for Data Analysis* (Springer-Verlag New York, 2016).

61. T. L. Pedersen, patchwork: The Composer of Plots. https://doi.org/10.32614/CRAN.package.patchwork. Deposited 1 December 2019.

62. corrplot function - RDocumentation. Available at: https://www.rdocumentation.org/packages/corrplot/versions/0.95/topics/corrplot [Accessed 31 March 2025].

63. H. Wickham, *et al.*, Welcome to the tidyverse. *J. Open Source Softw.* **4**, 1686 (2019).

64. M. Dowle, A. Srinivasan, Data.Table: Extension of `data.Frame`. (2020).

65. K. Müller, H. Wickham, Tibble: Simple Data Frames. (2020).

66. D. D. Sjoberg, *et al.*, Gtsummary: Presentation-Ready Data Summary and Analytic Result Tables. (2021).

67. O. Cinar, W. Viechtbauer, The poolr Package for Combining Independent and Dependent p Values. *J. Stat. Softw.* **101**, 1–42 (2022).

68. J. Li, L. Ji, Adjusting multiple testing in multilocus analyses using the eigenvalues of a correlation matrix. *Heredity* **95**, 221–227 (2005).
